# Analyses of the Stability and Core Taxonomic Memberships of the Human Microbiome

**DOI:** 10.1371/journal.pone.0063139

**Published:** 2013-05-06

**Authors:** Kelvin Li, Monika Bihan, Barbara A. Methé

**Affiliations:** J Craig Venter Institute, Rockville, Maryland, United States of America; University of Illinois, United States of America

## Abstract

Analyses of the taxonomic diversity associated with the human microbiome continue to be an area of great importance. The study of the nature and extent of the commonly shared taxa (“core”), versus those less prevalent, establishes a baseline for comparing healthy and diseased groups by quantifying the variation among people, across body habitats and over time. The National Institutes of Health (NIH) sponsored Human Microbiome Project (HMP) has provided an unprecedented opportunity to examine and better define what constitutes the taxonomic core within and across body habitats and individuals through pyrosequencing-based profiling of 16S rRNA gene sequences from oral, skin, distal gut (stool), and vaginal body habitats from over 200 healthy individuals. A two-parameter model is introduced to quantitatively identify the core taxonomic members of each body habitat’s microbiota across the healthy cohort. Using only cutoffs for taxonomic ubiquity and abundance, core taxonomic members were identified for each of the 18 body habitats and also for the 4 higher-level body regions. Although many microbes were shared at low abundance, they exhibited a relatively continuous spread in both their abundance and ubiquity, as opposed to a more discretized separation. The numbers of core taxa members in the body regions are comparatively small and stable, reflecting the relatively high, but conserved, interpersonal variability within the cohort. Core sizes increased across the body regions in the order of: vagina, skin, stool, and oral cavity. A number of “minor” oral taxonomic core were also identified by their majority presence across the cohort, but with relatively low and stable abundances. A method for quantifying the difference between two cohorts was introduced and applied to samples collected on a second visit, revealing that over time, the oral, skin, and stool body regions tended to be more transient in their taxonomic structure than the vaginal body region.

## Introduction

The ability to identify and define the nature and extent of common or core microbial community membership versus those less prevalent within human body habitats across multiple individuals is a subject of significant interest and importance [1], [2], [3]. The presence of these shared taxa as defined by their ubiquity and abundance is critical for improving our understanding of microbial diversity and stability arrangements across space and time and establishing the frameworks for further understanding the roles that interconnected evolutionary, ecological and stochastic processes may play in shaping these patterns [4], [5].

Abundant and ubiquitous members of the microbial community may indicate those populations well-adapted to a particular ecological niche [6]. Within the cohort, members present in low abundance, ubiquity, or both, may contribute to the high interpersonal variation of the microbiome and represent a vast source of genetic material. The ecological roles of this low abundant fraction of the community however, are even less defined and may include a wide range of possibilities several of which are discussed here. Some of these members may exert minimal functional contributions to the community, for example, if they are dormant or transient (exhibit short residence time) [7]. However, increasing evidence suggests that the low abundant fraction may include metabolically active members that embody a range of ecological strategies [6]. For example, they could be stress tolerant organisms with low growth rates or metabolic specialists, and as such, represent possible sources within the human body that may contribute to commensalism, pathogenesis, or dysbiosis, upon shifts in the equilibrium of the microbial community [8], [9], [10]. Therefore, understanding what constitutes core and non-core taxa is essential to improving our knowledge of microbial ecology and links of taxonomy to metabolic function. This is critical for driving future research in directly testing the influences of evolution, ecology and stochastic processes on biogeographic patterns and facilitating the ability to use taxonomic profiles as possible diagnostic markers of health and disease states.

The NIH sponsored HMP has provided an unprecedented opportunity to examine the microbial diversity within and across body habitats and individuals, through pyrosequencing-based profiling of 16S rRNA gene sequences (16S) from oral, skin, distal gut, and vaginal body habitats from over 200 healthy individuals [11], [12], [13]. This has enabled the identification, evaluation, and examination of the shared microbial diversity among a cohort of healthy individuals and over time. Work by Huse et al. [2], commenced an analyses of the core microbiota present in HMP data sets by identifying these members at various prevalence levels (ubiquity) using a fixed abundance cutoff. In this current work, we build from these initial findings a more nuanced core analysis by advancing a probabilistic interpretation of core taxa for individual body habitats and their collection into body regions. This analysis exposes the continuous relationship between abundance and ubiquity; thus providing enhanced insight into the variation across the cohort's microbiome and the taxa present in the long tail of the rank abundance distribution. Through the inclusion of key graphical representations to help visualize and compare this additional complexity within and between body habitats, the concept of "minor core" taxa is introduced, identified, and supported. Within this probabilistic context, the temporal stability of the microbiome across the cohort is further explored through the examination of core taxa between subsequent donor sample collections.

The measurement of the shared taxa between two habitats can be made by utilizing one of several indices of shared similarity such as Jaccard [14], Sørensen [15], Morisita-Horn [16], Bray-Curtis [17], or UniFrac [18]. These indices are represented by a scalar real value between 0 and 1, where 1 is maximum similarity, when every taxon between the microbial community members have equal membership and abundance, and 0 refers to no overlap, whatsoever. While these measurements are useful for quantitatively comparing an arbitrary number of samples pairwise and generating distance (1-similarity) matrices for all sample analyses, to define a core microbiome by identifying which specific taxa are shared across all habitats simultaneously requires an alternative method that will quantitatively elect specific taxa for membership.

The large and continuous differences in magnitude between the most and least abundant taxa require a consideration of both ends of this wide spectrum. The interaction of the multitude of organisms in a habitat, and the variation inherent to both sampling and sequencing depth, precludes a presence/absence, or all/nothing approach, which is frequently used to generate simple Venn-style diagrams [19]. Thus, “in-or-out” set-based enumerations of shared taxa are misleading if they do not take into account the effects of sampling depth on the detection of low abundant taxa. Furthermore, platform-dependent sequencing errors will also affect the taxonomic classification of reads, potentially leading to spurious OTUs and inflated measurements of diversity, thus making direct comparisons between studies difficult. The detection of extremely low abundant taxa, i.e., organisms whose detection are barely at or below the sampling depth of the sequencing methodology, require qualification with a condition defined probabilistically. For example, the presence/absence classification of a taxon of interest can be qualified as the probability of detecting that organism upon resampling and sequencing to the same coverage depth. This coverage depth could be standardized if multiple depths were used across the samples being compared, and the use of bootstrapping is commonly applied to simulate this. Ultimately, a taxon would be included as “in” if it could be asserted that its presence would be detected in ≥95% of the bootstrap simulated samples. See “Materials and Methods” for an explanation of how the binomial distribution can be utilized to make this assertion without resorting to computational bootstrapping.

An escape from the simplified Boolean restriction of defining a core microbiome is rewarded with a richer view of the taxonomic complexity found within a cohort. In particular, the relationship between taxonomic abundance and ubiquity for each taxon reveals characteristics that may be used to distinguish between taxa across a cohort, as well as body habitats from each other. The term “abundance” can be defined as the proportion that a taxon of interest exists in a specific donor’s sample. The abundance of a taxon is computed by normalizing the taxonomic counts across the 16S profile for a single sample, such that their normalized values sum to 1, thus generating a set of proportions, i.e., compositional data. Defining an abundance cutoff would then establish a lower bound threshold for set inclusion (“in”), thus converting each taxon’s proportion to a Boolean value. The term “ubiquity” can be defined as the proportion of the cohort that a taxon of interest may be detected in. The detection criterion would then be delineated by a taxon exceeding the defined abundance cutoff for a specific donor. Thus, for a specific taxon, the ubiquity would be the proportion of donors with that taxon exceeding the abundance cutoff. Finally, to define a “core”, a ubiquity cutoff would be delineated. For a specific taxon, if the ubiquity exceeds the defined cutoff, then that taxa would be considered a member of the core. Thus, applying both the abundance and ubiquity (two-parameter) cutoffs across each taxa for the cohort would construct a set of taxa, defined as the “core” taxa, for the cohort.

A method of incrementally removing low abundant OTUs, such as that performed by MultiCoLa [20], discards the proportion of taxa associated with low read counts. This distorts the compositional relationship of remaining taxa among samples (subcompositional coherence) [21] since each sample may have a different proportion of counts removed, depending on the tail length of each sample's rank abundance distribution. When comparing categorical data, for example with a χ^2^ test, low count categories are generally conserved into a newly created low abundance category [22] and degrees of freedom are reduced accordingly. As such, the two-parameter model avoids the truncation of entire segments of data, allowing the taxonomic proportions to be analyzed with subcompositional coherence even when considering taxa only at high levels of abundance.

A plot of ubiquity versus abundance for each taxon is used to help visualize the relationship between ubiquity and abundance across the entire spectrum of abundances and across all the taxa for a given cohort. When all ubiquity versus abundance lines (Ub-Ab lines) are plotted simultaneously (Ub-Ab plot), the visualization helps to elucidate the relationship between ubiquity and abundance for the cohort’s microbial community. When plotted with other Ub-Ab curves for taxa in the same cohort, context is provided for comparing the abundance levels at which taxa exist and their variation across the donors. As will be demonstrated and discussed in the Results and Discussion section, the relationship between ubiquity and abundance varies between taxa and is dependent upon the habitat from which the samples were collected. There is not a simple linear relationship between ubiquity and abundance, although by definition, the ubiquity is a monotonically decreasing function as abundance increases. As such, it can be considered a cumulative distribution function (cdf), however with a domain from 1 to 0, thus decreasing in y (ubiquity), as x increases (abundance). The differences that can be seen between the various Ub-Ab curves for each taxon is attributed to the variance of abundance among the donors, which depends on the taxon, body region of study, and the cohort.

This relationship between ubiquity and abundance appears to be conserved across related body habitats for the same taxon, making it possible to identify a signature representing the underlying microbial community’s structure. This characteristic is the focus of the research presented in this study. The variation of taxonomic abundance by body habitat is also explored, confirming that the identified cores are stable (i.e. low variation in taxonomic abundance among cohort samples within a body habitat). These methods also identify taxa that are saliently stable or unstable across the healthy cohort, making them potential marker candidates for different roles within the community, including contributions to metabolic processes that may enhance health or disease. In particular, these analyses have elucidated a contingent of stable, low abundant taxa that are in a majority of the cohort which we identify as members of the “minor” core. Pooled analyses of 16S profiles based on either taxonomy-dependent, or taxonomy-independent data, would have overlooked this contingent, since an averaging of abundances across the cohort would have obfuscated their characteristics.

The two-parameter model can be further exploited by comparing the nature and relationship of complexity between two cohorts. This methodology differs from the index-based approach, in that instead of summarizing the differences between pooled cohorts with a numerical (1-similarity) index, (where similarity is measured with one of: Jaccard, Sørensen, Morisita-Horn, etc.) specific differences, both taxonomically and in magnitude, can be identified within the context of other members.

In this study, we present results extending previous investigations of human microbiome core membership as revealed across body habitats and regions, from 16S profiles generated by the HMP. First, the core members of genera-based and OTU-based profiles are enumerated across the body habitats by introducing the two-parameter model and visualized with the Ub-Ab plot. Next, the minor core is revealed across the various body habitats and their characteristics in the Ub-Ab plots are explained. The variation of taxon abundance across the cohort is explored for each body habitat, reinforcing the appropriateness of the core election criteria and their distinction from other taxa. An interpretation of the two-parameter model for body regions, the composite of individual body habitats, is described and then applied to genera-based taxonomic profiles, to identify body region core taxa members. Finally, a comparison is made between the microbiota of two cohorts with the introduction of the Ubiquity-Ubiquity (U-U) plot. This provides a graphical representation of the differences between the two-parameter representations of two cohorts, and a test statistic named, the “abundance-weighted” Kolmogorov-Smirnov (AWKS) statistic [23] is introduced to quantify the magnitude of the differences. This test statistic is demonstrated to be useful for statistical inference by its application to the comparison of first visit to second visit samples for all body habitats on both genera and OTU-based taxonomic profiles.

## Results and Discussion

### The Core Genera

Core taxonomic members of the microbiome for each body habitat were defined by the following pairs of abundance versus ubiquity cutoffs (abundance | ubiquity):

a.) 10% | 75%

b.) 1% | 80%

c.) 0.1% | 85%

d.) 0.01% | 90%

**Figure 1:**
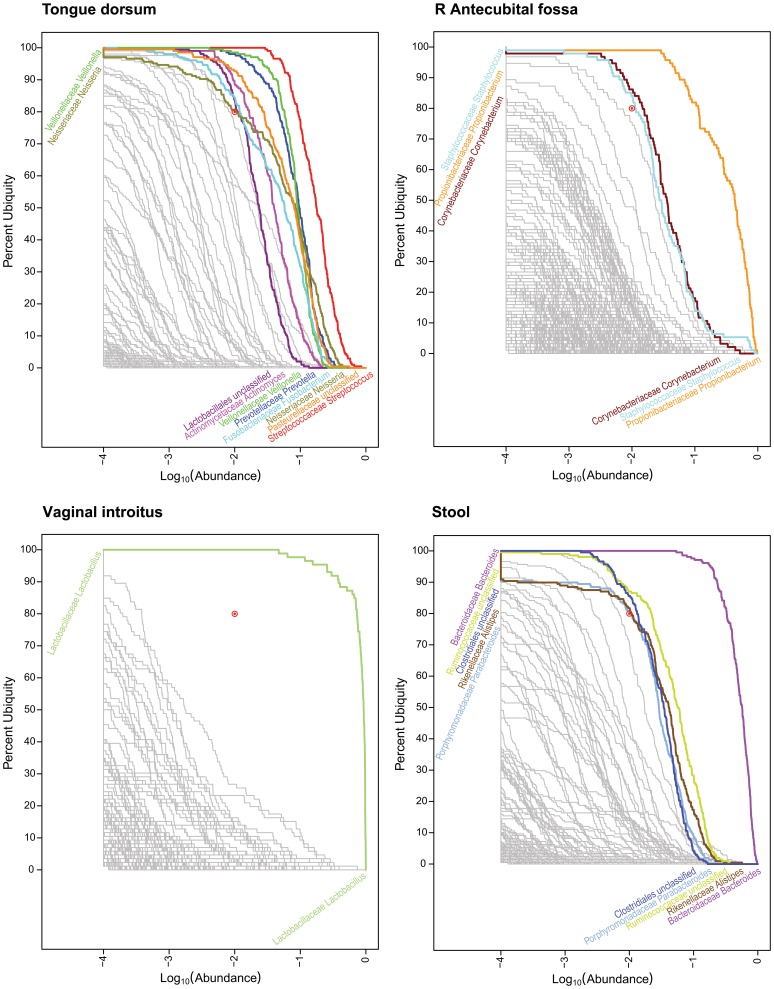
Genera Ub-Ab Plots for 4 Body Habitats. The taxonomic abundance is represented along the x-axis and the ubiquity across the cohort is represented along the y-axis. Abundance and ubiquity increase away from the origin on the bottom left. Based on the sequencing depth of the samples, a cutoff of 1 part per 10,000, or log_10_(0.0001) = −4 was selected as the plotted lower limit, found on the left-hand side of each Ub-Ab plot. For each plot, the location of the two-parameter cutoff is marked with a red bull’s eye (). Taxa with curves above the bull’s eye are colored and labeled; however those below since they are very numerous and overlapping, are greyed out and left unlabeled to reduce clutter.

These logarithmically decreasing abundances were selected to illustrate the rate at which the enrollment of the core membership would grow as lower abundant organisms were considered. Due to the inverse relationship between abundance and ubiquity, for each of the 4 combinations studied, the ubiquity cutoff was incrementally increased from 75% to 90% in order to maintain a manageable number of taxa to review. These numbers may be examined in Table 1 (Number of Core Genera and OTUs at Selected Ubiquity and Abundance Cutoffs). As demonstrated in Figure 1 (Genera Ub-Ab Plots for 4 Body Habitats), there is a not a single pair of cutoffs that can define core for all body habitats illustrating precisely why confidence intervals (CIs) must be provided for core number estimates. CIs for each of the core computes were estimated with bootstrapping; resampling with replacement from both the distribution of taxonomic classifications from each donor, and the donors included. The majority of the 95% confidence intervals were only within 1 or 2 members away from the median core value. “Observed” core member numbers were calculated directly on the 16S profiles without bootstrapping, thus representing the core member numbers calculated on the observed, unperturbed set of donors and read counts. The majority of median and observed core member counts were identical, but both values were included so that the table could be reconciled with the Ub-Ab plots, that represent the cohort’s observed 16S profile.

**Table 1:** The Number of Core Genera and OTUs (in parentheses) at Selected Ubiquity and Abundance Cutoffs.

Ubiquity	75%				80%				85%				90%			
Abundance	10%				1%				0.10%				0.01%			
Log_10_(Abundance)	−1				−2				−3				−4			
	Observed	Median	LB	UB	Observed	Median	LB	UB	Observed	Median	LB	UB	Observed	Median	LB	UB
**Oral**																
Buccal mucosa	1 (1)	1 (1)	1 (1)	1 (1)	3 (4)	3 (4)	3 (3)	3 (4)	11 (10)	11 (10)	9 (9)	12 (11)	15 (14)	14 (12)	12 (10)	15 (14)
Hard palate	1 (1)	1 (1)	1 (1)	1 (1)	6 (7)	6 (7)	5 (6)	7 (7)	14 (16)	14 (15)	13 (13)	15 (17)	18 (22)	17 (18)	15 (16)	19 (20)
Keratinized gingiva	2 (1)	2 (1)	2 (1)	2 (1)	2 (2)	2 (2)	2 (2)	2 (3)	5 (5)	5 (6)	4 (5)	6 (7)	7 (8)	7 (7)	5 (6)	8 (8)
Palatine Tonsils	0 (0)	0 (0)	0 (0)	1 (0)	5 (6)	5 (6)	5 (5)	7 (6)	14 (15)	14 (15)	14 (14)	15 (17)	20 (20)	17 (18)	16 (17)	18 (20)
Saliva	0 (0)	0 (0)	0 (0)	0 (0)	7 (7)	7 (7)	6 (7)	7 (8)	20 (21)	19.5 (20)	18 (18)	23 (22)	26 (28)	26 (25)	24 (22)	27 (27)
Subgingival plaque	0 (0)	0 (0)	0 (0)	0 (0)	6 (2)	6 (2)	5 (2)	6 (3)	15 (12)	15 (12)	13 (10)	17 (14)	23 (19)	21 (17)	18 (14)	23 (19)
Supragingival plaque	0 (0)	0 (0)	0 (0)	0 (0)	6 (4)	7 (4)	6 (3)	8 (5)	17 (17)	16 (17)	15 (16)	18 (18)	22 (23)	20 (21)	19 (19)	21 (23)
Throat	1 (0)	1 (0)	1 (0)	1 (0)	7 (6)	6.5 (6)	5 (5)	8 (7)	14 (16)	14 (16)	13 (14)	15 (17)	18 (20)	16 (18)	15 (16)	19 (21)
Tongue dorsum	1 (0)	1 (0)	0 (0)	1 (0)	8 (8)	7 (8)	6 (7)	8 (9)	13 (14)	13 (14)	12 (13)	14 (15)	18 (19)	16 (17)	14 (15)	18 (20)
**Skin**																
Anterior nares	2 (2)	2 (2)	1 (0)	2 (2)	3 (3)	3 (3)	3 (3)	3 (3)	4 (4)	4 (4)	4 (3)	4 (4)	5 (4)	4 (4)	4 (4)	5 (4)
L Antecubital fossa	1 (1)	1 (1)	1 (1)	1 (1)	2 (1)	2 (1)	1 (1)	3 (2)	4 (4)	4 (4)	4 (4)	6 (6)	6 (4)	5 (4)	4 (4)	6 (6)
L Retroauricular crease	1 (1)	1 (1)	1 (1)	1 (1)	2 (2)	2 (2)	2 (2)	2 (2)	3 (2)	3 (2)	2 (2)	3 (3)	4 (3)	3 (3)	2 (2)	4 (4)
R Antecubital fossa	1 (1)	1 (1)	0 (0)	1 (1)	3 (2)	3 (2)	2 (1)	4 (3)	5 (4)	5 (4)	5 (3)	6 (6)	5 (6)	5 (5)	4 (3)	6 (7)
R Retroauricular crease	1 (1)	1 (1)	1 (1)	1 (1)	2 (2)	2 (2)	2 (2)	2 (2)	2 (2)	2 (2)	2 (2)	3 (2)	3 (3)	3 (2)	2 (2)	3 (3)
**Vaginal**																
Mid vagina	1 (1)	1 (1)	1 (1)	1 (1)	1 (1)	1 (1)	1 (1)	1 (1)	1 (1)	1 (1)	1 (1)	1 (1)	1 (1)	1 (1)	1 (1)	1 (1)
Posterior fornix	1 (1)	1 (1)	1 (1)	1 (1)	1 (1)	1 (1)	1 (1)	1 (1)	1 (1)	1 (1)	1 (1)	1 (1)	1 (1)	1 (1)	1 (1)	2 (1)
Vaginal introitus	1 (1)	1 (1)	1 (1)	1 (1)	1 (1)	1 (1)	1 (1)	1 (1)	1 (1)	1 (1)	1 (1)	1 (1)	2 (1)	1 (1)	1 (1)	2 (1)
**Stool**	1 (1)	1 (1)	1 (0)	1 (1)	5 (4)	4 (3)	3 (2)	5 (4)	7 (6)	7 (6)	6 (5)	9 (7)	9 (8)	8 (8)	6 (6)	10 (9)

Observed core membership counts are based on unperturbed 16S profiles.

Median, LB, and UB core membership counts were computed using bootstrapping with 160 iterations and 95% CI.

The number of core genera at 10% | 75% ranged from 0 (palatine tonsils, saliva, subgingival plaque, and supragingival plaque) to 2 (keratinized gingiva and anterior nares). However the majority of body habitats only consisted of 1 core genus. At 1% | 80%, the number of identified core taxa increased more rapidly for oral and stool regions, while vaginal regions remained constant at 1 core genus. This pattern of either rapid or almost no increase in core genera followed suit through the cutoffs of 0.1% | 85% and 0.01% | 90%, as well. At 0.01% | 90%, the vaginal body habitats of mid vagina and posterior fornix, maintained a core genus count of 1, but saliva and subgingival plaque reached significantly higher core genera counts of twenty-six and twenty-three, respectively.

The core genera members for 1% | 80% and 10% | 75% are named in Table 2 (Core Family and Genera at 10% | 75% and 1% | 80%). The core genera at 10% | 75% were a subset of the core for those identified at 1% | 80%, and have been identified with bullet glyphs on the left-hand side of the names in Table 2 (The Number of Core Genera and OTUs at Selected Ubiquity and Abundance Cutoffs). At the cutoff of 10% | 75%, *Streptococcus* was revealed as a core member in the body habitats of buccal mucosa, hard plate, keratinized gingiva, throat and tongue dorsum. Keratinized gingiva also had a second core member of *Pasteurellacaeae unclassified*. (See “The Core OTUs” section for how the core *Pasteurellacaeae unclassified* was divided into OTUs.) All skin habitats maintained *Propionibacterium* in their core, however *Corynebacterium* was also present when anterior nares was examined. The anterior nares habitat is dissimilar to the other 4 skin habitats, in that it is adjacent to the mucosal membrane of the nasal cavities. Across the 3 vaginal habitats, *Lactobacillus* was consistently found in each respective core. For the stool habitat, only *Bacteroides* was considered a core member.

**Table 2:** The Core Genera and OTUs at 1% | 80% and 10% | 75%.

Oral		Oral (continued)	
* Buccal mucosa*	• Streptococcaceae Streptococcus (2)	* Tongue dorsum*	• Streptococcaceae Streptococcus (2, 6)
	Pasteurellaceae unclassified (16, 19)		Veillonellaceae Veillonella (4)
	Staphylococcaceae Gemella (11)		Prevotellaceae Prevotella (10)
* Hard palate*	• Streptococcaceae Streptococcus (2, 6)		Pasteurellaceae unclassified (16)
	Pasteurellaceae unclassified (16)		Actinomycetaceae Actinomyces (14)
	Veillonellaceae Veillonella (4)		Fusobacteriaceae Fusobacterium (9)
	Prevotellaceae Prevotella (10)		Lactobacillales unclassified (13)
	Lactobacillales unclassified (13)		Neisseriaceae Neisseria (8)
	Staphylococcaceae Gemella (11)		
* Keratinized gingiva*	• Streptococcaceae Streptococcus (2)	**Skin**	
	• Pasteurellaceae unclassified (19)	* Anterior nares*	• Propionibacteriaceae Propionibacterium (1)
* Palatine Tonsils*	Streptococcaceae Streptococcus (2, 6)		• Corynebacteriaceae Corynebacterium (12)
	Veillonellaceae Veillonella (4)		Staphylococcaceae Staphylococcus (5)
	Prevotellaceae Prevotella (10)	* L Antecubital fossa*	• Propionibacteriaceae Propionibacterium (1)
	Fusobacteriaceae Fusobacterium (9)		Corynebacteriaceae Corynebacterium (−)
	Pasteurellaceae unclassified (16)	* L Retroauricular crease*	• Propionibacteriaceae Propionibacterium (1)
* Saliva*	Prevotellaceae Prevotella (10)		Staphylococcaceae Staphylococcus (5)
	Streptococcaceae Streptococcus (2, 6)	* R Antecubital fossa*	• Propionibacteriaceae Propionibacterium (1)
	Veillonellaceae Veillonella (4)		Corynebacteriaceae Corynebacterium (−)
	Pasteurellaceae unclassified (16)		Staphylococcaceae Staphylococcus (5)
	Fusobacteriaceae Fusobacterium (9)	* R Retroauricular crease*	• Propionibacteriaceae Propionibacterium (1)
	Porphyromonadaceae Porphyromonas (7)		Staphylococcaceae Staphylococcus (5)
	Neisseriaceae Neisseria (−)		
* Subgingival plaque*	Streptococcaceae Streptococcus (2)	**Vaginal**	
	Fusobacteriaceae Fusobacterium (9)	* Mid vagina*	• Lactobacillaceae Lactobacillus (3)
	Flavobacteriaceae Capnocytophaga (−)	* Posterior fornix*	• Lactobacillaceae Lactobacillus (3)
	Prevotellaceae Prevotella (−)	* Vaginal introitus*	• Lactobacillaceae Lactobacillus (3)
	Corynebacteriaceae Corynebacterium (−)		
	Pasteurellaceae unclassified (−)	**Stool**	• Bacteroidaceae Bacteroides (27, 17, 31)
* Supragingival plaque*	Streptococcaceae Streptococcus (2)		Ruminococcaceae unclassified (−)
	Flavobacteriaceae Capnocytophaga (−)		Clostridiales unclassified (−)
	Corynebacteriaceae Corynebacterium (15)		Rikenellaceae Alistipes (30)
	Pasteurellaceae unclassified (−)		Porphyromonadaceae Parabacteroides (−)
	Neisseriaceae unclassified (21)		
	Fusobacteriaceae Fusobacterium (9)		
* Throat*	• Streptococcaceae Streptococcus (2, 6)		
	Veillonellaceae Veillonella (4)		
	Prevotellaceae Prevotella (10)		
	Pasteurellaceae unclassified (16)		
	Actinomycetaceae Actinomyces (−)		
	Fusobacteriaceae Fusobacterium (9)		
	Lachnospiraceae unclassified (−)		

Corresponding core OTUs underlying the taxonomy are labeled in parentheses.

A bullet (•) identifies core family and genera at >75% ubiquity and >10% abundance.

At 1% | 80%, the number of core members in oral and stool habitats increased several fold. Of the 9 oral habitats, *Pasteurellaceae unclassified* and *Streptococcus* were identified as core genera among them all. *Fusobacterium* and/or *Prevotella* were core members in 6 out of the 9 oral habitats, and *Veillonella* was found in 5 habitats. At this lower abundance cutoff, the skin regions only introduced *Corynebacterium* or *Staphylococcus*. Vaginal habitats did not increase their core size, and stool included *Ruminoccacae*, *Clostridiales*, *Alistipes* and *Parabacteroides* in its core.

To visualize the relationship between ubiquity and abundance for each taxon, it is useful to plot curves for each taxa as a means of both identifying the starting ubiquity, at the lowest abundance, and characterizing the drop-off of ubiquity, as the abundance cutoff is increased. When all taxa curves are plotted simultaneously in a single figure, context is provided among taxa and the viewer is able to quickly identify taxa of interest. These plots will be referred to as Ubiquity-Abundance (Ub-Ab) plots (Figure 1, Genera Ub-Ab Plots for 4 Body Habitats). For an environment rich in taxonomic diversity, it has been seen that the drop off of taxonomic abundance follows a power-law probability distribution, thus the majority of taxa have vanishing low proportions when below 10% in abundance. Performing the log_10_ transform on the abundances (x-axis) is critical, since it essentially provides a visual magnification of these low abundant taxa. Body habitats from the same body region tend to have a similar collection of curves, with respect to their clustering, slopes and degrees of inflection. Collectively, these qualitative characteristics which can be discerned with only visual inspection will be referred to as the “signature” for a cohort’s microbiome.

Four body habitats, one from each body region, were selected to demonstrate how the characteristic drop offs of ubiquity for each taxon provide a unique signature for each body habitat (Figure 1, Genera Ub-Ab Plots for 4 Body Habitats). The differences between body habitats from the same body region were less visually distinctive. The 4 Ub-Ab plots were generated and marked with a ubiquity and abundance cutoff at 80% and 1%, respectively. The number of, and labels for, each taxa curve above each of the bull’s eye, are the same counts and named taxa for the corresponding body habitats in Table 1 (The Number of Core Genera and OTUs at Selected Ubiquity and Abundance Cutoffs) and Table 2 (The Core Genera and OTUs at 10% | 75% and 1% | 80%). The Ub-Ab plot for the oral habitat of tongue dorsum (top left subplot), reveals that there is a continuum of ubiquity and abundance cutoffs that arguably could have been chosen to define the core taxa of the microbiome, especially since the curves may cross each other. The Ub-Ab curves for two taxa will cross each other when the more ubiquitous taxon does not maintain its level of ubiquity above the less ubiquitous taxon at a greater abundance level. For example, this occurs when a highly ubiquitous but low abundant taxon is compared against a low ubiquity taxon that is highly abundant in the subset of donors it is found in. The lack of a distinctive grouping of Ub-Ab taxon curves makes the choice of an ideal two-parameter cutoff less prescribed, thus making the decision between a focus on greater ubiquity or greater abundance, more subjective. Examination of the skin habitat of the right (R) antecubital fossa (top right subplot), reveals that by significantly reducing the abundance cutoff of the two-parameter model from 1% to 0.1% (−2 and −3, respectively, in log_10_ scale), only 3 more taxa would have been added to the core microbiome. A compression of taxon Ub-Ab curves can be observed below 80% ubiquity, revealing that a majority of the sampled cohort share many taxa, but at a low abundance. The bottom left subplot, the vaginal introitus body habitat, reveals that a single genus of *Lactobacillus* is the clearest core member. It is significantly abundant and ubiquitous across all donors. For this body habitat, the two-parameter cutoff could have been defined across a large area of ubiquity and abundance cutoffs, and yet still revealed the single core genus. The last Ub-Ab plot on the bottom right is for the stool habitat. It has characteristics of the vaginal region, in the sense that the ubiquity and abundance of a single genus, *Bacteroides*, is clearly apparent. However, it also has characteristics of the skin region in that a second tier of core microbiome members could be defined by maintaining the ubiquity cutoff at 80%, while reducing the abundance cutoff by a single order of magnitude.

While each body region had very distinct sets of Ub-Ab curves, the various body habitats, if grouped by body regions, were relatively similar. This indicates that the characteristics necessary to group individual samples by habitat are well-conserved in this representation of cohort taxonomic structure. Body regions can be readily discerned from each other visually, but at the body habitat level, a more quantitative approach, which is introduced in “Comparing the microbiota of two cohorts”, may be necessary to determine the statistical significance of differences between two groups of samples. The analysis of the core members of the microbial communities based on taxonomy for body regions is discussed later. The Ub-Ab output for all 18 body habits is available as Supplemental Material, File S1 (Ub-Ab Plots 18 Body Habitats).

### The Core OTUs

The core OTUs were also computed on the 18 body habitats. Their values are listed in Table 1 (The Number of Core Genera and OTUs at Selected Ubiquity and Abundance Cutoffs), in parentheses, next to the genera counts. At 10% | 75% and 1% | 80%, the number of core genera and OTUs were mostly in agreement, and it was confirmed that when a genus was shared across a body region, the underlying OTU(s) were shared, as well. The core OTU identifiers for each body habitat were annotated next to their respective genera in Table 2 (The Core Genera and OTUs at 1% | 80% and 10% | 75%). With the exception of *Streptococcus*, OTUs #2 and #6, and *Pasteurellaceae* unclassified, OTUs #16 and #19, the core genera that were present across the body habitats could be represented by a single core OTU. At 1% | 80%, there were many cases in oral, skin, and stool body regions, where the OTUs underlying a core genus were no longer considered core OTUs because their distribution across the cohort was no longer ubiquitous. In Table 2 (The Core Genera and OTUs at 1% | 80% and 10% | 75%), these genera were marked with a hyphen within the parentheses. Nonetheless, in general the two approaches were in overall agreement.

**Figure 2:**
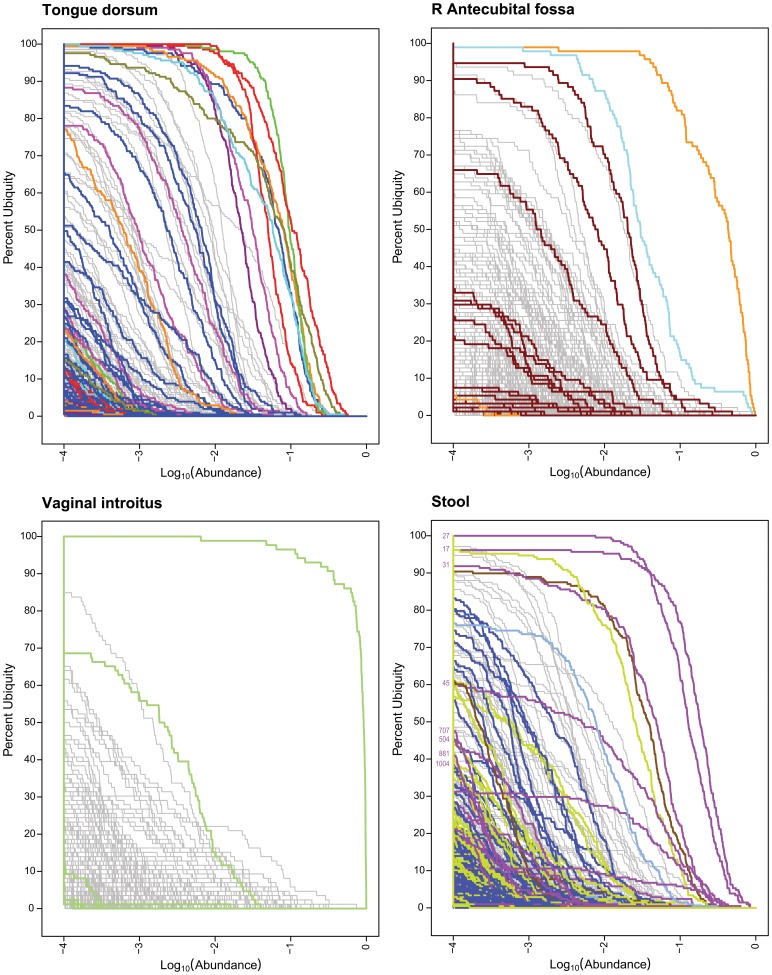
OTU Ub-Ab Plots for 4 Body Habitats. These Ub-Ab plots were generated based on OTUs. The colored curves are based on the same colors used for the core genera in Figure 1. Lines with the same color belong to the same genera.

The core OTUs for all body habitats are better analyzed in the context of the genera that they compose. Core genera at 1% | 80% were assigned colors for tongue dorsum, R antecubital fossa, vaginal introitus and stool in Figure 1 (Genera Ub-Ab Plots for 4 Body Habitats). The same colors were applied to the OTUs in Figure 2 (OTU Ub-Ab Plots for 4 Body Habitats).

In the tongue dorsum, the 8 core genera comprise many OTUs with various ubiquities. *Prevotella* was divided into the most OTUs, of which 6 were still present in more than 50% of the cohort. *Streptococcus* split into multiple OTUs, two of which (OTUs #2 and #6) still exceeded the 1% | 80% cutoff. The remaining 6 core genera were also composed of multiple OTUs, but in each case, a dominant underlying OTU remained ubiquitous and abundant enough to be a core representative.

Within the R antecubital fossa’s 3 core genera, only *Corynebacterium* divided into multiple OTUs, of which 3 were present at greater than 50% ubiquity. The division of *Corynebacterium* resulted in its loss from core membership at 1% | 80%, thus reducing the number of core OTUs for R antecubital fossa down to only 2.

Two *Lactobacillus* OTUs (#3 and #148) with ubiquity >50% were generated by the splitting of the single *Lactobacillus* core genus. The more ubiquitous *Lactobacillus* OTU #3 still maintained 100% ubiquity at an abundance of greater than 10^−4^. From the Ub-Ab plot, additional *Lactobacillus* OTUs could be found in the cohort, but their ubiquity and abundance were significantly less than these two most ubiquitous OTUs. Since there were a large number of *Lactobacillus* reads across all the vaginal samples, one might expect a proportionately larger measured OTU diversity due to spurious under-clustering because of sequencing error. The Ub-Ab plot is able to place this potentially spurious diversity in better context since they would not only be low in abundance, but also low in ubiquity.

The splitting of core genera into OTUs was the most dramatic within stool. *Bacteroides* split into 4 OTUs with ubiquity greater than 50%, of which 3 exceeded the 1% | 80% cutoff. Both *Alistipes* and *Ruminococcacae* (Family) maintained their membership in the OTU-based core. *Parabacteroides* dropped out of membership although only missing these cutoffs slightly. Due to the high ubiquity and abundance of *Bacteroides* OTUs in the stool habitat, further investigation was made to determine if it would be possible to associate species-level classifications to the OTUs and/or identify novel species. This is discussed in the next subsection, “Bacteroides in the Stool Habitat”.

Table 1 (The Number of Core Genera and OTUs at Selected Ubiquity and Abundance Cutoffs) also included the number of core OTUs that were identified in parentheses next to the core genera count for each body habitat. In spite of the much greater numbers of taxonomic units in OTU-based analyses, the number of core OTUs did not differ significantly from the number of core genera. Across all (abundance | ubiquity) cutoffs, the median difference was 0. The maximum difference between the counts of genera and OTUs was 4 in favor of genera for subgingival plaque (1% | 80%) and 4 in favor of OTUs for hard palate and subgingival plaque (0.01% | 90%). With a mean difference of 0, there was not a systematic bias across the differences towards either OTUs or genera.

These findings reveal that a very small subset of taxa, whether measured in genera or OTUs, in proportion to the total richness detected in a sample, can characterize a body region very accurately across the majority of the population. However, without the detection of body habitat specific core OTUs, the differentiation between body habitats is a more difficult problem, as they represent a finer resolution of physical location and physicochemical characteristics which may not significantly discriminate between them. By using a two-parameter model for defining a core, a quantitative differentiation can be made between taxa that are not widespread in a population, i.e., not ubiquitous, versus those in low abundance but very widespread, i.e. very ubiquitous, or common. An alternative analysis where all donors are pooled together by averaging normalized abundances and then analyzed for overall composition would not be able to identify these important differences.

#### Bacteroides in the stool habitat

**Figure 3:**
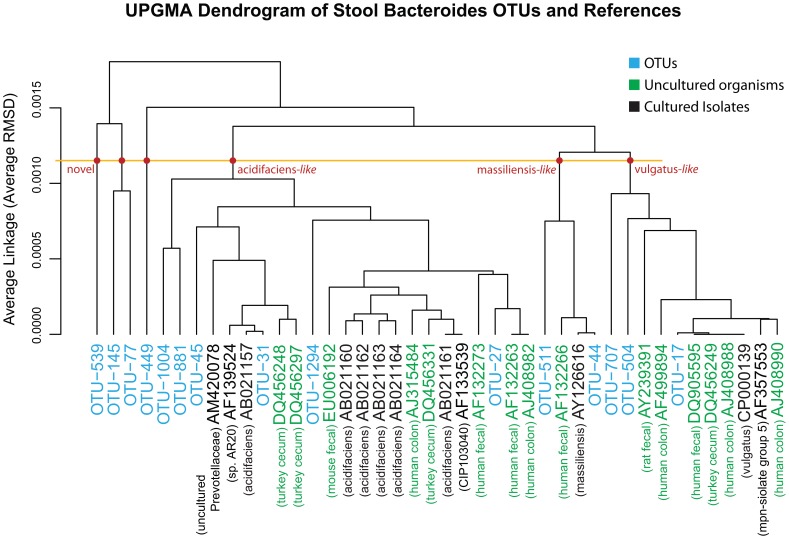
UPGMA Dendrogram of Stool Bacteroides OTUs. This is a UPGMA (average-linkage) dendrogram based on a distance matrix computed by using ANDES to estimate the RMSD between the nucleotide distributions of two groups of aligned sequences. The OTUs identified in the stool samples are colored blue. The reference sequences are labeled according to their Genbank ID and color-coded: green for the uncultured organisms and black for the cultured isolates. A short description for each reference sequence is labeled below the Genbank ID. A horizontal line was drawn to mark the possible cutoff for the RMSD difference separating species. The three novel branches and the three species-associated branches (*acidifaciens*-like, *massiliensis*-like and *vulgatus*-like) are marked with red dots.

To arrive at a deeper understanding of the variety of Bacteroides OTUs detected in Stool, a UPGMA tree was generated by building and comparing ANDES [24] profiles for each OTU between one another as well as with profiles generated for the reference sequences. Briefly, each ANDES profile contains the position-by-position nucleotide probability distribution for a group of sequences, e.g. an OTU, based on its underlying multiple sequence alignment (MSA). With this representation, the dissimilarity between two OTUs, independent of cluster size and necessity to identify a single representative sequence, can be computed by measuring the averaged root mean squared deviation (RMSD), across all positions. The smaller the RMSD between two ANDES profiles, the more similar they are. The reference sequences used to annotate the UPGMA tree were based on the 16S sequences from Silva [25]. A more sophisticated identification of evolutionary relationships using a phylogenetic tree building algorithm was not utilized to avoid the necessity of selecting a single representative sequence for each OTU. If a single sequence was selected to represent the OTU, it might not be an accurate representative of the cluster or its centroid, since the OTU might be composed of a distribution of strains along with noise from sequencing error. For the purposes of identifying the closest reference sequence to an OTU when treated as a whole, this distance-based approach, comparing the average of each OTU’s underlying sequences, was considered sufficient (Figure 3, UPGMA Dendrogram of Stool Bacteroides OTUs). Twenty-seven reference 16S sequences from the Silva database were selected based on the underlying matching reads of the OTUs using Mothur [26]. Of the twenty-seven reference 16S sequences, fifteen were labeled as uncultured bacterium that were isolated from turkey cecum, mouse fecal, human colon, human fecal, or rat fecal samples. The matching cultured isolates included the species of B. acidifaciens, B. massiliensis and B. vulgatus. The reference sequence AM420078, isolated from an oral sample putatively associated with Prevotellaceae, may have been mislabeled. The closest reference associations of OTUs #31, #44, and #17, were B. acidifaciens (AB021157), B. massiliensis (AY126616), and B. vulgatus (CP000139), respectively. From the UPGMA dendrogram, the closest cultured isolates to the top 4 most ubiquitous OTUs (#27, #17, #31, and #45) were all members of the Bacteroides and are listed as B. acidifaciens (AB021160-4), B. vulgatus (CP000139), B. acidifaciens (AB021157) and B.acidifaciens (AB021157), respectively. At an average RMSD of 0.0010, the species of B.acidifaciens, B. massiliensis, and B. vulgatus, were separated into non-overlapping clusters. The OTUs #539, {#145 and #77}, and #449 may be considered novel species, due to their more distinctive clustering away from the remaining reference sequences and associated OTUs. These 4 OTUs joined the 3 known species clusters at an RMSD exceeding 0.00125. The most ubiquitous stool Bacteroides OTUs are labeled in Figure 2 (OTU Ub-Ab Plots for 4 Body Habitats, Stool Panel). None of the novel OTUs were in a majority of the cohort, although #881 and #1004 were more ubiquitous than the other potentially novel OTUs.

### Minor Core: Low in Abundance, However Present in a Majority of the Population

Thus far, the definition of a core microbiome has been focused on high ubiquity taxa in conjunction with a characteristic of high abundance such that the significance of both measurements can be made with a sufficient degree of confidence across and within samples. This has led to a more conventional view of a core in that the taxa that have been identified could also have been discovered with a smaller cohort size and with a sequencing depth not necessarily exploiting the full capabilities of next generation sequencing.

With the two-parameter model, an alternative contingent of the microbiome can be elucidated by inverting the abundance restriction. In particular, instead of identifying core taxa as sufficiently “ubiquitous” at >75% of the cohort, let the definition of a “majority” of the population be a more loosely required ubiquity at >50% of the cohort and the definition of low abundance be <1%. For these alternative parameters to be meaningful, a large enough donor sample size and next generation sequencing depths for each body habitat are necessary, so that the detection of low abundant taxa may be considered non-spurious across the donors.

**Figure 4:**
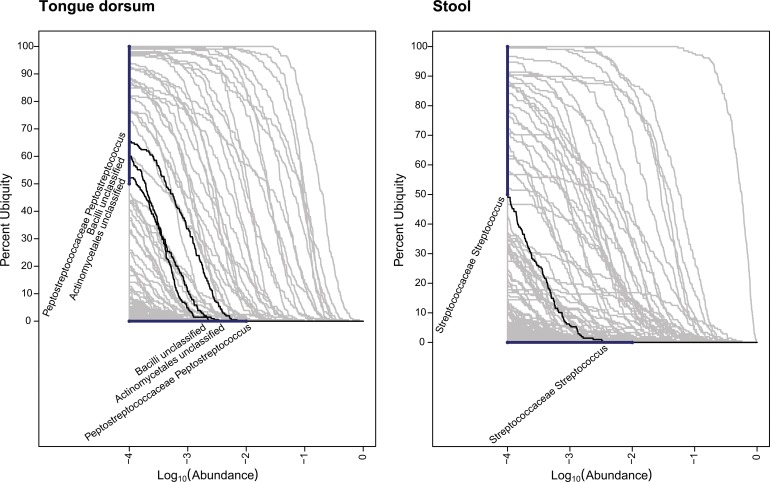
Genera Minor Core for 2 Body Habitats. In these Ub-Ab plots, heavy bars are drawn vertically on the y-axis from 50% to 100% ubiquity, and horizontally on the log_10_ transformed x-axis (abundance) from −4 to −2, representing an abundance of <1%. To qualify for greater than 50% ubiquity and less than 1% abundance, the Ub-Ab curve must originate from the range highlighted by the vertical bar and terminate on the range highlighted by the horizontal bar. The identified minor core taxa are identified for the tongue dorsum and stool body habitats.

The taxa identified within the majority of the cohort but at a low abundance are listed in Table 3 (Minor Core Taxa) and visualized for two body habitats in Figure 4 (Genera Minor Core for 2 Body Habitats). In Figure 4 (Genera Minor Core 2 Body Habitats), the tongue dorsum and stool habitats were highlighted to demonstrate how minor core taxa are identified in the two-parameter model with the Ub-Ab plots. In the tongue dorsum habitat, 3 minor core taxa were identified: *Peptostreptococcaceae Peptostreptoccocus*, *Bacilli* (Class), and *Actinomycetales* (Family). In the stool habitat, only one taxon was detected: *Streptococcaceae Streptococcus*. The body habitats of R antecubital fossa and vaginal introitus bore no minor core members, and thus were not illustrated. In all oral body regions, minor core members were detected, ranging from 1 to 4 members. With the exception of the anterior nares, with 1 minor core member, the remaining skin regions revealed no minor core members. Of the 3 vaginal regions, no minor core members were identified either.

**Table 3:** Minor Core Taxa.

Body Habitat	Number Identified	Taxa Identified	Maximum Ubiquity
**Oral**			
Buccal mucosa	4	*Coriobacteriaceae Atopobium*	54.23%
		*Prevotellaceae unclassified*	56.72%
		*Bacilli unclassified*	57.21%
		*Lachnospiraceae Catonella*	54.23%
Hard palate	2	*Clostridiales Family XIII. Mogibacterium*	73.87%
		*Lachnospiraceae Catonella*	89.95%
Keratinized gingiva	1	*Bacilli unclassified*	62.02%
Palatine Tonsils	2	*Clostridiales Family XIII. Mogibacterium*	75.36%
		*Firmicutes unclassified*	71.98%
Saliva	3	*Actinomycetales unclassified*	53.01%
		*Porphyromonadaceae Tannerella*	79.23%
		*Neisseriaceae Kingella*	60.11%
Subgingival plaque	1	*Firmicutes unclassified*	57.28%
Supragingival plaque	1	*Betaproteobacteria unclassified*	52.68%
Throat	2	*Clostridiales Family XIII. Mogibacterium*	74.75%
		*Firmicutes unclassified*	61.11%
Tongue dorsum	3	*Actinomycetales unclassified*	52.20%
		*Bacilli unclassified*	60.00%
		*Peptostreptococcaceae Peptostreptococcus*	65.37%
**Skin**			
Anterior nares	1	*Pseudomonadaceae Pseudomonas*	52.60%
L Antecubital fossa	0		
L Retroauricular crease	0		
R Antecubital fossa	0		
R Retroauricular crease	0		
**Vaginal**			
Mid vagina	0		
Posterior fornix	0		
Vaginal introitus	0		
**Stool**	1	*Streptococcaceae Streptococcus*	50.00%

These results confirm our prior diversity analyses [27] comparing median diversity versus pooled diversity, when measured with the low-abundance sensitive Tail diversity statistic. If the calculated median diversity across the donors is equal to the diversity measured when donors are pooled together, then the taxonomic diversity is common among the donors. However, if the pooled diversity is significantly greater than the median diversity, then even though the donors may appear to share the same measurement of richness and evenness, there are more taxa not shared among them. According to these prior pooled versus median diversity measurements, the oral body regions share more taxa, than the skin body regions. This is also reflected in the difference between the number of core and minor taxa between the oral and skin body sites. The more taxa that are shared among donors, the longer both the high abundance core and minor core list will be.

This set of low abundant taxa that are in the majority of the cohort support the notion that the long tail of the rank abundance curve should not only be considered composed of transient taxa occasionally acquired from the immediate environment. Instead, this minor core set of taxa may persistently contribute its own unique assemblage of genetic information to the habitat’s microbiome and as such represents a range of ecological strategies and sources of potentially both beneficial and pathogenic organisms.

### The Relative Variation of the Core Taxa

The variation in the abundance of a taxon across a cohort can be attributed to a combination of multiple evolutionary, ecological and stochastic sources. Prominent sources likely include dynamic biological relationships between the organism and its habitat, the interaction of predatory, competitive and cooperative strategies between community members, and of special consideration in the study of the human microbiome, influences from host genetics and lifestyle. If these influences are not uniform across the cohort, then these forces may result in the presence of multiple community structures that have recently been described as “biome types” within a body habitat, for example the description of “enterotypes” in the gut [28]. This implies that with a large enough cohort size, it is possible to detect subsets in the cohort that may exhibit community structures that are more similar to other members in the same subset than across other subsets. In analysis of variance (ANOVA) terms, the variance between subsets is statistically significantly greater than the variance within the subsets. The stability and degree of separation between biome types for a healthy adult human population remains to be further explored. Nonetheless, in this scenario, it would be possible to reduce the variance of taxa proportions across the cohort by stratification, for example, by a subset of taxa combinations or donor phenotype data. After stratification, the intra-strata variance would be more equivalent to that of a homogenous cohort. The final influence on variance is the abundance of the taxa. The closer the abundance approaches 0.5, the greater the variance, following the variance formula for a sample proportion [31]:
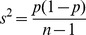
(1)


Here, *p* is the abundance and *n* is the number of reads per sample, assuming simple random sampling, i.e., no PCR amplification bias. This last source of variation is difficult to correct for because of non-constant reads per sample and un-measurable PCR amplification bias. To identify distinctively high or low variation taxa in a cohort, visualization is useful to identify patterns that may arise per dataset.

**Figure 5:**
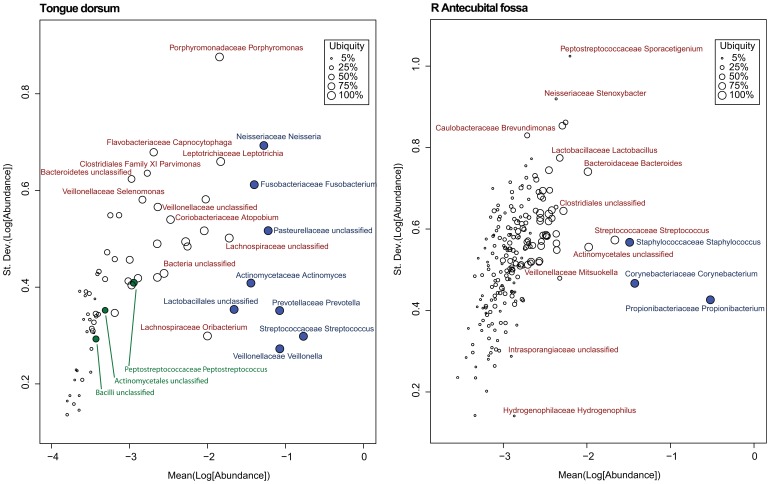
Variation of Abundance Plot. In these Var-Ab plots, the abundance is along the x-axis, and the variation is along the y-axis, with both measurements increasing towards the top right of the plot. Each glyph represents a single taxon, and its size is proportional to the ubiquity of the taxon, thus the larger the glyph, the more ubiquitous the taxon is across the cohort. Those taxa that were previously selected as core at 80% ubiquity and 1% abundance have been filled in with blue, and the minor core have been filled in with green. Taxa towards the top of the plot have greater variation than those on the bottom.

If a core set of taxa were to exist, not only would one expect that sufficient abundance and ubiquity are both requisites for the membership of a taxon, but also, across the cohort, its abundance would be more constant than non-core taxa. This constancy could be detected by a lower relative variation, if this characteristic was plotted against abundance. In Figure 5 (Variation of Abundance Plot), two variation versus abundance (Var-Ab) plots were selected for tongue dorsum and R antecubital fossa. See “Materials and Methods” for the steps and reasoning behind how the data was first transformed. When there is a clear core, such as in the R antecubital fossa, vaginal introitus or stool (not shown), then the core taxa will tend towards the bottom right of the variation plots, supporting the expectation that core taxa should be both stable, and thus exhibit low variation, and relatively high in abundance. The tongue dorsum body habitat is very distinctive among the body habitats sampled. As discussed earlier, selecting a single ubiquity-abundance pair was less clear than the other body habitats, and this is supported by the Ub-Ab plot for tongue dorsum in Figure 1 (Genera Ub-Ab Plots for 4 Body Habitats), where the non-core taxa curves interweaved with some of the core taxa curves. This complexity is also observed in the Var-Ab plot in Figure 5 (Variation of Abundance Plot). Instead of the core taxa being clustered only at the bottom right quadrant of the plot, the core, as identified with the two-parameter model, revealed *Fusobacterium* and *Neisseria*, as well. These taxa had among the highest variation across all taxa. The majority of Var-Ab plots resembled that of R antecubital fossa, sharing the characteristic that the core taxa were consistently restricted to the bottom right quadrant. The other distinctive characteristic of the tongue dorsum habitat’s Var-Ab plot are the locations of the 3 minor core taxa, which are identified in green. Due to the low abundance of the minor core, their variation tended to be low as well, but this may be a result of following the expected variance of estimated sample proportions (Equation 1). The Var-Ab plots for all 18 body habitats are available as Supplemental Material, File S2 (Var-Ab Plots 18 Body Habitats).

**Figure 6:**
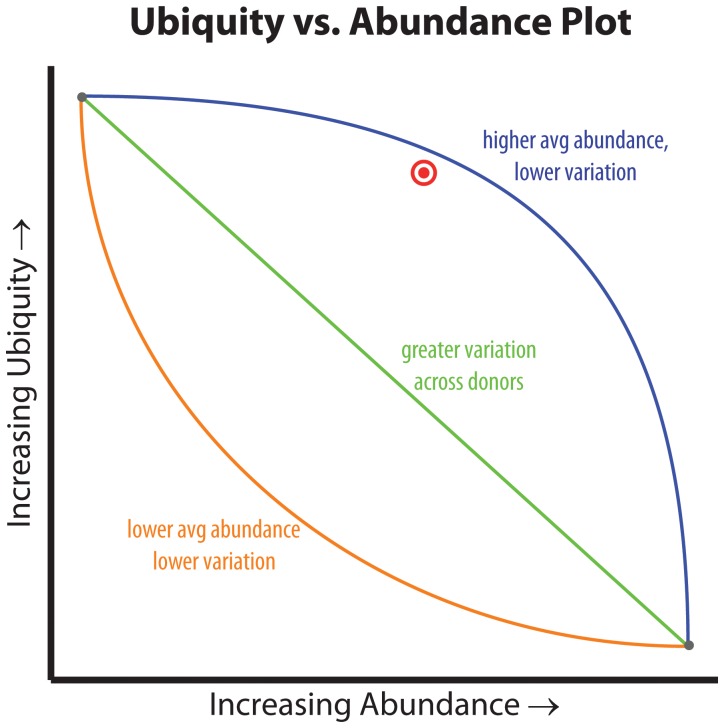
Inflection of the Ub-Ab Curve. The inflection of the Ub-Ab curve describes the distribution of a taxon’s abundance across the cohort. The blue curve (high abundance core) describes a taxon for a cohort with greater average abundance. Since the majority of the cohort had the taxon assayed at a large abundance, the variance will also be lower. The orange curve (minor core) describes a taxon across a cohort assayed with a majority of low abundant measurements. An average of these abundances will yield a smaller value, and because the taxon abundances are concentrated in the low abundance range, the variance will also be lower. The green line represents a taxa assayed with an even distribution of both high and low abundance, thus its variance will be greater than both taxa represented by the blue and orange curves.

The two-parameter model works well for identifying a core set of taxa, because the selection of the cutoffs also enforces the degree of inflection that the taxa curve must possess for inclusion in core membership. The greater the inflection, the more narrowly the abundance is observed across the members of the cohort. An example is illustrated in Figure 6 (Inflection of the Ub-Ab Curve), where the 2-parameter cutoff is able to differentiate between taxon curves with identical high ubiquity at low abundance and also low ubiquity at high abundance, but only with differences in variation and average abundance.

### The Core Taxa of the Body Regions

The core microbiome for each of the 4 body regions was computed using two different methods. This was necessary because each method answered a different question. One question that may be asked is, given a set of body habitats from a body region, “what is the probability that a body habitat from that body region will contain a taxon of interest at a specified abundance?” This may be useful in estimating which taxa may be discovered in a new habitat from an already characterized body region. This is calculated by averaging body habitat ubiquities across a body region. The alternative question that may be asked is, “what is the probability that a taxon of interest will be detected across all body habitats for a specified body region?” The answer to this question may relate more directly to the idea of a body region core, in the sense that the taxa that are identified must be detected in every body habitat for a region above the specified abundance cutoff. The methodology used to acquire the probability that a taxon will be discovered across all body habitats involves the “and”ing, or multiplying, of ubiquities across all body habitats of a body region. This implies that as more body habitats are included in a body region, fewer taxa will remain in the core, thus quickly reducing the core to low ubiquities, since repeated serial multiplication of values less than 1 can only reduce the final product’s magnitude. Thus, it may be less desirable to compare the core member sizes of multiple body regions, if the number of body habitats in each body region is not identical.

Due to the similarity of all body habitats within each body region, the averaged body region Ub-Ab plots have a very similar characteristic to those of the body habitats. Curves do appear to be smoother, but that is an artifact of reducing the quantization error in the ubiquity approximation when averaging is performed. As previously noted, body regions can be distinguished visually, however to compare body habitats from the same body region, a more sensitive and quantitative measure may need to be employed.

**Figure 7:**
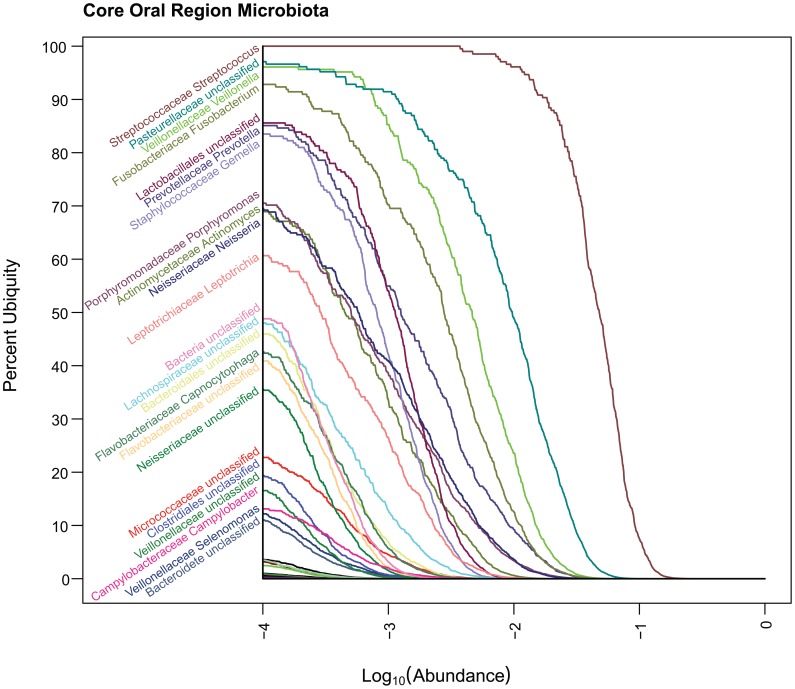
Core Oral Ub-Ab Plot. The Ub-Ab plot for the oral body region was constructed by multiplying the ubiquities together for each of the 9 oral habitats along all the abundances. At an abundance of 10^−4^, *Streptococcus*, *Pasteurellaceae* (Family), *Veillonella* and *Fusobacterium* were found in over 90% of the cohort.

**Figure 8:**
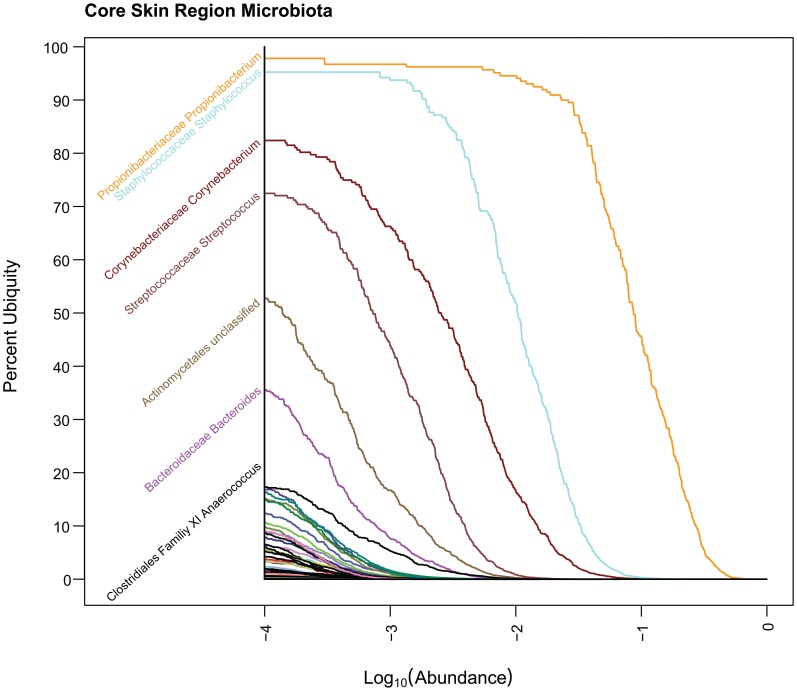
Core Skin Ub-Ab Plot. The Ub-Ab plot for the skin body region was constructed by multiplying the ubiquities together for each of the 5 skin habitats along all the abundances. At an abundance of 10^−4^, *Propionibacterium* and *Staphylococcus* was found in over 90% of the cohort.

**Figure 9:**
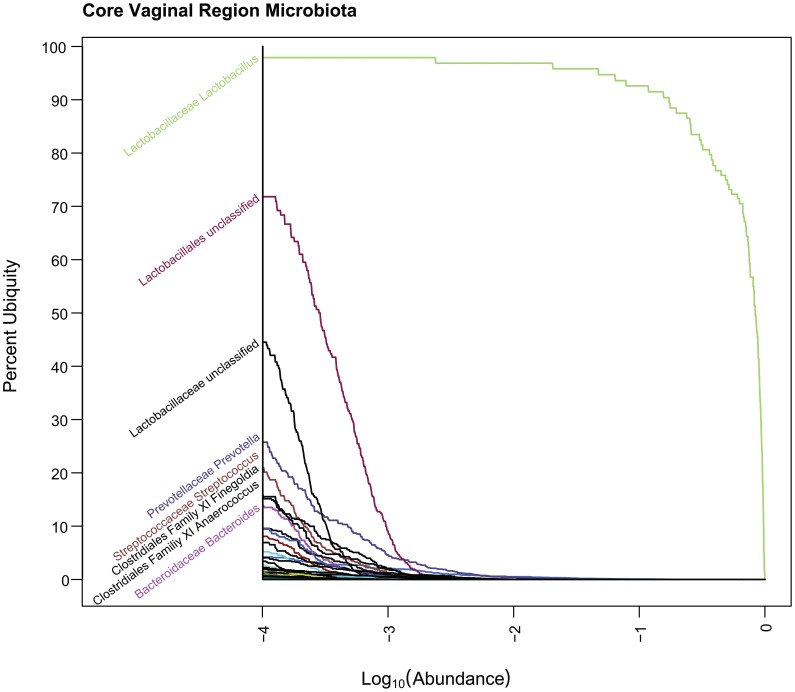
Core Vaginal Ub-Ab Plot. The Ub-Ab plot for the vaginal body region was constructed by multiplying the ubiquities together for each of the 3 vaginal habitats along all the abundances. At an abundance of 10^−4^, only *Lactobacillus* was found in over 90% of the cohort.

The plots for oral, skin and vagina for the “and”ing of body regions is provided in Figures 7, 8, and 9 (Core Ub-Ab Plots for oral, skin and vaginal regions, respectively). The plot for the stool body habitat was not provided, since it was the only body habitat in its body region. The differences between the core microbiota at body regions versus those in the body habitats are very apparent because the number of taxa identifiable as core is reduced significantly. Due to this reduction of core, for this discussion, a cutoff of >80% ubiquity and a looser cutoff of >.01% (i.e., 10^−4^) abundance was applied. In the oral body region, the core consisted of 7 taxa, 2 of which could not be confidently classified to the genus level. In the order of decreasing ubiquity, these were: *Streptococcus*, *Pasteurellacaceae* (family), *Veillonella*, *Fusobacterium*, *Lactobacillales* (family), *Prevotella*, and *Gemella*. The skin body region’s core was composed of 3 genera: *Propionibacterium*, *Staphylococcus*, and *Corynebacterium*. In the vaginal body region, the only taxon above the cutoff was *Lactobacillus*. From these body region plots, it is possible to identify even the low abundant members of the core microbiome members for all 3 body regions.

### Comparing the Microbiota of Two Cohorts

A methodology for comparing the micobiota of two cohorts is crucial for the analysis of experiments involving control and treatment groups. Each member of a cohort is actually a sample from the population of interest, thus making the cohort size the actual sample size that should be used in testing for the statistical significance of differences between the microbiota of any two groups. Utilizing the number of sequenced reads per sample as the sample size will generate incorrect conclusions with respect to the statistical significance of any differences identified. The high sequencing depths made available from 454 pyrosequencing should be defined as “amplification magnitude”, rather than “sampling depth”. In the early cycles of PCR, 16S sequences are “sampled” from the bacterial genomes present as the template and then amplification occurs exponentially. Subsequent PCR cycles may preferentially amplify the initial copies over the original templates, thus leading to the magnification of early sampling bias. As a result, pairwise analyses of 16S sample replicates with moderate sequencing depth frequently reject the null hypothesis of no difference when using a statistical methodology such as Pearson’s χ^2^ test. As a result, pooling samples, focusing on sequencing depth, while not taking the actual cohort sizes into account, detrimentally hides critical information that could be useful and may generate misleading inferences. The advantage of utilizing the proposed two-parameter model for the comparison of two cohorts is the attempt to circumvent these issues by allowing the sampling variance of each cohort to be reflected in the taxonomic ubiquity that is compared.

Recall, the Ub-Ab plot represents the relationship between abundance and ubiquity for all taxa across all the donors in a specific cohort. In the HMP datasets that have been studied, the cohort of donors passed a screening for systemic health, implying that the taxa represented and their characteristic Ub-Ab curves should be preserved for another random set of healthy individuals, if the same criteria were used for inclusion and exclusion. A comparison of the Ub-Ab plots between two cohorts should reveal differences in the core microbiomes, such as control and treatment. To estimate the amount of deviation that would represent a significant difference between the microbiota of two cohorts, it is necessary to first quantify the variation in a cohort by measuring the statistic’s variance within the cohort (i.e., estimate a null distribution), and then calculate a p-value to give confidence that the statistic’s magnitude is significant.

First, to visualize the differences between the microbiomes of two cohorts, a plot based on the Ub-Ab plot is introduced. The Ubiquity-Ubiquity (U-U) plot provides a visualization of the differences between microbiomes by incorporating the ubiquity measurements of two Ub-Ab plots into a single plot. The x and y axes of the U-U plot are ubiquities, thus their minimum and maximum percentages range from 0 to 100. For each taxon, a curve is drawn that represents a shift of the ubiquity between the two cohorts. If the ubiquities of a taxon are identical across all abundances between the two compared cohorts, then a line will fall along the diagonal, where the thick grey line has been drawn as a “no change” reference. The ubiquities for the first and second cohort are represented along the x-axis and y-axis, respectively. The magnitude of the positive shift of a taxon’s abundance in the second cohort is represented by the distance of the taxon’s curve’s deviation above the grey reference line. Contrariwise, the magnitude of the negative shift of a taxon’s abundance in the second cohort is represented by the deviation of the taxon’s curve below the grey reference line. To quantify the magnitude of the differences between cohorts captured by a U-U plot, an abundance-weighted KS (AWKS) statistic (See "Material and Methods") was computed between the compared samples.

#### Comparing the microbiome of similar body habitats

**Figure 10:**
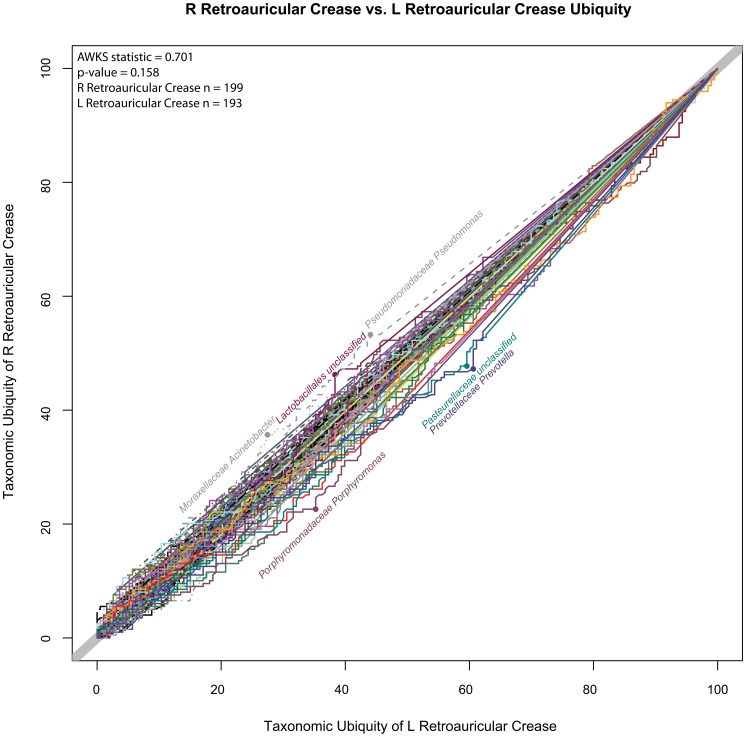
L vs. R Retroauricular Crease U-U Plot. This U-U plot represents the taxonomic differences between the L and R retroauricular crease samples collected from the cohort. The taxonomic ubiquity of the L and R retroauricular crease is represented along the x-axis and y-axis, respectively. If a U-U curve falls along the diagonal line with a slope of 1, then there is no difference between the ubiquities across the abundances for that taxon. The more significant the differences between the ubiquities of a taxon, the further off the diagonal its U-U curve will deviate. The most saliently deviating U-U curves are labeled. The p-value (0.158) for the AWKS statistic computed between the symmetric body habits indicate that there was not a statistically significant (α = 0.05) difference between the microbial communities of the two symmetric body habitats.

A comparison of the L and R retroauricular crease was performed to demonstrate the magnitude of variation that could be expected and observed if sampling replicates were analyzed from the same body habitat. This pair was chosen because the L and R retroauricular crease samples could almost be considered sampling replicates, since detected variation would not be as confounded by time, collector, or sample processing (Figure 10, L vs R Retroauricular Crease U-U Plot). In this U-U plot, the reader may observe that there was not a large shift of any particular genus between the two habitats. A few of the genera exhibiting the greatest shifts are labeled, however overall, the AWKS statistic measuring the difference between the microbiomes of the cohorts was not significant (p-value = 0.158, α = 0.05). This example demonstrates how similar microbiomes and those genera which specifically contributed to the most extreme changes in ubiquity between two cohorts can be quickly identified.

#### Comparing the microbiota of dissimilar body habitats

**Figure 11:**
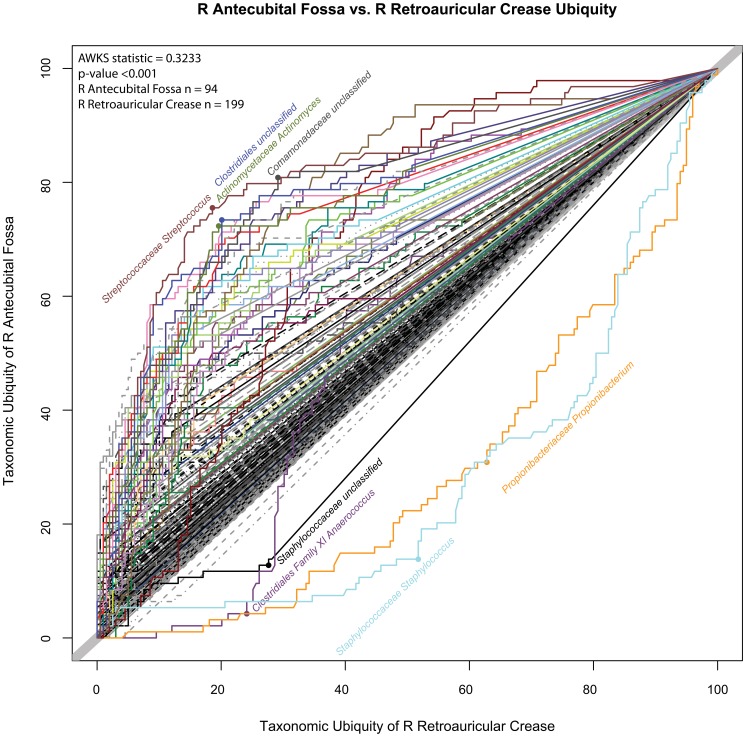
R Retroauricular Crease vs.R Antecubital Fossa U-U Plot. This U-U plot represents the taxonomic differences between the two skin body habitats of R retroauricular crease and R antecubital fossa. The ubiquity of taxa for R retroauricular crease and R antecubital fossa are represented along the x-axis and y-axis, respectively. Taxonomic shifts toward the R antecubital fossa are represented by curves above the diagonal reference line. This plot reveals the greater taxonomic presence of *Staphylococcus*, *Propionibacterium*, *Anaerococcus*, and *Staphylococcaceae* (family) in the R retroauricular crease. The labeled points represent where the greatest difference of ubiquities are found. For example, at matching abundances, the maximum difference in ubiquity for *Streptococcus* was found at 76% of the cohort in R antecubital fossa, but only at 19% of the cohort in R retroauricular crease.

To demonstrate the sensitivity of the U-U plot in detecting changes of the microbiota between two body habitats from the same skin body region, a comparison of the R antecubital fossa was made against the R retroauricular crease (Figure 11, R Retroauricular Crease vs R Antecubital Fossa U-U Plot).

In contrast to the left versus right comparisons of the retroauricular crease body habitats, when these two different body habitats were compared, a relatively greater significant difference of the microbiota could be discerned. Considering the R retroauricular crease, which is represented by the curves below the diagonal, the genera of *Staphylococcus*, *Propionibacterium* and *Anaerococcus* were more ubiquitous. For *Staphylococcus*, the greatest difference in ubiquity occurred at 52% in R retroauricular crease, versus 14% in the R antecubital fossa. The underlying core OTU for *Staphylococcus*, #5, had a maximum shift from 52% to 15% (not shown). Since the measured abundances are analyzed as compositional data, the decrease in relative abundance for these genera in R antecubital fossa, necessarily increased the proportions for its taxa in the R retroauricular crease. The greater diversity of the microbiota in the R antecubital fossa allowed the increase of ubiquity to be distributed across a larger number of taxa. One of the most salient differences of ubiquity could be found for *Streptococcus* at 19% and 76%, for R retroauricular crease and R antecubital fossa, respectively. The underlying core OTUs (not shown) for *Streptococcus* had similar shifts in ubiquity for OTU #2 from 22% to 77%, and for #6 from 23% to 78%. The computed p-value for the difference between the two body habitats using their genera-based profiles was <0.001 with an AWKS statistic of 0.3233.

#### Comparing the microbiota between 2 visits

**Figure 12:**
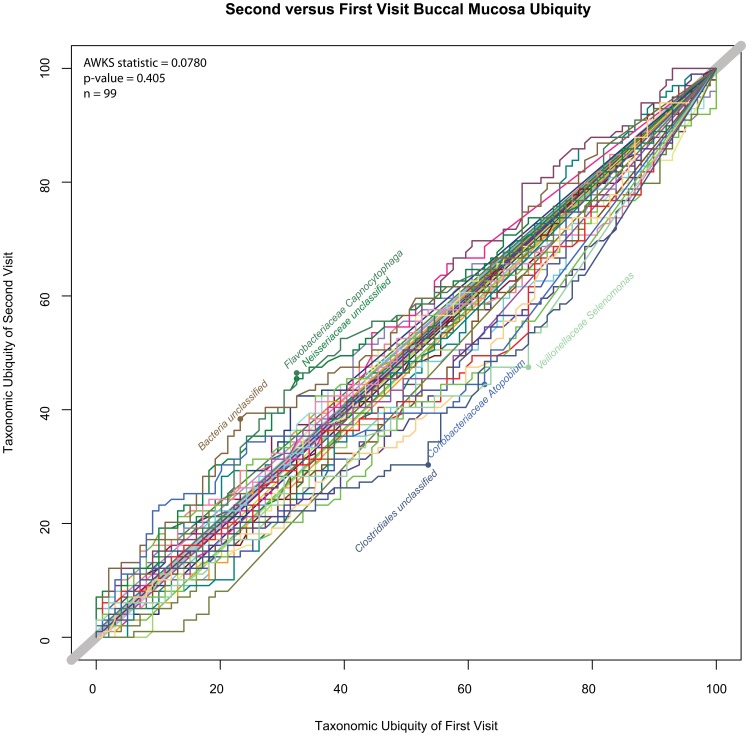
Buccal Mucosa, Second vs. First Visit U-U Plot. This U-U plot represents the comparison between the first and second visits for the buccal mucosa body habitat. There was not a statistically significant different between the two visits (p-value = 0.405, α = 0.05).

**Figure 13:**
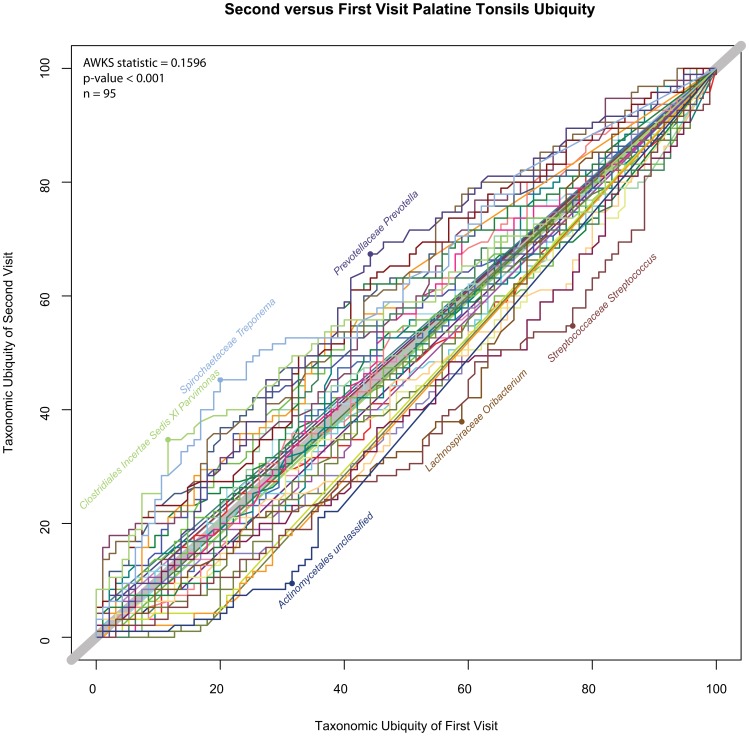
Palatine Tonsils, Second vs.First Visit U-U Plot. This U-U plot represents the comparison between the first and second visits for the palatine tonsils body habitat. There was a statistically significant difference between the two visits (p-value <0.001, α = 0.05).

A subset of individuals from the first study was resampled at a later time. In order to determine if there had been any significant shift of the cohort’s microbiota over time, the AWKS statistic and p-values were computed between first and second visits for all body habitats. The calculations were performed on profiles based on both genera and OTUs. See Table 4 (Comparisons of First and Second Visit for 18 Body Habitats). Using a false discovery rate (FDR) rate of α = 0.05, of the 18 body habitats that were tested, 10 were rejected using genera-based profiles and 9 were rejected using OTU-based profiles, where the null hypothesis was that there was no differences between visits. While the order of statistical significance was slightly different between OTU and genera-based profiles, the only conflict in the set of body habitats rejecting the null hypothesis (“no difference between visits”) was the saliva habitat. The “1-Tailed p-value” column contains the unadjusted p-value between the first and second visit, and the “BH Adjusted α Threshold” column contains the Benjamini and Hochberg [29] adjusted threshold for a FDR with α = 0.05, m = 18. A body habitat was considered statistically significantly different, if the 1-tailed p-value was less than the BH adjusted α threshold. Slightly more than half of skin and oral samples appeared to be statistically significantly different between visits. The single stool habitat was statistically significantly different between visits, as well. In contrast, the vaginal body habitats tended not to show statistical significance between the donors’ two visits. Figure 12 (Buccal Mucosa, Second vs. First Visit U-U Plot) and Figure 13 (Palatine Tonsils, Second vs. First Visit U-U Plot) are included to visually demonstrate the differences between a body habitat with an insignificant change versus one with a significant change. From this analysis, it appears that there is not a clear distinction between microbiomes of body habitats that do or do not change between visits for skin and oral body regions, since a small change in the targeted FDR rate would change the number of body habitats that rejected the null hypothesis. Only the vaginal body habitats appeared to be stable between visits, as both skin and oral body habitats fell along a spectrum. It is important to note that because the profiles represent a collection of healthy individuals, rather than replicates of the same individual, changes in an individual’s microbiome over time may be more statistically significant than the results would indicate for a cohort over time. For example, if biome types were to exist in a cohort and individuals shifted biome types as if they were discrete states, a methodology based on comparing heterogeneous cohorts would not detect individual shifts, if the biome type proportions remained constant across the cohort. Additional research associating the range of disease severity and the magnitude of dysbiosis would aid in determining what limits must be crossed in order to confidently associate a microbiome state with a diseased individual based on the comparison with a healthy cohort alone. Additional work, for example, following a cohort of healthy donors and producing a time series data set would better quantify the amount of variation that could be considered healthy for that duration for a single individual, thus better defining biological significance, rather than the statistical significance that is currently being quantified.

**Table 4:** Comparisons of First and Second Visit for 18 Body Habitats.

Genera							
	Body Region	Body Habitat	Awks Statistic	1-Tailed p-value	Number of Donors	BH Adjusted α Threshold (FWE α = 0.05)	Null Hypothesis (No difference between visits)
1	Oral	Palatine Tonsils	0.160	<0.001	95	0.003	Reject
2	Oral	Subgingival plaque	0.147	<0.001	98	0.006	Reject
3	Oral	Supragingival plaque	0.133	<0.001	95	0.008	Reject
4	Oral	Throat	0.140	0.001	93	0.011	Reject
5	Skin	L Antecubital fossa	0.334	0.002	14	0.014	Reject
6	Oral	Keratinized gingiva	0.138	0.005	88	0.017	Reject
7	Stool	Stool	0.127	0.005	104	0.019	Reject
8	Oral	Saliva*	0.114	0.016	83	0.022	Reject
9	Skin	R Antecubital fossa	0.291	0.023	13	0.025	Reject
10	Skin	L Retroauricular crease	0.154	0.024	66	0.028	Reject
11	Skin	Anterior nares	0.128	0.033	62	0.031	Accept
12	Oral	Tongue dorsum	0.092	0.120	99	0.033	Accept
13	Skin	R Retroauricular crease	0.109	0.152	75	0.036	Accept
14	Oral	Hard palate	0.095	0.186	86	0.039	Accept
15	Vaginal	Vaginal introitus	0.129	0.247	38	0.042	Accept
16	Oral	Buccal mucosa	0.078	0.405	99	0.044	Accept
17	Vaginal	Mid vagina	0.099	0.471	40	0.047	Accept
18	Vaginal	Posterior fornix	0.077	0.603	40	0.050	Accept

OTUs							
	Body Region	Body Habitat	Awks Statistic	1-Tailed p-value	Number of Donors	BH Adjusted α Threshold (FWE α = 0.05)	Null Hypothesis (No difference between visits)
1	Oral	Subgingival plaque	0.136	<0.001	98	0.001	Reject
2	Oral	Palatine Tonsils	0.132	<0.001	95	0.006	Reject
3	Stool	Stool	0.112	<0.001	104	0.008	Reject
4	Skin	L Antecubital fossa	0.321	0.001	14	0.011	Reject
5	Oral	Keratinized gingiva	0.128	0.004	88	0.014	Reject
6	Oral	Throat	0.115	0.004	93	0.017	Reject
7	Oral	Supragingival plaque	0.114	0.005	95	0.019	Reject
8	Skin	R Antecubital fossa	0.284	0.006	13	0.022	Reject
9	Skin	L Retroauricular crease	0.150	0.013	66	0.025	Reject
10	Oral	Saliva*	0.099	0.058	83	0.028	Accept
11	Oral	Hard palate	0.101	0.088	86	0.031	Accept
12	Skin	Anterior nares	0.111	0.103	62	0.033	Accept
13	Oral	Tongue dorsum	0.090	0.125	99	0.036	Accept
14	Skin	R Retroauricular crease	0.106	0.163	75	0.039	Accept
15	Oral	Buccal mucosa	0.085	0.238	99	0.042	Accept
16	Vaginal	Mid vagina	0.120	0.260	40	0.044	Accept
17	Vaginal	Vaginal introitus	0.106	0.470	38	0.047	Accept
18	Vaginal	Posterior fornix	0.053	0.853	40	0.050	Accept

* With the exception of saliva, genera and OTU-based analyses agree on which body habitats exhibit no significant differences between visits.

## Conclusions

A systematic and quantitative approach towards the identification of the core microbiomes of body habitats across the large cohort of donors made available through the HMP revealed distinct patterns in the relationship between ubiquity and abundance among the 4 body regions. The Ub-Ab plots identify which organisms contribute to the taxonomic diversity within a cohort, exposing low abundant taxa with high ubiquity, while simultaneously providing a backdrop of other taxa as context for their importance. A significant number of low abundant taxa, while not ubiquitous at a high degree, i.e., >80%, were discovered across the cohort’s body habitats, revealing the vast richness that may contribute to the microbial community’s sources of genetic material (whether as a pool available for generating mutations or lateral gene exchange), biological functions, regulatory processes, or other interactions within the community ecology. While a single ubiquity and abundance cutoff may be specified to define the core members of a microbiome rigorously, a single parameter pair does not appear to be generalizable for all body habitats, as there is a continuum of possible definitions from which one single parameter pair cannot be preordained. From an alternate perspective of variation and its relationship with abundance, the two-parameter definition of a core is supported in terms of a taxa’s stability across the cohort, providing further support for the potential importance of a contingent of minor core. While there may be statistically significant differences in the microbiome for many body habitats over time, a general microbiome signature appears to be reasonably conserved among body habitats of the same body region and between visits suggesting that a relatively stable microbial community structure is maintained throughout the human microbiota, possibly serving as an overall indicator of health. Analyses of the variation for the abundances of taxa across the cohort reveal that the detected core taxa are also stable. An examination of the OTUs comprising each genera-based taxonomic unit indicates that there were no body habitat specific core OTUs and that the same OTUs were conserved across the cohort as well.

A methodology for visually comparing the microbiota of two cohorts was introduced. With these U-U plots, it was visually demonstrated by comparing two similar and two dissimilar body habits, the expected background variation and key taxa that contribute to the differences between the body habitats, respectively. The abundance weighted KS statistic was then introduced as a means to quantify the differences seen in the U-U plots, and applied to the 18 body habitats to determine how constant the microbial community structure is conserved between the first and second visits that were collected from the healthy cohort. Statistical inference was performed with the aid of bootstrapping. This led to the observation that oral and stool habitats tended to vary more than skin and vaginal body regions. The lack of a discrete separation between conserved and varying microbiomes of habitats between visits revealed that much like the complexity of defining the core taxonomic members of a microbiome across a healthy cohort, addressing the question of whether a microbiome varies over time, also falls along a continuous spectrum.

## Materials and Methods

### Ethics Statement

As a part of a multi-institutional collaboration, the Human Microbiome Project human subjects study was reviewed by the Institutional Review Boards at Baylor College of Medicine under IRB Protocol H-22895, the Washington University School of Medicine under protocol number HMP-07-001 (IRB ID# 201105198) and at the J. Craig Venter Institute under IRB Protocol Number 2008-084. All study participants gave their written informed consent before sampling and the study was conducted using the Human Microbiome Project Core Sampling Protocol A. Each IRB has a federal wide assurance and follows the regulations established at 45 CFR Part 46. The study was conducted in accordance with the ethical principles expressed in the Declaration of Helsinki and the requirements of applicable federal regulations.

### Utilizing the Binomial Distribution to Qualify Presence and Absence

To properly qualify if a taxon of interest should be considered in or out of a set of taxa, it is not sufficient to use a “fixed percentage abundance” cutoff or a “greater than zero” approach. Neither of these approaches takes into account the sampling depth, i.e. reads per sample. Awareness of the sampling depth is crucial for comparing two samples with low abundance taxa, especially when these two samples have been sampled at different depths and have a significant proportion of taxa with abundances barely at, or just below, the maximum sampling sensitivity. The inverse of the sampling depth is the lower bound for the least abundant taxa that may be quantified. To properly determine whether a taxon should be in or out of a set, it is useful to make this Boolean decision based on a probabilistic condition, or cutoff. To include a taxon of interest into a set, an assertion of the confidence of resampling redetection should be made. One possible assertion is that upon repeated resampling and resequencing, the taxon of interest will be detected (i.e., have an abundance greater than zero) in at least 95% of the samples. This calculation can be made using the binomial distribution. The binomial distribution’s probability mass function is described by two parameters, *n*, number of trials (read depth) and *p*, the probability of successful detection (abundance).
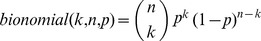
(2)


At k = 0, the function can be simplified to return the probability of no taxa detection, given the specified *n* and *p*.
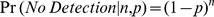
(3)


If this probability of no detection is greater than 0.05, there is a less than a 95% chance that the taxa will be detected upon resampling, and thus should not be considered present in downstream analyses.

### Computing Ubiquity vs. Abundance (Ub-Ab) Plots

The Ub-Ab plots demonstrate, for each taxon, the diminishing proportion of the samples that contain the taxon of interest, as the abundance cutoff increases. As one would expect, at a low abundance cutoff, the probability of a sample containing a taxon exceeding that cutoff, is greater. The definition of the ubiquity at abundance *a*, for cohort *X*, is specified for each taxon *t*, and is defined as:
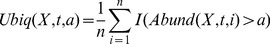
(4)

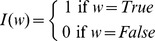



The values of ubiquity are proportions ranging from 0 to 1, because the number of samples containing the taxon of interest *t*, exceeding the abundance cutoff *a*, is normalized by the total number of samples *n.* I(w) is an indicator function that takes on the value of 1 if *w* is true, or 0 if *w* is false. *Abund*(*X*, *t*, *i*) is the proportion of taxon *t* in sample *i* in cohort *X*. The reader familiar with probability theory will notice that the ubiquity would be a cumulative distribution function, if the “greater than” sign were reversed.

Since a large proportion of taxa are low in abundance, prior to plotting the relationship between ubiquity and abundance, it is useful to first log_10_ transform the abundances. Thus, a ubiquity vs. abundance plot would have a domain of log_10_(min(*Abund*(*X*, *t*, *i*))) to 0, and a range of 0% to 100%.

### Computing Taxonomic Variance across the Cohort

The definition of the core taxonomic members of a microbiome presented in this study used a cutoff for abundance and ubiquity to elect candidates for inclusion. Across the cohort, even if a taxon is ubiquitous, the variation of that abundance in each donor will vary as well. This may reflect a variety of conditions such as the physiochemical range in which the taxon in question is adapted to, and/or the cooperative or competitive lifestyle interactions with the other members of the microbial community also inhabiting the body habitat in question. In order to measure the variation of the abundance across the cohort, three key issues needed to be addressed. The first issue is the non-normal distribution of abundances. Taxonomic distributions tend to follow a power-law probability distribution. Thus, a log_10_ transformation was applied to the abundance before computing the variance. This introduced the second issue of zero counts in the data. Zeroes may occur in the data due to the read sampling depth being insufficient to assay a very low abundance, thus leading to transformation errors resulting from log_10_(0) equaling negative infinity. To deal with this problem, it is common to treat a zero abundance as a “non-response” [30], thereby excluding it from the analysis and reducing the sample size for that taxa. Due to the various read depths used, a minimum abundance of 10^−4^ was used as a lower bound for presence. The final issue was the correlation between variance and proportion. The variance for a sample proportion [31] is estimated by the formula, p(1-p)/(n-1). As *p* (the abundance) approaches 0 or 1 from the midpoint proportion of 0.5, or *n* (the reads per sample) increases, the sampling variance will decline independent of the taxon’s biological characteristics. Since reads per sample varied and amplification bias may occur, applying a correction to the observed variance based on a theoretical expected variance, does not work well. Thus, a scatter plot was used to better identify the taxa that do not follow a common trend. In these plots, the x-axis is the mean of the log transformed proportions, and the y-axis is the standard deviation of the log transformed abundance. Utilizing the standard deviation in the plot, instead of the variance, makes the scale more intuitive and relative to the log abundance. To account for the variability of sample size, i.e. ubiquity, for each taxon, each glyph’s size was plotted proportional in size to the ubiquity of the underlying taxon. From these plots, the core taxa tended to be located in the bottom right quadrant, because of their characterization by low variance and high abundance. The minor core, if existent in a body habitat, may be found at comparable levels of variation to the high abundance core, but located in the bottom left quadrant, where they are characterized by their low abundance and low variance, but wide ubiquity.

### Computing Ubiquity-Ubiquity Plots (U-U Plots)

A comparison of two groups of samples based on each taxon’s relationship of abundance and ubiquity can be performed by matching the abundances between the two groups. The ubiquity comparison plot (U-U Plot) represents one group of samples (1^st^ cohort) on the x-axis, and the second group of samples (2^nd^ cohort) on the y-axis. The units on both axes represent ubiquity, and thus range from 0% to 100%. When the taxonomic abundance of the two samples compared have the same ubiquity, they will fall on the idealized diagonal line of slope = 1 and y-intercept = 0. Thus, the more similar the microbial communities of two groups are the closer to this line they will fall. Deviations of the U-U plotted lines above the idealized diagonal indicate a greater ubiquity towards the group labeled on the y-axis, while curves deviating below the idealized diagonal represent greater ubiquity towards the group represented along the x-axis. All U-U curves will diverge from (0, 0) and then converge at (100%, 100%). The reader familiar with statistical methods may recognize the similarity of the U-U plot to the quantile-quantile (Q-Q plot) [32] or Receiver Operator Characteristic (ROC) curve [22]. Both are commonly used for comparing two cumulative distribution functions.

### Statistical Inference on the U-U Plots

The U-U plot provides a visual representation of the difference between the microbiomes of two cohorts. However, in order to determine whether the differences visualized are statistically significant, it is necessary to quantify both the potential variation in the sampled donors and the variation due to 16S amplification by computing the null distribution through bootstrapping. Thus, the null hypothesis (H_0_) would then be that the two cohorts have the same taxonomic profile, and the alternate hypothesis (H_A_) would be that there existed a difference. Standard caveats hold that 1) statistical significance is not the same as biological or practical significance, where an effect size can be associated with a disease state, 2) with large sample sizes, methods can be very sensitive to small deviations, and 3) the act of sampling without replacement will perturb the system. To measure the difference between taxonomic profiles of two cohorts, a test statistic was devised in order to capture the difference of ubiquities between cohorts across abundances, for each taxon. This test statistic is computed between the compositional representations of the two cohorts, and will be referred to as the Abundance-Weighted Kolmogorov-Smirnov (AWKS) statistic:
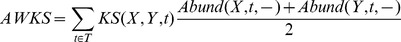
(5)


AWKS is the abundance weighted average of the Kolmogorov-Smirnov (KS) statistics measured for every taxon between the cohorts, X and Y. *T* is the union of taxa between cohorts X and Y. *Abund(X,t,-)* and *Abund(Y,t,-)* are the average abundance of taxa *t*, across all the individuals in cohort *X* and *Y*, respectively.
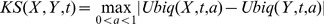
(6)


The Kolmogorov-Smirnov (KS) statistic between two cohorts *X* and *Y* is calculated for a single taxon as the maximum absolute difference between the ubiquities at matching abundance levels.
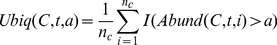
(7)


The *Ubiq(C, t, a)* is a cumulative distribution function defined as the proportion of donors in cohort *C*, for taxa *t*, above the abundance cutoff *a*. *I* is the indicator function, which returns 1 if the argument is true, otherwise 0, if the argument is false. *n_c_* is the size of cohort *C*.

The null distribution for the AWKS statistic was computed for 1000 bootstrap replicates by resampling with replacement both the donors’ samples and each sample’s read counts for each body habitat. The test for normality, using the Shapiro-Wilks test, rejected the null hypothesis that the bootstrapped AWKS null distribution was from a Gaussian distribution. Thus, each generated AWKS null distribution was utilized directly to calculate the p-values for each of the AWKS statistic based inferences.

### Software Availability

The R and Perl scripts used to generate the Ub-Ab, U-U, and Var-Ab plots, as well as computing core CIs are available through Sourceforge. The project is named Corbata (CORe microBiome Analysis Tools). The direct project URL is:

http://sourceforge.net/projects/corbata/.

Additional information is also available at:

http://www.jcvi.org/cms/research/projects/corbata.

### 16S rRNA Sequence Data Processing and Data Set

Sequence reads were processed using a pipeline constructed by the HMP Consortium as described in [13]. Data from the “high stringency” pipeline was used in this analysis and quality was assured with the following steps described here in brief. For sample multiplex barcode deconvolution and 16S primer trimming, a one nucleotide unambiguous mismatch to the sample barcode and up to two nucleotide mismatches to the adjacent PCR primer were allowed, respectively. Sequences with an ambiguous base call or a homopolymer stretch longer than eight nucleotides were removed from subsequent analyses. The high stringency pipeline incorporated a strategy of calculating the average quality score within a 50 nucleotide window that was shifted along the sequence. When the average quality score of the window decreased to <35, the sequence was trimmed. After trimming, all sequences were aligned using a NAST-based sequence aligner [33] to a custom reference based on the SILVA [34] database of curated alignments. Sequences shorter than 200 nucleotides or that did not align to the anticipated region of the reference alignment were removed and extraneous bases that extended beyond the targeted variable region were trimmed. Chimeric sequences were then identified using the Mothur [26] implementation of the ChimeraSlayer [35] algorithm which was trained to the “Gold” database (http://microbiomeutil.sourceforge.net). These filtered alignments formed the data set used for the generation of taxonomic classifications using RDP [36]. From the read processing described above, a total of 3,044 samples (17,371,356 total reads) were analysed.

## Supporting Information

File S1 **Ub-Ab Plots 18 Body Habitats.** This PDF file contains the Ub-Ab plots for all 18 HMP body habitats under study. Colors were only assigned to the 50 most abundant taxa. Abundance for each taxon was computed by averaging across the entire cohort. See manuscript for additional details.
(PDF)

File S2 **Var-Ab Plots 18 Body Habitats.** This PDF file contains the Var-Ab plots for all 18 HMP body habitats under study. Not all taxa were labeled in order to reduce clutter. See manuscript for additional details.
(PDF)
