# Supplementary material for: Analyses of the Stability and Core Taxonomic Memberships of the Human Microbiome
Source: PLoS One. 2013 May 6;8(5):e63139. doi: 10.1371/journal.pone.0063139 (PMC3646044; doi:10.1371/journal.pone.0063139)
Supplement: File S1 — Ub-Ab Plots 18 Body Habitats. This PDF file contains the Ub-Ab plots for all 18 HMP body habitats under study. Colors were only assigned to the 50 most abundant taxa. Abundance for each taxon was computed by averaging across the entire cohort. See manuscript for additional details. (PDF) [file pone.0063139.s001.pdf]

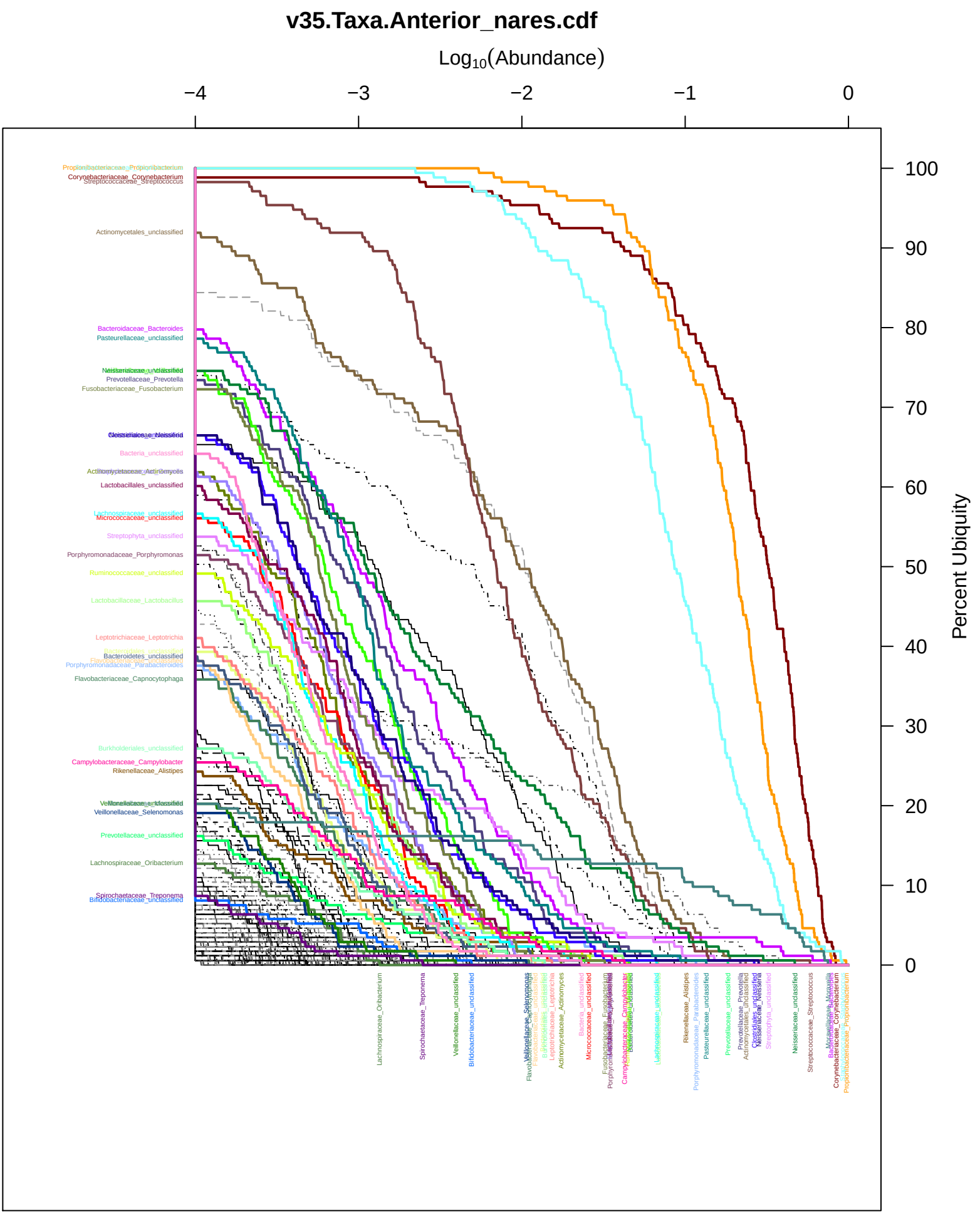
v35.Taxa.Anterior_nares.cdf
Log10(Abundance)
−4
−3
−2
−1
0
Percent Ubiquity
100
90
80
70
60
50
40
30
20
10
0
Propionibacteriaceae_Propionibacterium
Corynebacteriaceae_Corynebacterium
Streptococcaceae_Streptococcus
Actinomycetales_unclassified
Bacteroidaceae_Bacteroides
Pasteurellaceae_unclassified
Neisseriaceae_unclassified
Prevotellaceae_Prevotella
Fusobacteriaceae_Fusobacterium
Neisseriaceae_Neisseria
Bacteria_unclassified
Actinomycetaceae_Actinomyces
Lactobacillales_unclassified
Lachnospiraceae_unclassified
Micrococcaceae_unclassified
Streptophyta_unclassified
Porphyromonadaceae_Porphyromonas
Ruminococcaceae_unclassified
Lactobacillaceae_Lactobacillus
Leptotrichiaceae_Leptotrichia
Bacteroidetes_unclassified
Porphyromonadaceae_Parabacteroides
Flavobacteriaceae_Capnocytophaga
Burkholderiales_unclassified
Campylobacteraceae_Campylobacter
Rikenellaceae_Alistipes
Veillonellaceae_unclassified
Veillonellaceae_Selenomonas
Prevotellaceae_unclassified
Lachnospiraceae_Oribacterium
Spirochaetaceae_Treponema
Bifidobacteriaceae_unclassified
Lachnospiraceae_Oribacterium
Spirochaetaceae_Treponema
Veillonellaceae_unclassified
Bifidobacteriaceae_unclassified
Veillonellaceae_Selenomonas
Flavobacteriaceae_unclassified
Burkholderiales_unclassified
Leptotrichiaceae_Leptotrichia
Actinomycetaceae_Actinomyces
Bacteria_unclassified
Micrococcaceae_unclassified
Fusobacteriaceae_Fusobacterium
Porphyromonadaceae_Porphyromonas
Bacteroidetes_unclassified
Campylobacteraceae_Campylobacter
Actinomycetales_unclassified
Lachnospiraceae_unclassified
Rikenellaceae_Alistipes
Porphyromonadaceae_Parabacteroides
Pasteurellaceae_unclassified
Prevotellaceae_unclassified
Prevotellaceae_Prevotella
Actinomycetales_unclassified
Clostridiales_unclassified
Neisseriaceae_Neisseria
Streptophyta_unclassified
Neisseriaceae_unclassified
Streptococcaceae_Streptococcus
Moraxellaceae_Moraxella
Corynebacteriaceae_Corynebacterium
Propionibacteriaceae_Propionibacterium

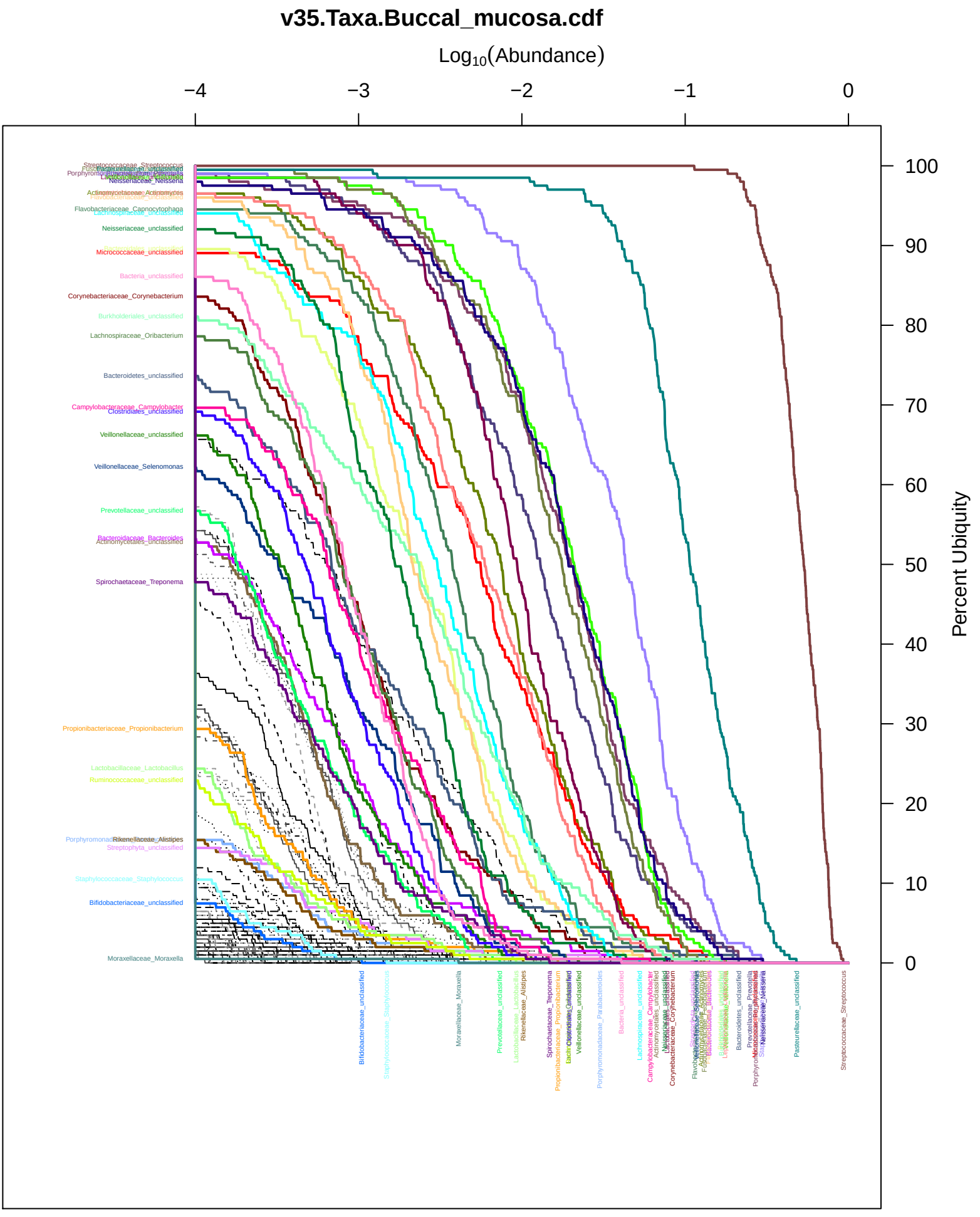
v35.Taxa.Buccal_mucosa.cdf
Log10(Abundance)
−4
−3
−2
−1
0
Percent Ubiquity
100
90
80
70
60
50
40
30
20
10
0
Streptococcaceae_Streptococcus
Neisseriaceae_Neisseria
Actinomycetaceae_Actinomyces
Flavobacteriaceae_Capnocytophaga
Neisseriaceae_unclassified
Micrococcaceae_unclassified
Bacteria_unclassified
Corynebacteriaceae_Corynebacterium
Burkholderiales_unclassified
Lachnospiraceae_Oribacterium
Bacteroidetes_unclassified
Campylobacteraceae_Campylobacter
Clostridiales_unclassified
Veillonellaceae_unclassified
Veillonellaceae_Selenomonas
Prevotellaceae_unclassified
Bacteroidaceae_Bacteroides
Actinomycetales_unclassified
Spirochaetaceae_Treponema
Propionibacteriaceae_Propionibacterium
Lactobacillaceae_Lactobacillus
Ruminococcaceae_unclassified
Rikenellaceae_Alistipes
Streptophyta_unclassified
Staphylococcaceae_Staphylococcus
Bifidobacteriaceae_unclassified
Moraxellaceae_Moraxella
Pasteurellaceae_unclassified

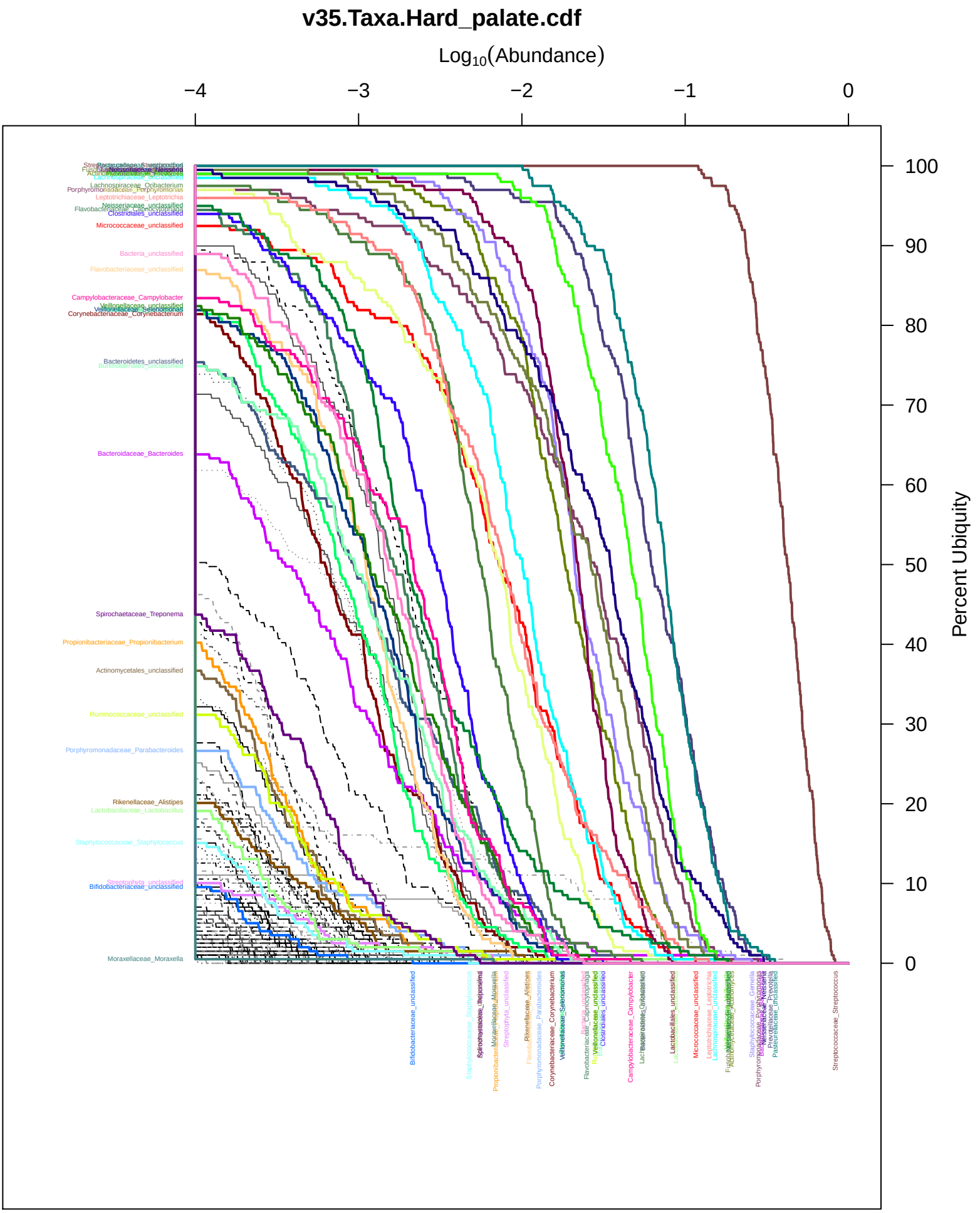
v35.Taxa.Hard_palate.cdf
Log10(Abundance)
−4
−3
−2
−1
0
Percent Ubiquity
100
90
80
70
60
50
40
30
20
10
0
Micrococcaceae_unclassified
Bacteria_unclassified
Flavobacteriaceae_unclassified
Campylobacteraceae_Campylobacter
Veillonellaceae_unclassified
Corynebacteriaceae_Corynebacterium
Bacteroidetes_unclassified
Bacteroidaceae_Bacteroides
Spirochaetaceae_Treponema
Propionibacteriaceae_Propionibacterium
Actinomycetales_unclassified
Ruminococcaceae_unclassified
Porphyromonadaceae_Parabacteroides
Rikenellaceae_Alistipes
Lactobacillaceae_Lactobacillus
Staphylococcaceae_Staphylococcus
Streptophyta_unclassified
Bifidobacteriaceae_unclassified
Moraxellaceae_Moraxella
Streptococcaceae_Streptococcus

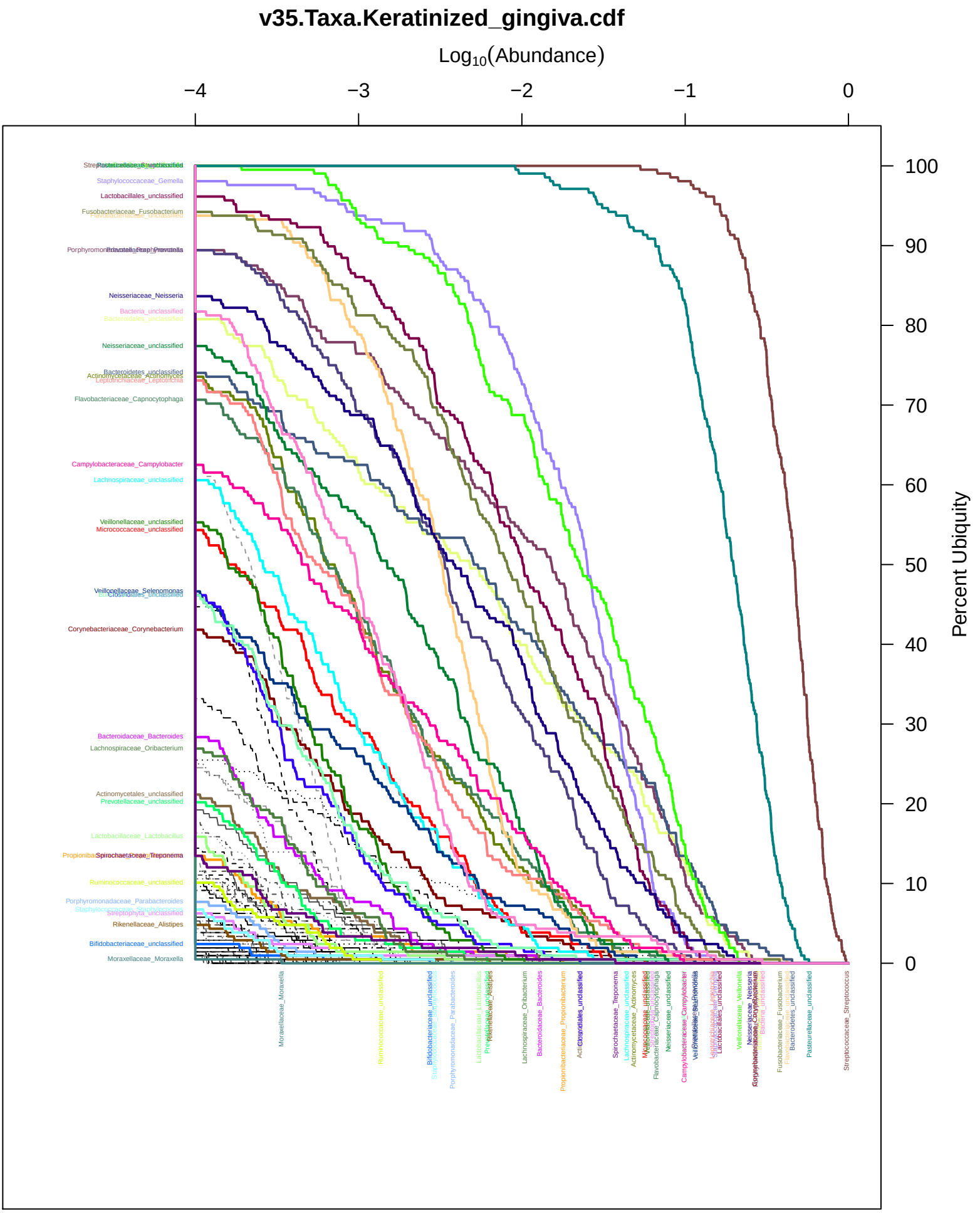
v35.Taxa.Keratinized_gingiva.cdf
Log10(Abundance)
−4
−3
−2
−1
0
Percent Ubiquity
100
90
80
70
60
50
40
30
20
10
0
Staphylococcaceae_Gemella
Lactobacillales_unclassified
Fusobacteriaceae_Fusobacterium
Neisseriaceae_Neisseria
Bacteria_unclassified
Bacteroidales_unclassified
Neisseriaceae_unclassified
Bacteroidetes_unclassified
Actinomycetaceae_Actinomyces
Leptotrichiaceae_Leptotrichia
Flavobacteriaceae_Capnocytophaga
Campylobacteraceae_Campylobacter
Lachnospiraceae_unclassified
Veillonellaceae_unclassified
Micrococcaceae_unclassified
Veillonellaceae_Selenomonas
Clostridiales_unclassified
Corynebacteriaceae_Corynebacterium
Bacteroidaceae_Bacteroides
Lachnospiraceae_Oribacterium
Actinomycetales_unclassified
Prevotellaceae_unclassified
Lactobacillaceae_Lactobacillus
Spirochaetaceae_Treponema
Ruminococcaceae_unclassified
Porphyromonadaceae_Parabacteroides
Streptophyta_unclassified
Rikenellaceae_Alistipes
Bifidobacteriaceae_unclassified
Moraxellaceae_Moraxella
Moraxellaceae_Moraxella
Ruminococcaceae_unclassified
Bifidobacteriaceae_unclassified
Porphyromonadaceae_Parabacteroides
Lachnospiraceae_Oribacterium
Bacteroidaceae_Bacteroides
Propionibacteriaceae_Propionibacterium
Spirochaetaceae_Treponema
Lachnospiraceae_unclassified
Actinomycetaceae_Actinomyces
Micrococcaceae_unclassified
Flavobacteriaceae_Capnocytophaga
Neisseriaceae_unclassified
Veillonellaceae_Veillonella
Neisseriaceae_Neisseria
Bacteria_unclassified
Fusobacteriaceae_Fusobacterium
Bacteroidetes_unclassified
Pasteurellaceae_unclassified
Streptococcaceae_Streptococcus

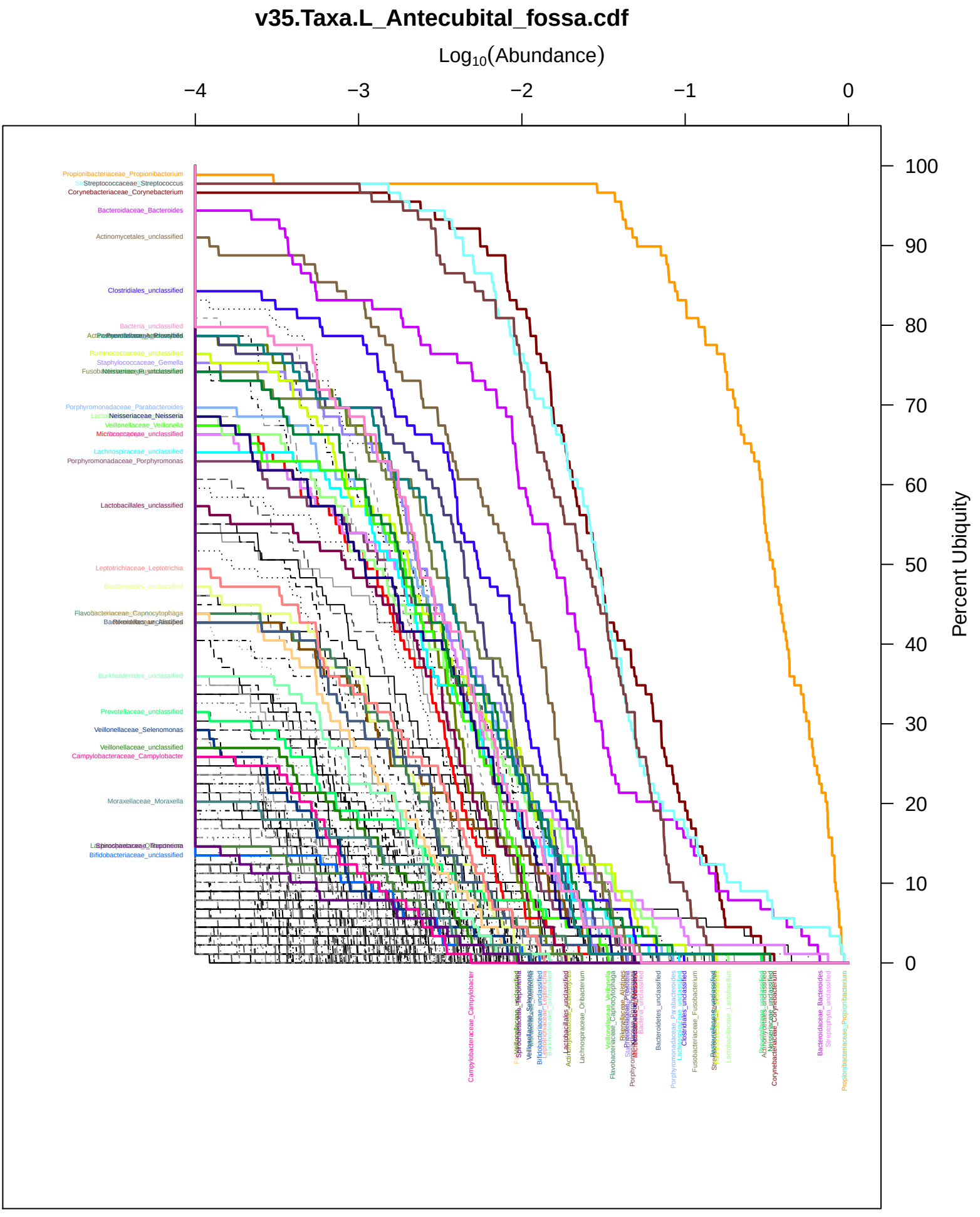
v35.Taxa.L_Antecubital_fossa.cdf
Log10(Abundance)
−4
−3
−2
−1
0
Percent Ubiquity
100
90
80
70
60
50
40
30
20
10
0
Propionibacteriaceae_Propionibacterium
Streptococcaceae_Streptococcus
Corynebacteriaceae_Corynebacterium
Bacteroidaceae_Bacteroides
Actinomycetales_unclassified
Clostridiales_unclassified
Bacteria_unclassified
Ruminococcaceae_unclassified
Staphylococcaceae_Gemella
Porphyromonadaceae_Parabacteroides
Neisseriaceae_Neisseria
Veillonellaceae_Veillonella
Micrococcaceae_unclassified
Lachnospiraceae_unclassified
Porphyromonadaceae_Porphyromonas
Lactobacillales_unclassified
Leptotrichiaceae_Leptotrichia
Bacteroidales_unclassified
Burkholderiales_unclassified
Prevotellaceae_unclassified
Veillonellaceae_Selenomonas
Veillonellaceae_unclassified
Campylobacteraceae_Campylobacter
Moraxellaceae_Moraxella
Bifidobacteriaceae_unclassified
Campylobacteraceae_Campylobacter
Lachnospiraceae_Oribacterium
Veillonellaceae_Veillonella
Bacteroidetes_unclassified
Porphyromonadaceae_Parabacteroides
Clostridiales_unclassified
Fusobacteriaceae_Fusobacterium
Lactobacillaceae_Lactobacillus
Actinomycetales_unclassified
Corynebacteriaceae_Corynebacterium
Bacteroidaceae_Bacteroides
Propionibacteriaceae_Propionibacterium

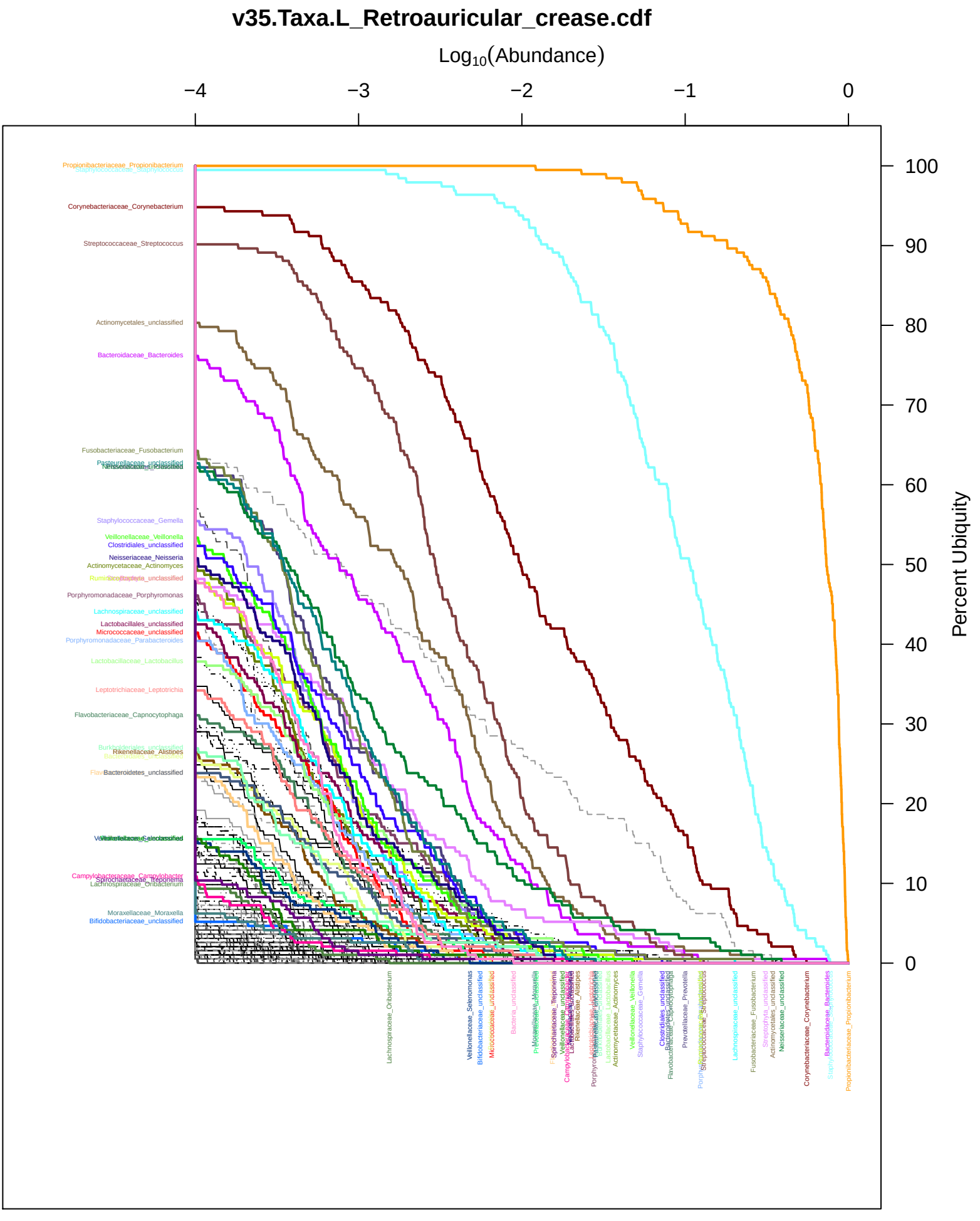
v35.Taxa.L_Retroauricular_crease.cdf
Log10(Abundance)
−4
−3
−2
−1
0
Percent Ubiquity
100
90
80
70
60
50
40
30
20
10
0
Propionibacteriaceae_Propionibacterium
Staphylococcaceae_Staphylococcus
Corynebacteriaceae_Corynebacterium
Streptococcaceae_Streptococcus
Actinomycetales_unclassified
Bacteroidaceae_Bacteroides
Fusobacteriaceae_Fusobacterium
Pasteurellaceae_unclassified
Staphylococcaceae_Gemella
Veillonellaceae_Veillonella
Clostridiales_unclassified
Neisseriaceae_Neisseria
Actinomycetaceae_Actinomyces
Porphyromonadaceae_Porphyromonas
Lachnospiraceae_unclassified
Lactobacillales_unclassified
Micrococcaceae_unclassified
Porphyromonadaceae_Parabacteroides
Lactobacillaceae_Lactobacillus
Leptotrichiaceae_Leptotrichia
Flavobacteriaceae_Capnocytophaga
Rikenellaceae_Alistipes
Bacteroidetes_unclassified
Veillonellaceae_Selenomonas
Spirochaetaceae_Treponema
Lachnospiraceae_Oribacterium
Moraxellaceae_Moraxella
Bifidobacteriaceae_unclassified

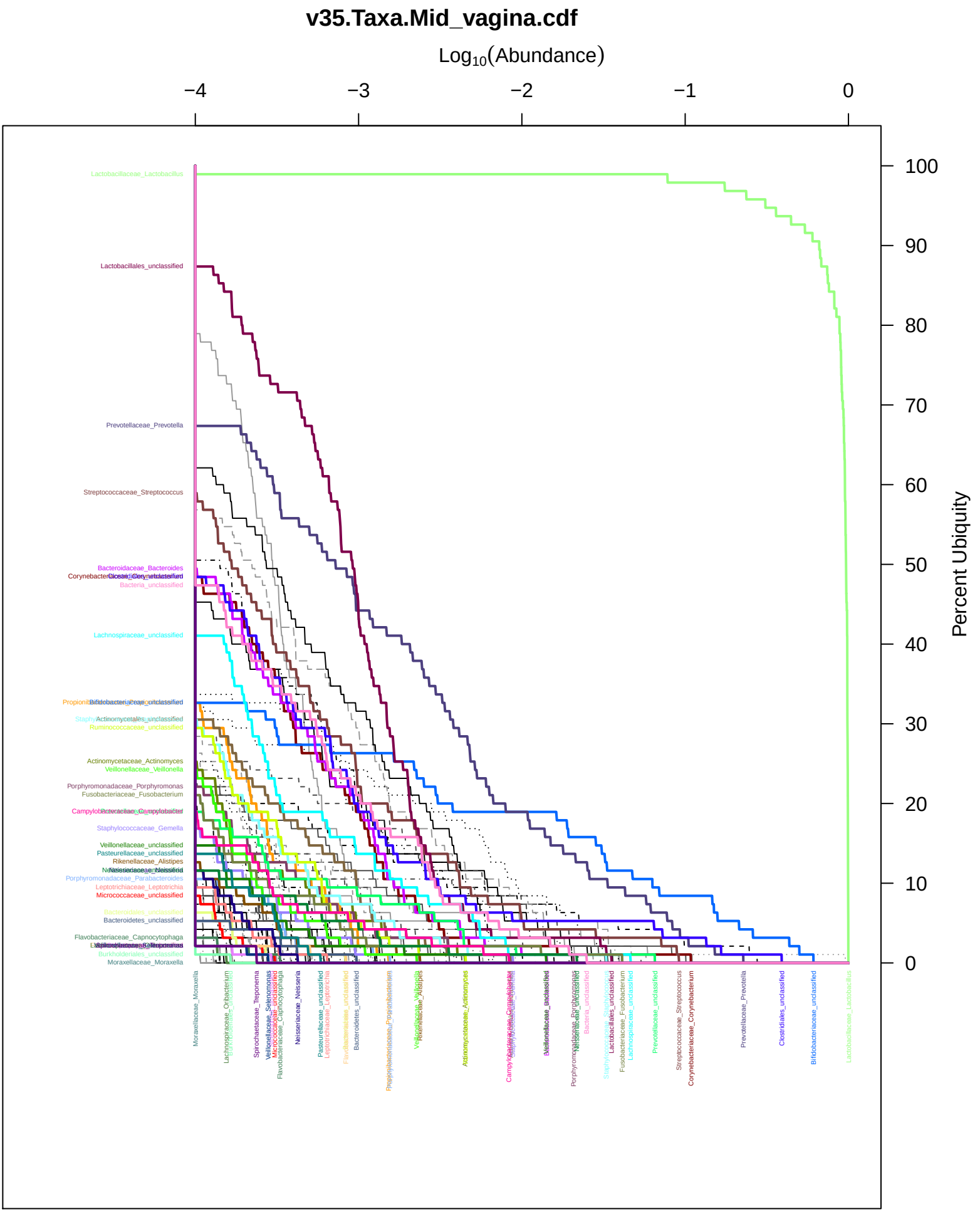
v35.Taxa.Mid_vagina.cdf
Log10(Abundance)
−4
−3
−2
−1
0
Percent Ubiquity
100
90
80
70
60
50
40
30
20
10
0
Lactobacillaceae_Lactobacillus
Lactobacillales_unclassified
Prevotellaceae_Prevotella
Streptococcaceae_Streptococcus
Bacteroidaceae_Bacteroides
Bacteria_unclassified
Lachnospiraceae_unclassified
Actinomycetaceae_Actinomyces
Veillonellaceae_Veillonella
Porphyromonadaceae_Porphyromonas
Fusobacteriaceae_Fusobacterium
Staphylococcaceae_Gemella
Veillonellaceae_unclassified
Pasteurellaceae_unclassified
Rikenellaceae_Alistipes
Porphyromonadaceae_Parabacteroides
Leptotrichiaceae_Leptotrichia
Micrococcaceae_unclassified
Bacteroidales_unclassified
Bacteroidetes_unclassified
Flavobacteriaceae_Capnocytophaga
Burkholderiales_unclassified
Moraxellaceae_Moraxella
Moraxellaceae_Moraxella
Lachnospiraceae_Cellulosilyticum
Spirochaetaceae_Treponema
Veillonellaceae_Selenomonas
Micrococcaceae_unclassified
Flavobacteriaceae_Capnocytophaga
Neisseriaceae_Neisseria
Pasteurellaceae_unclassified
Leptotrichiaceae_Leptotrichia
Flavobacteriaceae_unclassified
Bacteroidetes_unclassified
Propionibacteriaceae_Propionibacterium
Veillonellaceae_Veillonella
Rikenellaceae_Alistipes
Actinomycetaceae_Actinomyces
Veillonellaceae_unclassified
Porphyromonadaceae_Porphyromonas
Neisseriaceae_unclassified
Bacteria_unclassified
Staphylococcaceae_Gemella
Lactobacillales_unclassified
Fusobacteriaceae_Fusobacterium
Lachnospiraceae_unclassified
Prevotellaceae_unclassified
Streptococcaceae_Streptococcus
Corynebacteriaceae_Corynebacterium
Prevotellaceae_Prevotella
Clostridiales_unclassified
Bifidobacteriaceae_unclassified
Lactobacillaceae_Lactobacillus

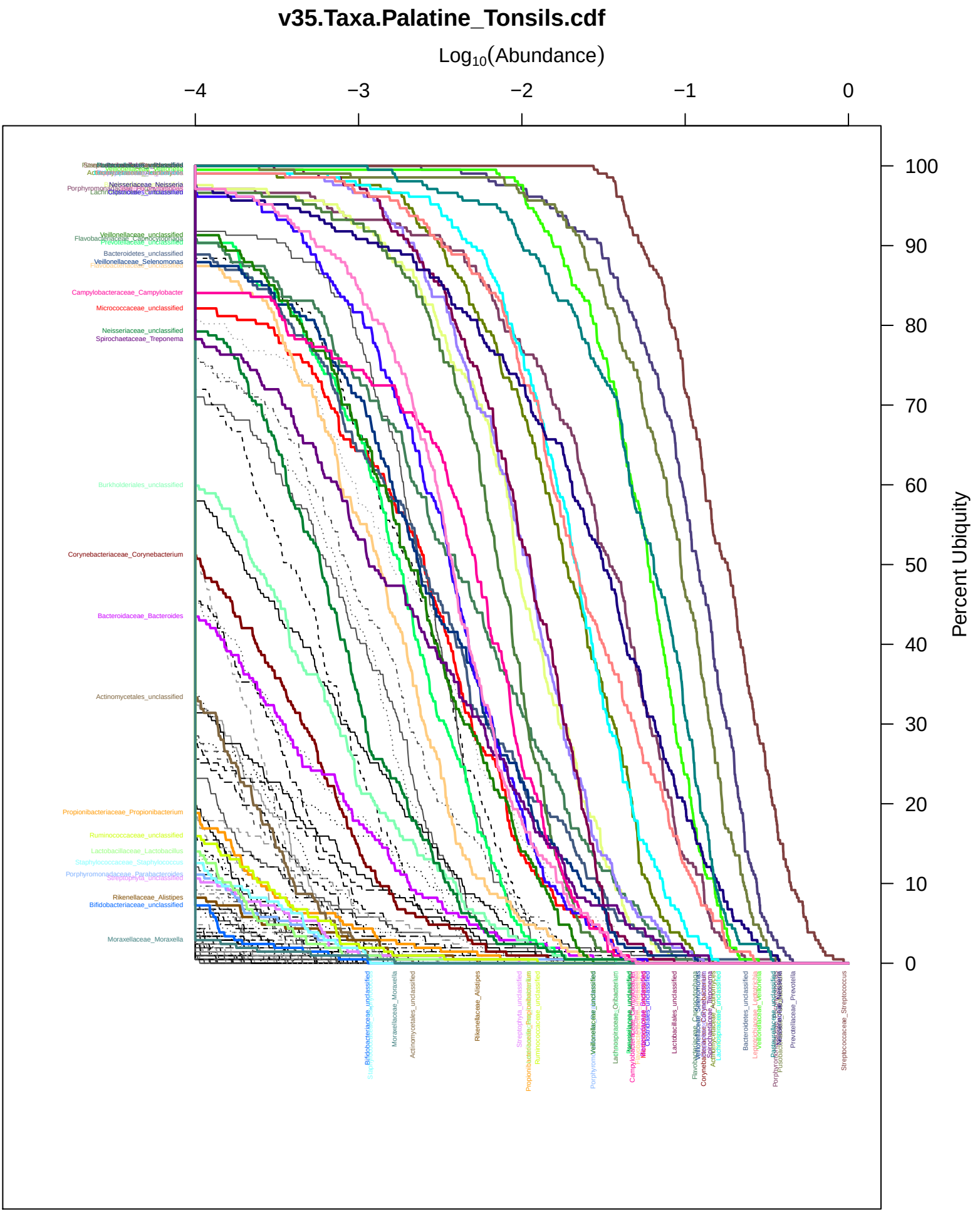
v35.Taxa.Palatine_Tonsils.cdf
Log10(Abundance)
−4
−3
−2
−1
0
Percent Ubiquity
100
90
80
70
60
50
40
30
20
10
0
Neisseriaceae_Neisseria
Veillonellaceae_unclassified
Prevotellaceae_unclassified
Bacteroidetes_unclassified
Veillonellaceae_Selenomonas
Campylobacteraceae_Campylobacter
Micrococcaceae_unclassified
Neisseriaceae_unclassified
Spirochaetaceae_Treponema
Burkholderiales_unclassified
Corynebacteriaceae_Corynebacterium
Bacteroidaceae_Bacteroides
Actinomycetales_unclassified
Propionibacteriaceae_Propionibacterium
Ruminococcaceae_unclassified
Lactobacillaceae_Lactobacillus
Staphylococcaceae_Staphylococcus
Porphyromonadaceae_Parabacteroides
Streptophyta_unclassified
Rikenellaceae_Alistipes
Bifidobacteriaceae_unclassified
Moraxellaceae_Moraxella
Bifidobacteriaceae_unclassified
Moraxellaceae_Moraxella
Actinomycetales_unclassified
Rikenellaceae_Alistipes
Streptophyta_unclassified
Propionibacteriaceae_Propionibacterium
Ruminococcaceae_unclassified
Lachnospiraceae_Coprococcus
Lachnospiraceae_unclassified
Lachnospiraceae_unclassified
Lactobacillales_unclassified
Corynebacteriaceae_Corynebacterium
Spirochaetaceae_Treponema
Actinomycetales_unclassified
Lachnospiraceae_unclassified
Bacteroidetes_unclassified
Veillonellaceae_Veillonella
Prevotellaceae_Prevotella
Streptococcaceae_Streptococcus

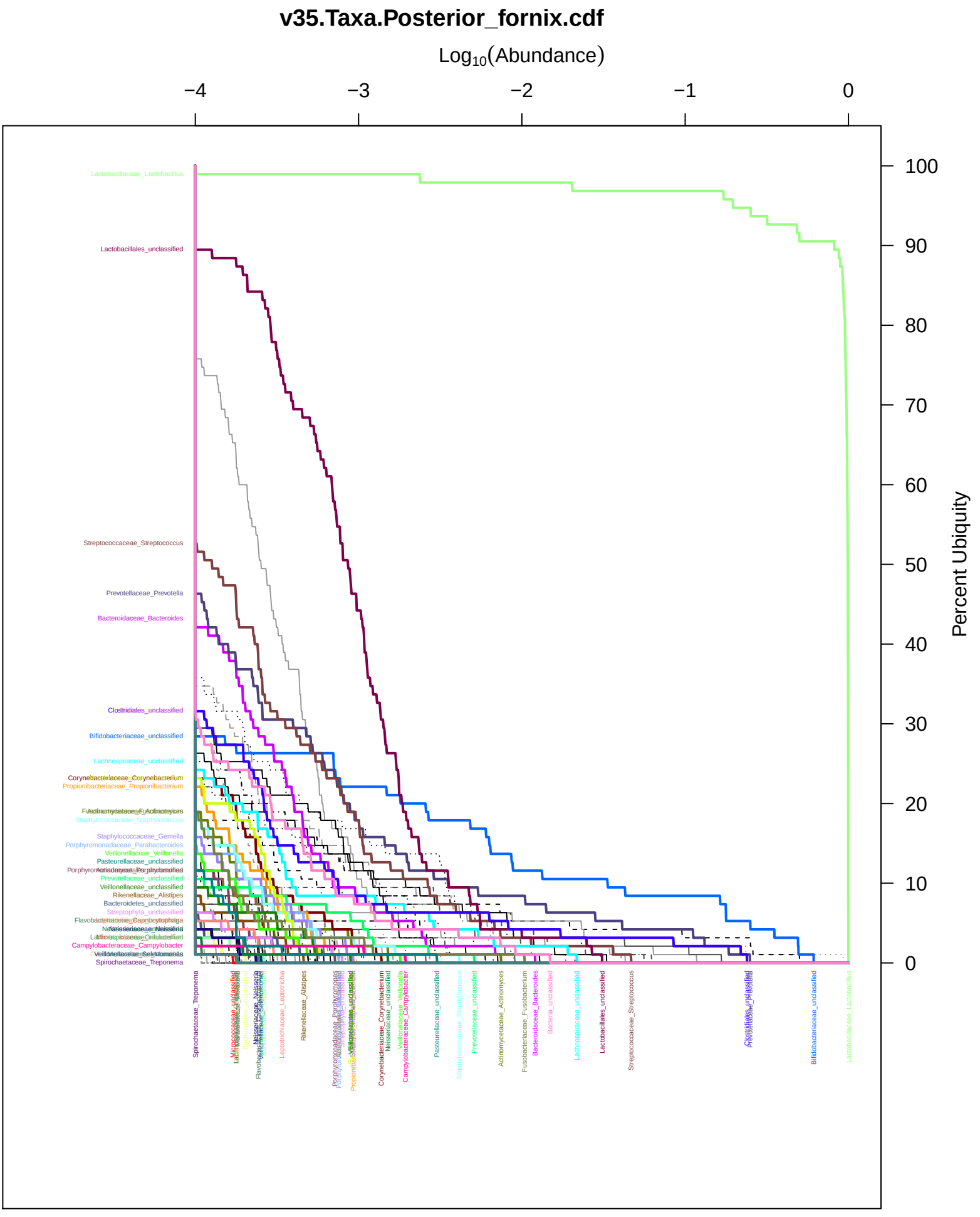
v35.Taxa.Posterior_fornix.cdf
Log10(Abundance)
−4
−3
−2
−1
0
Percent Ubiquity
100
90
80
70
60
50
40
30
20
10
0
Lactobacillaceae_Lactobacillus
Lactobacillales_unclassified
Streptococcaceae_Streptococcus
Prevotellaceae_Prevotella
Bacteroidaceae_Bacteroides
Clostridiales_unclassified
Bifidobacteriaceae_unclassified
Lachnospiraceae_unclassified
Corynebacteriaceae_Corynebacterium
Propionibacteriaceae_Propionibacterium
Staphylococcaceae_Staphylococcus
Staphylococcaceae_Gemella
Porphyromonadaceae_Parabacteroides
Veillonellaceae_Veillonella
Pasteurellaceae_unclassified
Prevotellaceae_unclassified
Veillonellaceae_unclassified
Rikenellaceae_Alistipes
Bacteroidetes_unclassified
Streptophyta_unclassified
Campylobacteraceae_Campylobacter
Spirochaetaceae_Treponema
Spirochaetaceae_Treponema
Leptotrichiaceae_Leptotrichia
Rikenellaceae_Alistipes
Pasteurellaceae_unclassified
Staphylococcaceae_Staphylococcus
Prevotellaceae_unclassified
Actinomycetaceae_Actinomyces
Fusobacteriaceae_Fusobacterium
Bacteroidaceae_Bacteroides
Bacteria_unclassified
Lachnospiraceae_unclassified
Lactobacillales_unclassified
Streptococcaceae_Streptococcus
Bifidobacteriaceae_unclassified
Lactobacillaceae_Lactobacillus

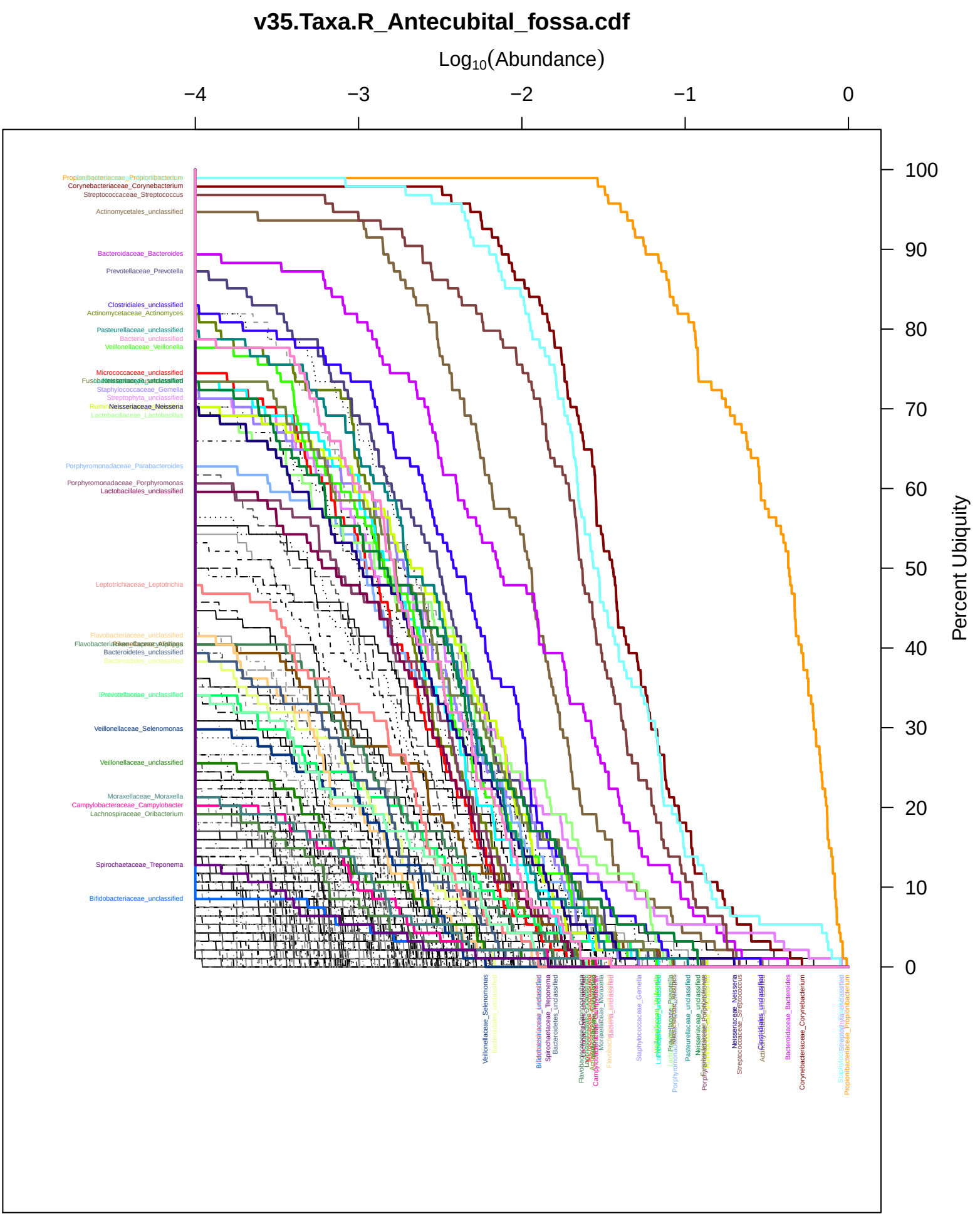
v35.Taxa.R_Antecubital_fossa.cdf
Log10(Abundance)
−4
−3
−2
−1
0
Percent Ubiquity
100
90
80
70
60
50
40
30
20
10
0
Propionibacteriaceae_Propionibacterium
Corynebacteriaceae_Corynebacterium
Streptococcaceae_Streptococcus
Actinomycetales_unclassified
Bacteroidaceae_Bacteroides
Prevotellaceae_Prevotella
Clostridiales_unclassified
Actinomycetaceae_Actinomyces
Pasteurellaceae_unclassified
Bacteria_unclassified
Veillonellaceae_Veillonella
Micrococcaceae_unclassified
Staphylococcaceae_Gemella
Streptophyta_unclassified
Neisseriaceae_Neisseria
Lactobacillaceae_Lactobacillus
Porphyromonadaceae_Parabacteroides
Porphyromonadaceae_Porphyromonas
Lactobacillales_unclassified
Leptotrichiaceae_Leptotrichia
Bacteroidetes_unclassified
Veillonellaceae_Selenomonas
Veillonellaceae_unclassified
Moraxellaceae_Moraxella
Campylobacteraceae_Campylobacter
Lachnospiraceae_Oribacterium
Spirochaetaceae_Treponema
Bifidobacteriaceae_unclassified

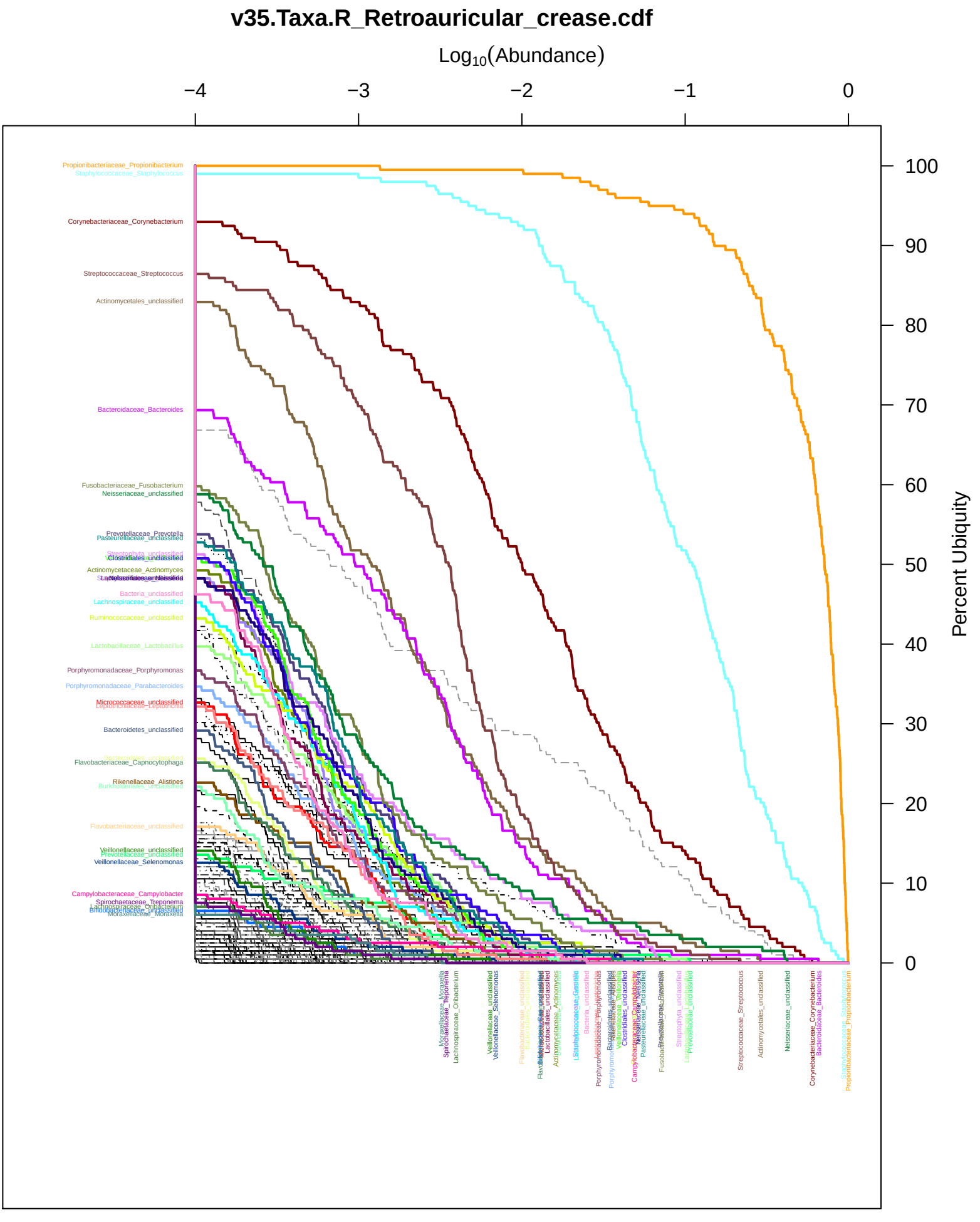
v35.Taxa.R_Retroauricular_crease.cdf
Log10(Abundance)
−4
−3
−2
−1
0
Percent Ubiquity
100
90
80
70
60
50
40
30
20
10
0
Propionibacteriaceae_Propionibacterium
Staphylococcaceae_Staphylococcus
Corynebacteriaceae_Corynebacterium
Streptococcaceae_Streptococcus
Actinomycetales_unclassified
Bacteroidaceae_Bacteroides
Fusobacteriaceae_Fusobacterium
Neisseriaceae_unclassified
Prevotellaceae_Prevotella
Pasteurellaceae_unclassified
Streptophyta_unclassified
Clostridiales_unclassified
Actinomycetaceae_Actinomyces
Bacteria_unclassified
Lachnospiraceae_unclassified
Ruminococcaceae_unclassified
Lactobacillaceae_Lactobacillus
Porphyromonadaceae_Porphyromonas
Porphyromonadaceae_Parabacteroides
Micrococcaceae_unclassified
Bacteroidetes_unclassified
Flavobacteriaceae_Capnocytophaga
Rikenellaceae_Alistipes
Burkholderiales_unclassified
Flavobacteriaceae_unclassified
Veillonellaceae_unclassified
Veillonellaceae_Selenomonas
Campylobacteraceae_Campylobacter
Spirochaetaceae_Treponema
Lachnospiraceae_Oribacterium
Bifidobacteriaceae_unclassified
Moraxellaceae_Moraxella

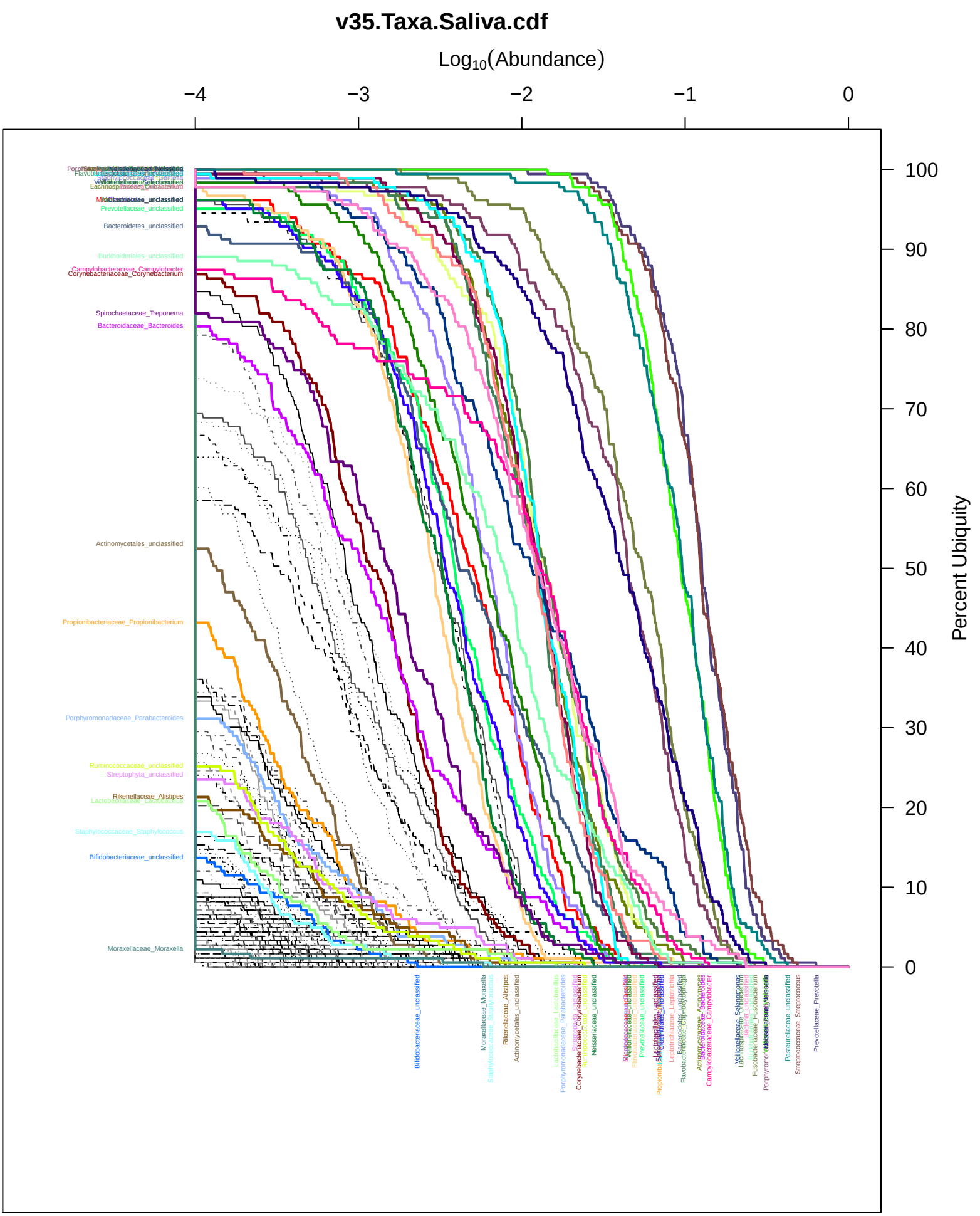
v35.Taxa.Saliva.cdf
Log10(Abundance)
−4
−3
−2
−1
0
Percent Ubiquity
100
90
80
70
60
50
40
30
20
10
0
Prevotellaceae_unclassified
Bacteroidetes_unclassified
Burkholderiales_unclassified
Campylobacteraceae_Campylobacter
Corynebacteriaceae_Corynebacterium
Spirochaetaceae_Treponema
Bacteroidaceae_Bacteroides
Actinomycetales_unclassified
Propionibacteriaceae_Propionibacterium
Porphyromonadaceae_Parabacteroides
Ruminococcaceae_unclassified
Streptophyta_unclassified
Rikenellaceae_Alistipes
Lactobacillaceae_Lactobacillus
Staphylococcaceae_Staphylococcus
Bifidobacteriaceae_unclassified
Moraxellaceae_Moraxella
Bifidobacteriaceae_unclassified
Moraxellaceae_Moraxella
Staphylococcaceae_Staphylococcus
Rikenellaceae_Alistipes
Actinomycetales_unclassified
Lactobacillaceae_Lactobacillus
Porphyromonadaceae_Parabacteroides
Streptophyta_unclassified
Corynebacteriaceae_Corynebacterium
Ruminococcaceae_unclassified
Neisseriaceae_unclassified
Veillonellaceae_unclassified
Prevotellaceae_unclassified
Propionibacteriaceae_Propionibacterium
Leptotrichiaceae_Leptotrichia
Bacteroidaceae_Bacteroides
Actinomycetales_unclassified
Campylobacteraceae_Campylobacter
Veillonellaceae_Selenomonas
Lachnospiraceae_Oribacterium
Bacteria_unclassified
Fusobacteriaceae_Fusobacterium
Porphyromonadaceae_Neisseria
Pasteurellaceae_unclassified
Streptococcaceae_Streptococcus
Prevotellaceae_Prevotella

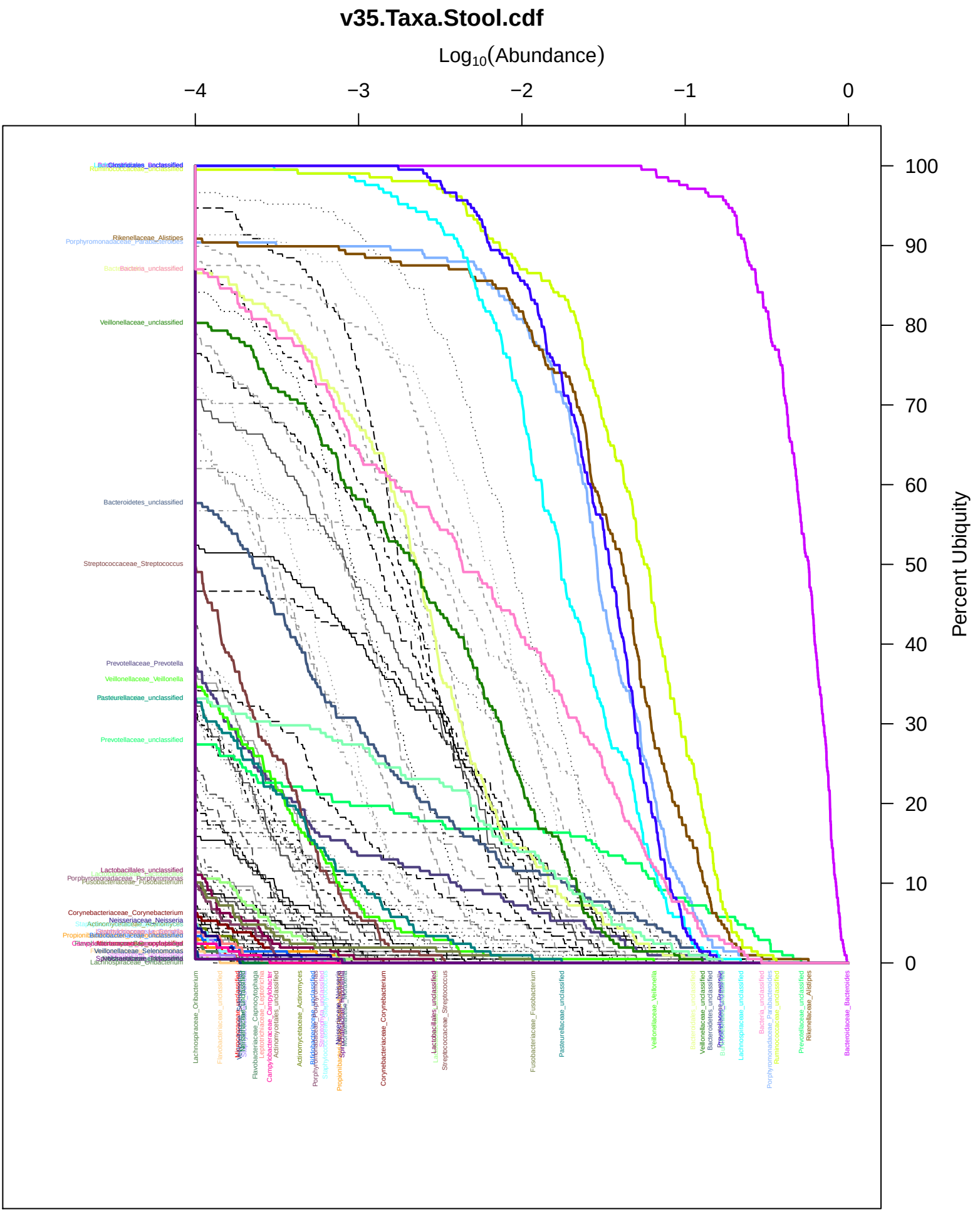
v35.Taxa.Stool.cdf
Log10(Abundance)
−4
−3
−2
−1
0
Percent Ubiquity
100
90
80
70
60
50
40
30
20
10
0
Rikenellaceae_Alistipes
Porphyromonadaceae_Parabacteroides
Veillonellaceae_unclassified
Bacteroidetes_unclassified
Streptococcaceae_Streptococcus
Prevotellaceae_Prevotella
Veillonellaceae_Veillonella
Pasteurellaceae_unclassified
Prevotellaceae_unclassified
Lactobacillales_unclassified
Porphyromonadaceae_Porphyromonas
Fusobacteriaceae_Fusobacterium
Corynebacteriaceae_Corynebacterium
Neisseriaceae_Neisseria
Lachnospiraceae_Oribacterium
Flavobacteriaceae_unclassified
Flavobacteriaceae_Capnocytophaga
Leptotrichiaceae_Leptotrichia
Actinomycetaceae_Actinomyces
Actinomycetales_unclassified
Porphyromonadaceae_unclassified
Lachnospiraceae_unclassified
Bacteria_unclassified
Ruminococcaceae_Parabacteroides
Ruminococcaceae_unclassified
Prevotellaceae_unclassified
Rikenellaceae_Alistipes
Bacteroidaceae_Bacteroides

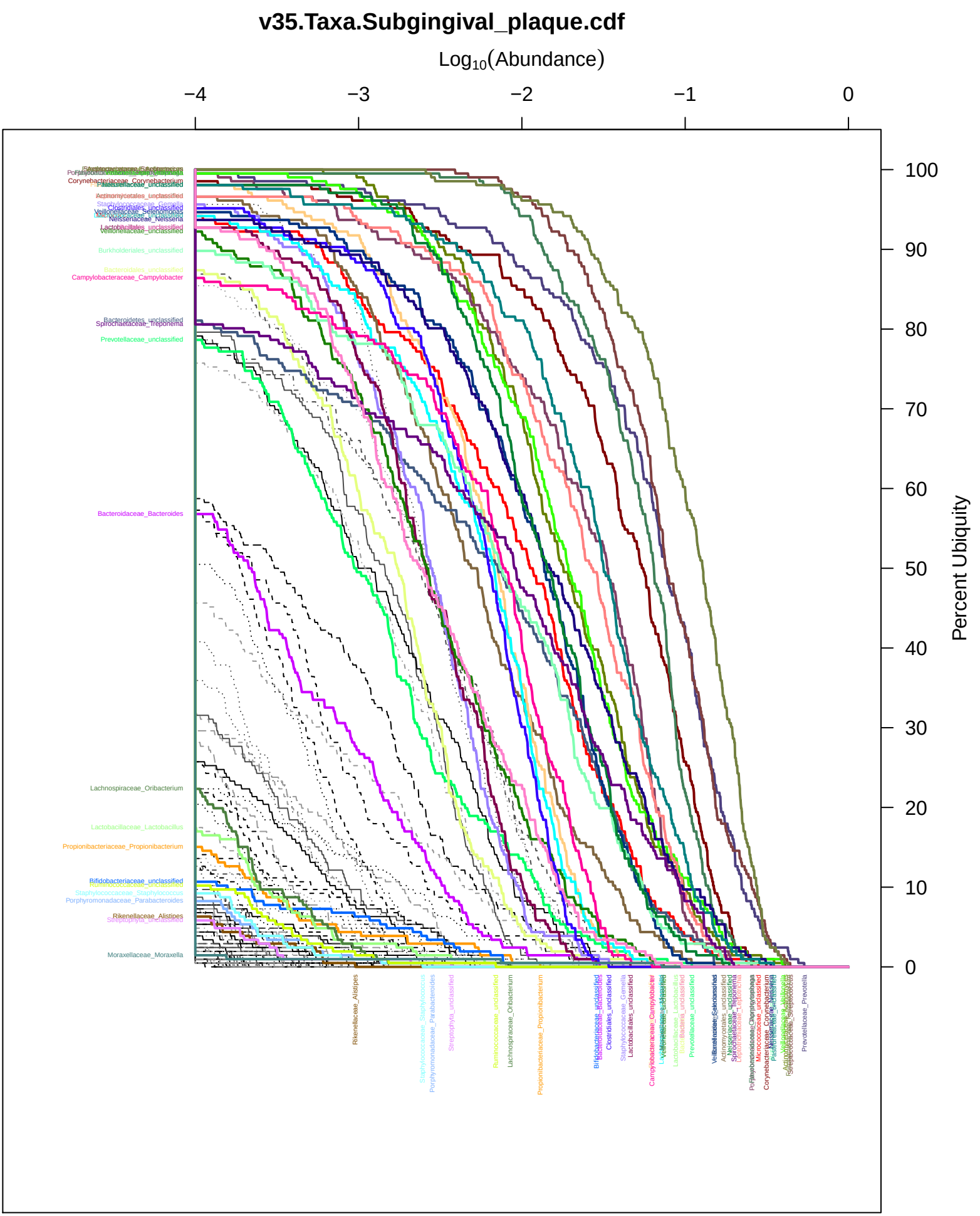
v35.Taxa.Subgingival_plaque.cdf
Log10(Abundance)
−4
−3
−2
−1
0
Percent Ubiquity
100
90
80
70
60
50
40
30
20
10
0
Corynebacteriaceae_Corynebacterium
Actinomycetales_unclassified
Veillonellaceae_Phascolarctobacterium
Neisseriaceae_Neisseria
Lactobacillales_unclassified
Veillonellaceae_unclassified
Burkholderiales_unclassified
Bacteroidales_unclassified
Campylobacteraceae_Campylobacter
Bacteroidetes_unclassified
Spirochaetaceae_Treponema
Prevotellaceae_unclassified
Bacteroidaceae_Bacteroides
Lachnospiraceae_Oribacterium
Lactobacillaceae_Lactobacillus
Propionibacteriaceae_Propionibacterium
Bifidobacteriaceae_unclassified
Staphylococcaceae_Staphylococcus
Porphyromonadaceae_Parabacteroides
Rikenellaceae_Alistipes
Moraxellaceae_Moraxella
Rikenellaceae_Alistipes
Staphylococcaceae_Staphylococcus
Porphyromonadaceae_Parabacteroides
Streptophyta_unclassified
Ruminococcaceae_unclassified
Lachnospiraceae_Oribacterium
Propionibacteriaceae_Propionibacterium
Bifidobacteriaceae_unclassified
Clostridiales_unclassified
Staphylococcaceae_Gemella
Lactobacillales_unclassified
Campylobacteraceae_Campylobacter
Lachnospiraceae_unclassified
Veillonellaceae_unclassified
Lactobacillaceae_Lactobacillus
Bacteroidaceae_unclassified
Prevotellaceae_unclassified
Actinomycetales_unclassified
Neisseriaceae_unclassified
Spirochaetaceae_Treponema
Corynebacteriaceae_Corynebacterium
Prevotellaceae_Prevotella

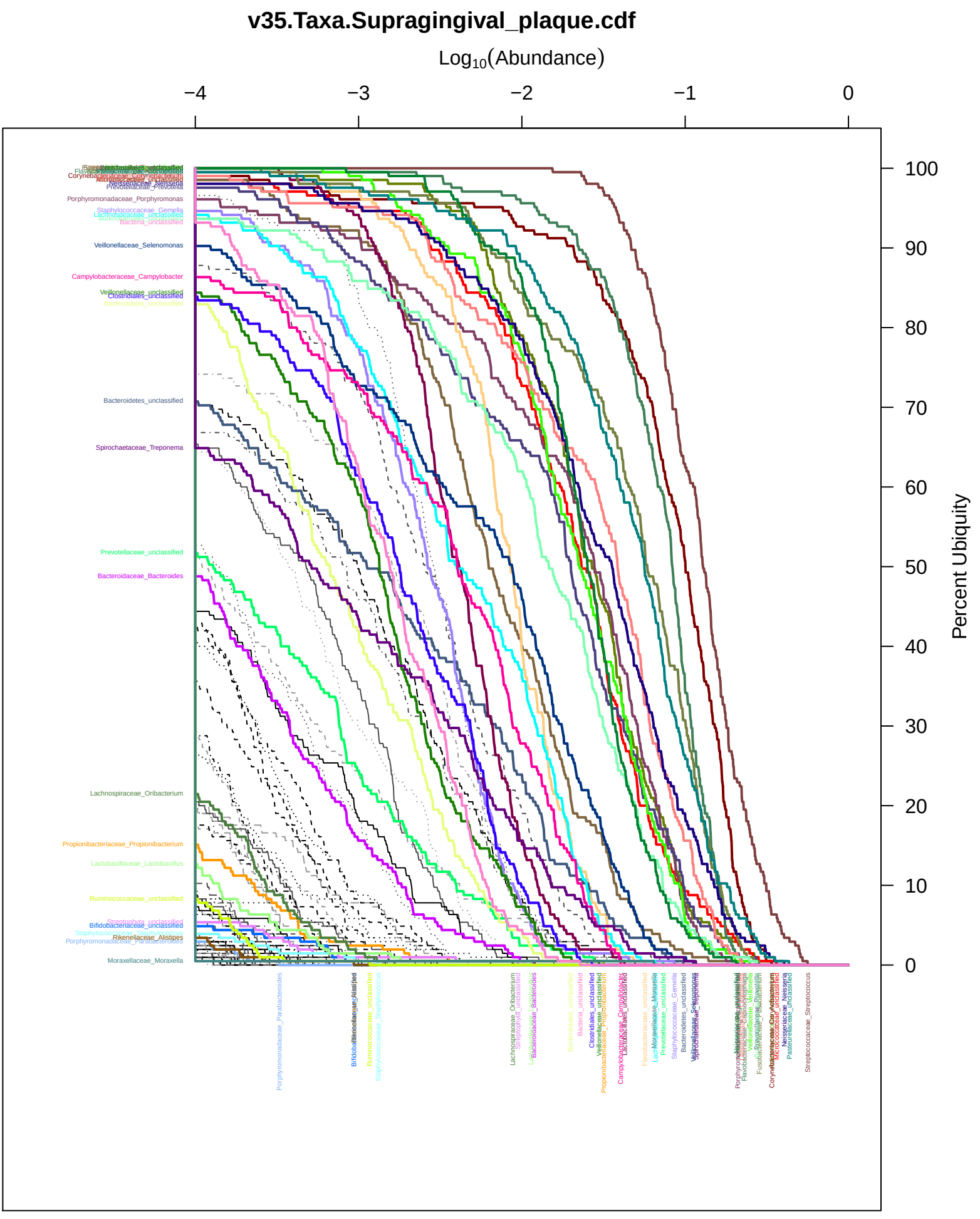
v35.Taxa.Supragingival_plaque.cdf
Log10(Abundance)
−4
−3
−2
−1
0
Percent Ubiquity
100
90
80
70
60
50
40
30
20
10
0
Porphyromonadaceae_Porphyromonas
Staphylococcaceae_Gemella
Veillonellaceae_Selenomonas
Campylobacteraceae_Campylobacter
Veillonellaceae_unclassified
Clostridiales_unclassified
Bacteroidetes_unclassified
Spirochaetaceae_Treponema
Prevotellaceae_unclassified
Bacteroidaceae_Bacteroides
Lachnospiraceae_Oribacterium
Propionibacteriaceae_Propionibacterium
Lactobacillaceae_Lactobacillus
Ruminococcaceae_unclassified
Bifidobacteriaceae_unclassified
Rikenellaceae_Alistipes
Moraxellaceae_Moraxella
Streptococcaceae_Streptococcus

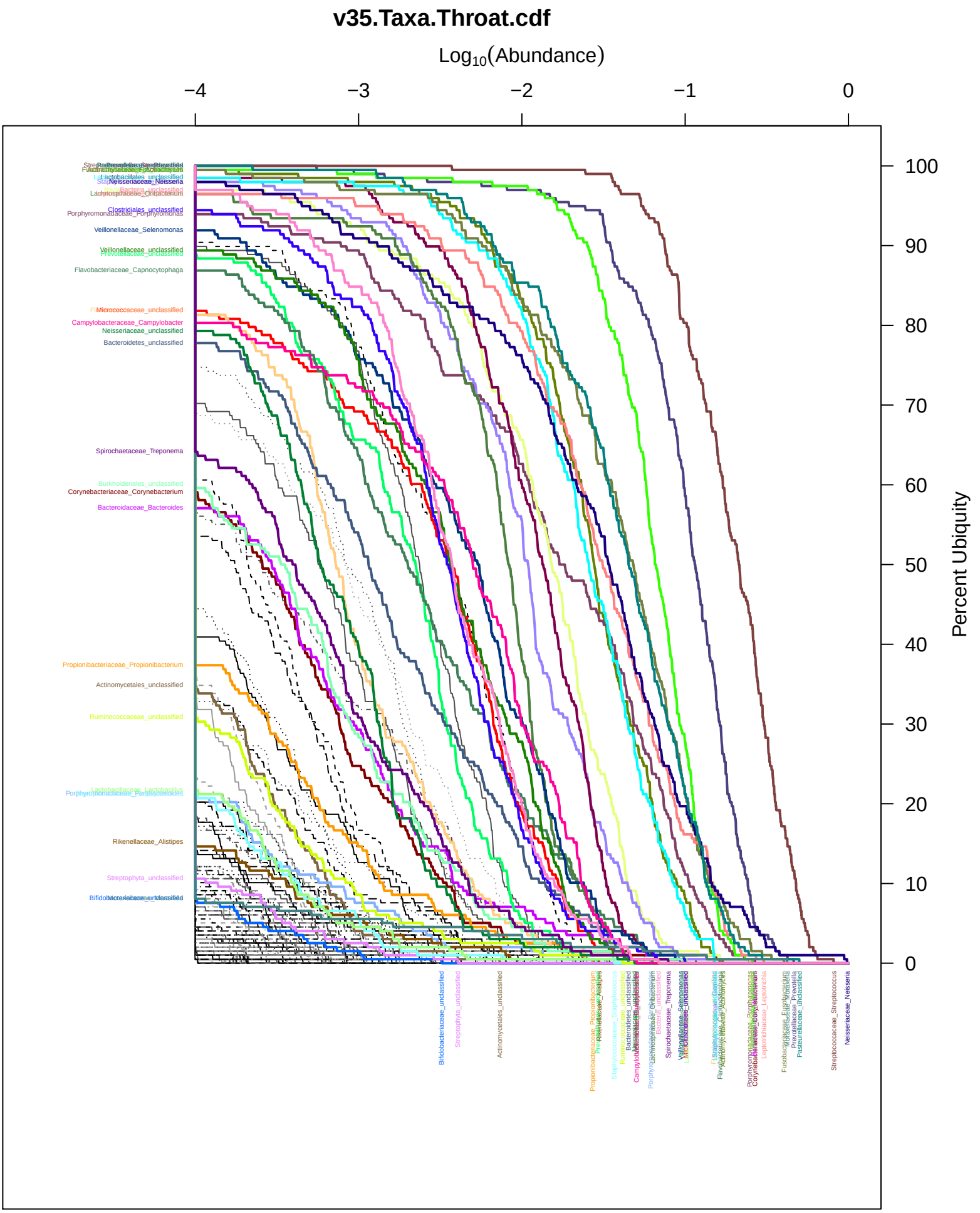
v35.Taxa.Throat.cdf
Log10(Abundance)
−4
−3
−2
−1
0
Percent Ubiquity
100
90
80
70
60
50
40
30
20
10
0
Lactobacillales_unclassified
Neisseriaceae_Neisseria
Lachnospiraceae_Oribacterium
Clostridiales_unclassified
Porphyromonadaceae_Porphyromonas
Veillonellaceae_Selenomonas
Veillonellaceae_unclassified
Prevotellaceae_unclassified
Flavobacteriaceae_Capnocytophaga
Micrococcaceae_unclassified
Campylobacteraceae_Campylobacter
Neisseriaceae_unclassified
Bacteroidetes_unclassified
Spirochaetaceae_Treponema
Burkholderiales_unclassified
Corynebacteriaceae_Corynebacterium
Bacteroidaceae_Bacteroides
Propionibacteriaceae_Propionibacterium
Actinomycetales_unclassified
Ruminococcaceae_unclassified
Lactobacillaceae_Lactobacillus
Porphyromonadaceae_Parabacteroides
Rikenellaceae_Alistipes
Streptophyta_unclassified
Bifidobacteriaceae_unclassified
Actinomycetales_unclassified
Propionibacteriaceae_Propionibacterium
Rikenellaceae_Alistipes
Staphylococcaceae_Staphylococcus
Ruminococcaceae_unclassified
Bacteroidales_unclassified
Campylobacteraceae_Campylobacter
Porphyromonadaceae_unclassified
Lachnospiraceae_Oribacterium
Bacteria_unclassified
Spirochaetaceae_Treponema
Veillonellaceae_Selenomonas
Clostridiales_unclassified
Micrococcaceae_unclassified
Flavobacteriaceae_Capnocytophaga
Actinomycetales_unclassified
Porphyromonadaceae_Porphyromonas
Corynebacteriaceae_Corynebacterium
Leptotrichiaceae_Leptotrichia
Fusobacteriaceae_Fusobacterium
Moraxellaceae_Moraxella
Prevotellaceae_Prevotella
Pasteurellaceae_unclassified
Streptococcaceae_Streptococcus
Neisseriaceae_Neisseria

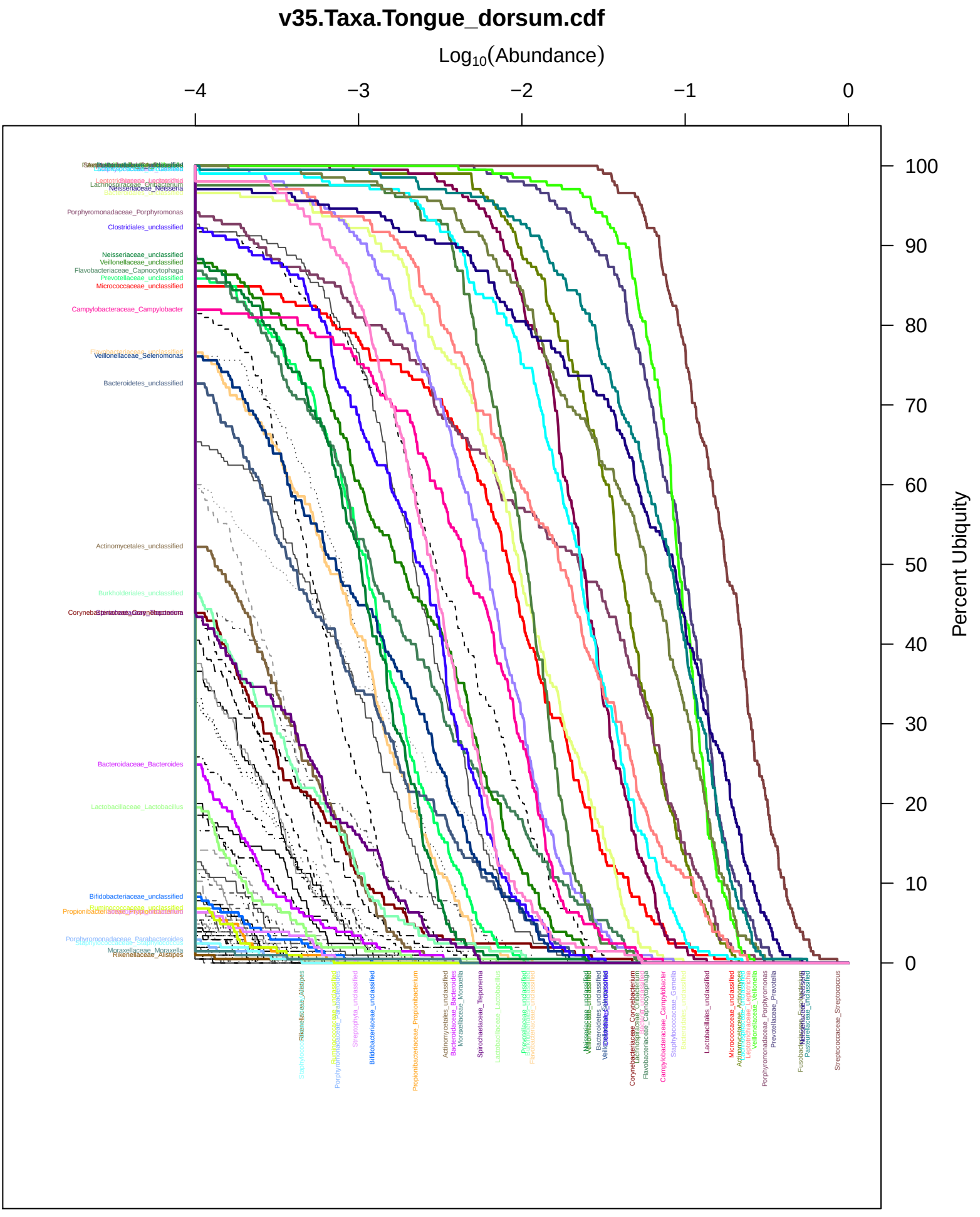
v35.Taxa.Tongue_dorsum.cdf
Log10(Abundance)
−4
−3
−2
−1
0
Percent Ubiquity
100
90
80
70
60
50
40
30
20
10
0
Porphyromonadaceae_Porphyromonas
Clostridiales_unclassified
Neisseriaceae_unclassified
Veillonellaceae_unclassified
Flavobacteriaceae_Capnocytophaga
Prevotellaceae_unclassified
Micrococcaceae_unclassified
Campylobacteraceae_Campylobacter
Veillonellaceae_Selenomonas
Bacteroidetes_unclassified
Actinomycetales_unclassified
Burkholderiales_unclassified
Bacteroidaceae_Bacteroides
Lactobacillaceae_Lactobacillus
Bifidobacteriaceae_unclassified
Ruminococcaceae_unclassified
Porphyromonadaceae_Parabacteroides
Moraxellaceae_Moraxella
Rikenellaceae_Alistipes
Streptophyta_unclassified
Propionibacteriaceae_Propionibacterium
Spirochaetaceae_Treponema
Prevotellaceae_unclassified
Corynebacteriaceae_Corynebacterium
Lachnospiraceae_Oribacterium
Actinomycetaceae_Actinomyces
Leptotrichiaceae_Leptotrichia
Pasteurellaceae_unclassified
Streptococcaceae_Streptococcus

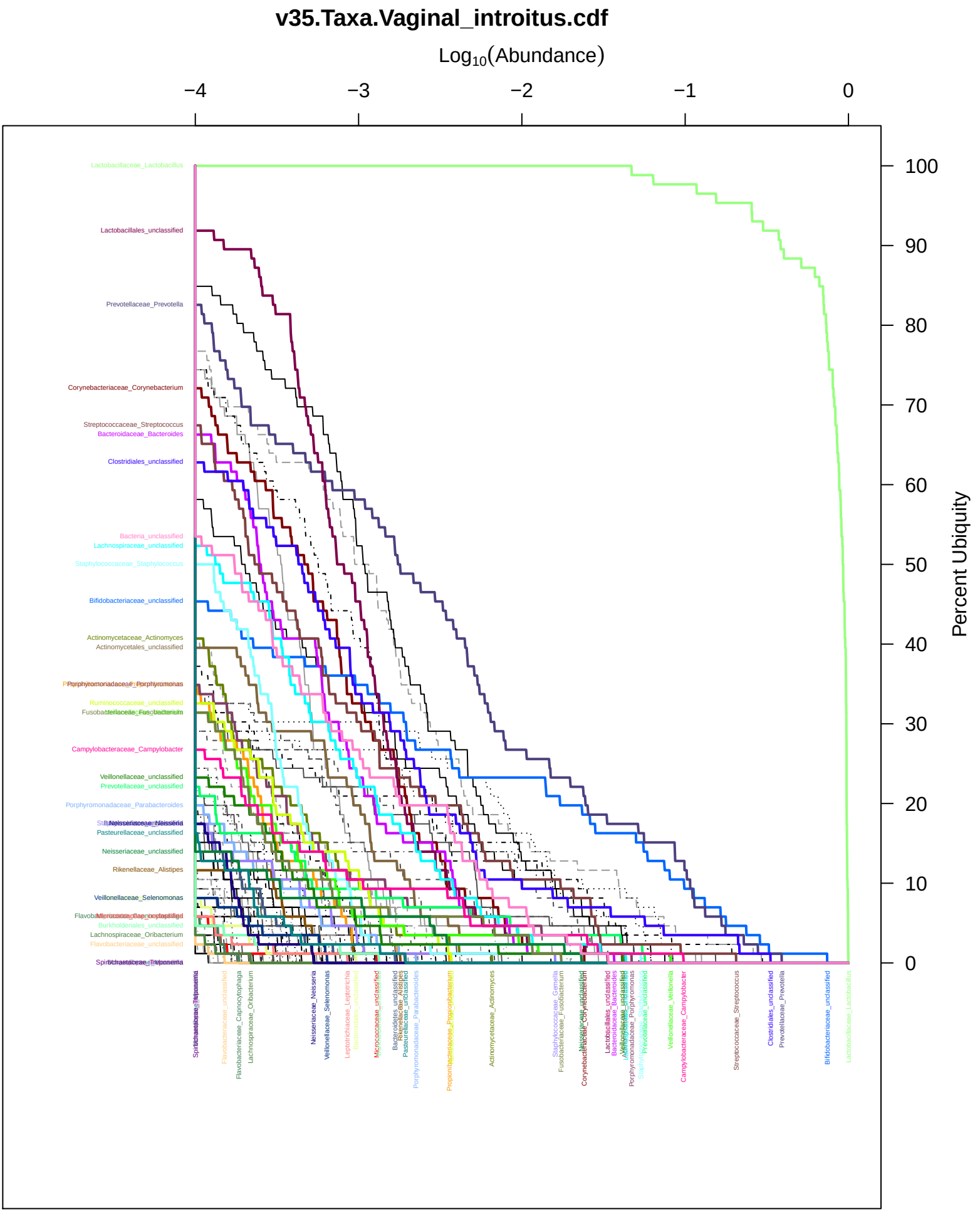
v35.Taxa.Vaginal_introitus.cdf
Log10(Abundance)
−4
−3
−2
−1
0
Percent Ubiquity
100
90
80
70
60
50
40
30
20
10
0
Lactobacillaceae_Lactobacillus
Lactobacillales_unclassified
Prevotellaceae_Prevotella
Corynebacteriaceae_Corynebacterium
Streptococcaceae_Streptococcus
Bacteroidaceae_Bacteroides
Clostridiales_unclassified
Bacteria_unclassified
Lachnospiraceae_unclassified
Staphylococcaceae_Staphylococcus
Bifidobacteriaceae_unclassified
Actinomycetaceae_Actinomyces
Actinomycetales_unclassified
Ruminococcaceae_unclassified
Campylobacteraceae_Campylobacter
Veillonellaceae_unclassified
Prevotellaceae_unclassified
Porphyromonadaceae_Parabacteroides
Pasteurellaceae_unclassified
Neisseriaceae_unclassified
Rikenellaceae_Alistipes
Veillonellaceae_Selenomonas
Burkholderiales_unclassified
Lachnospiraceae_Oribacterium
Flavobacteriaceae_unclassified
Micrococcaceae_unclassified
Leptotrichiaceae_Leptotrichia
Porphyromonadaceae_Porphyromonas
Fusobacteriaceae_Fusobacterium
Veillonellaceae_Veillonella
Flavobacteriaceae_Capnocytophaga
Neisseriaceae_Neisseria
Propionibacteriaceae_Propionibacterium
Staphylococcaceae_Gemella
Spirochaetaceae_Treponema
