# Supplementary material for: Analyses of the Stability and Core Taxonomic Memberships of the Human Microbiome
Source: PLoS One. 2013 May 6;8(5):e63139. doi: 10.1371/journal.pone.0063139 (PMC3646044; doi:10.1371/journal.pone.0063139)
Supplement: File S2 — Var-Ab Plots 18 Body Habitats. This PDF file contains the Var-Ab plots for all 18 HMP body habitats under study. Not all taxa were labeled in order to reduce clutter. See manuscript for additional details. (PDF) [file pone.0063139.s002.pdf]

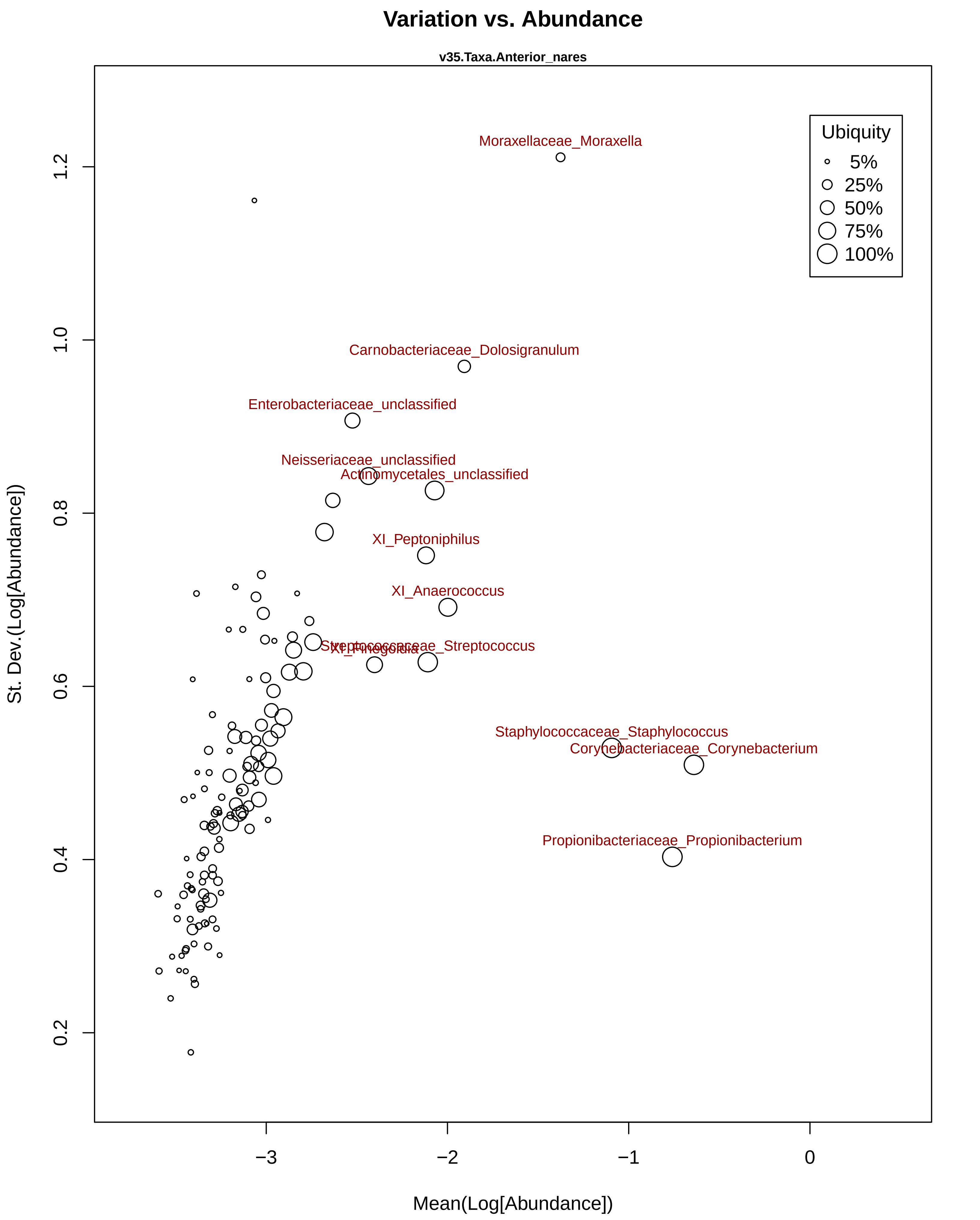
Variation vs. Abundance
v35.Taxa.Anterior_nares
Ubiquity
5%
25%
50%
75%
100%
Moraxellaceae_Moraxella
Carnobacteriaceae_Dolosigranulum
Enterobacteriaceae_unclassified
Neisseriaceae_unclassified
Actinomycetales_unclassified
XI_Peptoniphilus
XI_Anaerococcus
Streptococcaceae_Streptococcus
XI_Finegoldia
Staphylococcaceae_Staphylococcus
Corynebacteriaceae_Corynebacterium
Propionibacteriaceae_Propionibacterium
St. Dev.(Log[Abundance])
Mean(Log[Abundance])
1.2
1.0
0.8
0.6
0.4
0.2
−3
−2
−1
0

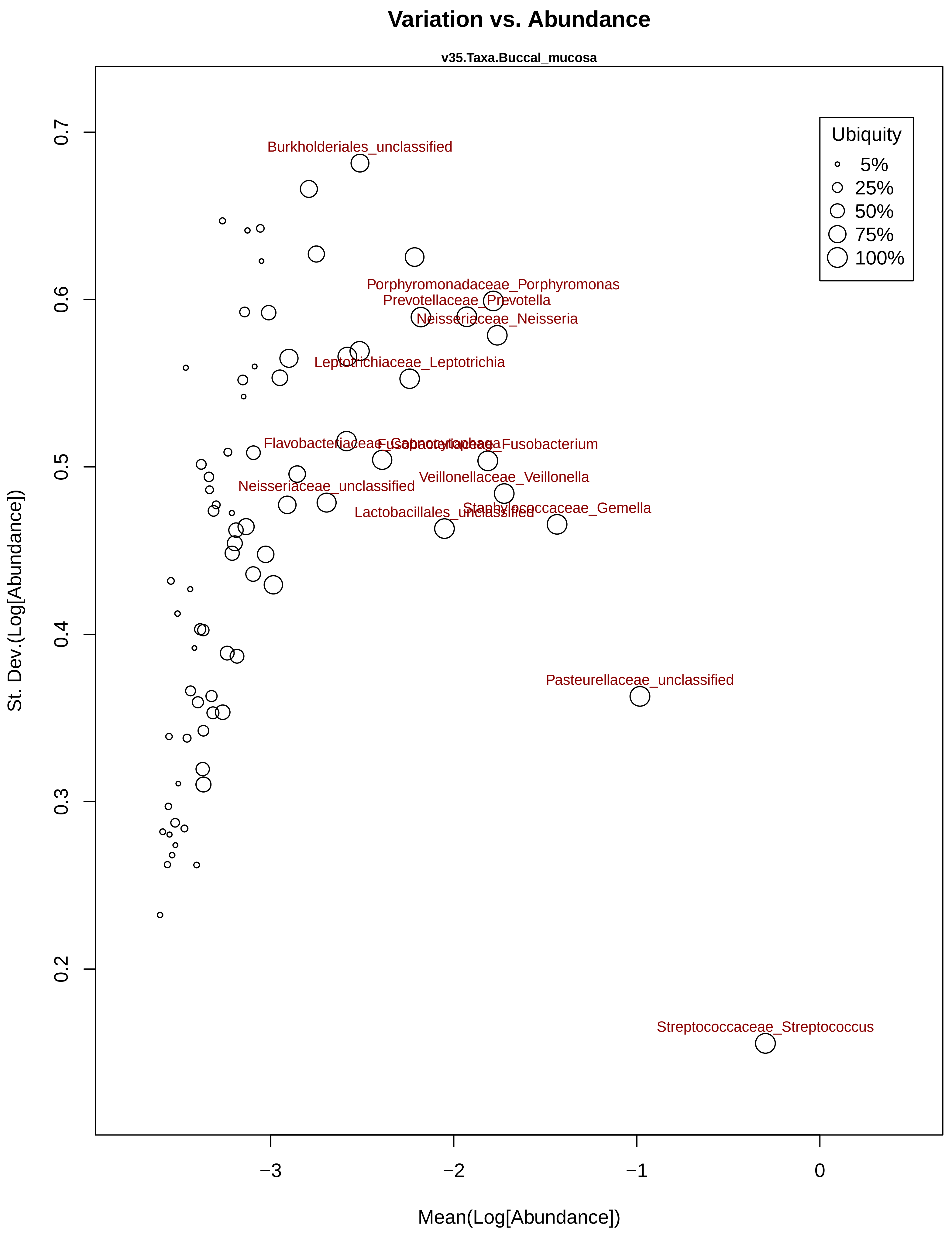
Variation vs. Abundance
v35.Taxa.Buccal_mucosa
Ubiquity
5%
25%
50%
75%
100%
Burkholderiales_unclassified
Porphyromonadaceae_Porphyromonas
Prevotellaceae_Prevotella
Neisseriaceae_Neisseria
Leptotrichiaceae_Leptotrichia
Flavobacteriaceae_Capnocytophaga
Fusobacteriaceae_Fusobacterium
Veillonellaceae_Veillonella
Neisseriaceae_unclassified
Lactobacillales_unclassified
Staphylococcaceae_Gemella
Pasteurellaceae_unclassified
Streptococcaceae_Streptococcus
St. Dev.(Log[Abundance])
Mean(Log[Abundance])
0.7
0.6
0.5
0.4
0.3
0.2
−3
−2
−1
0

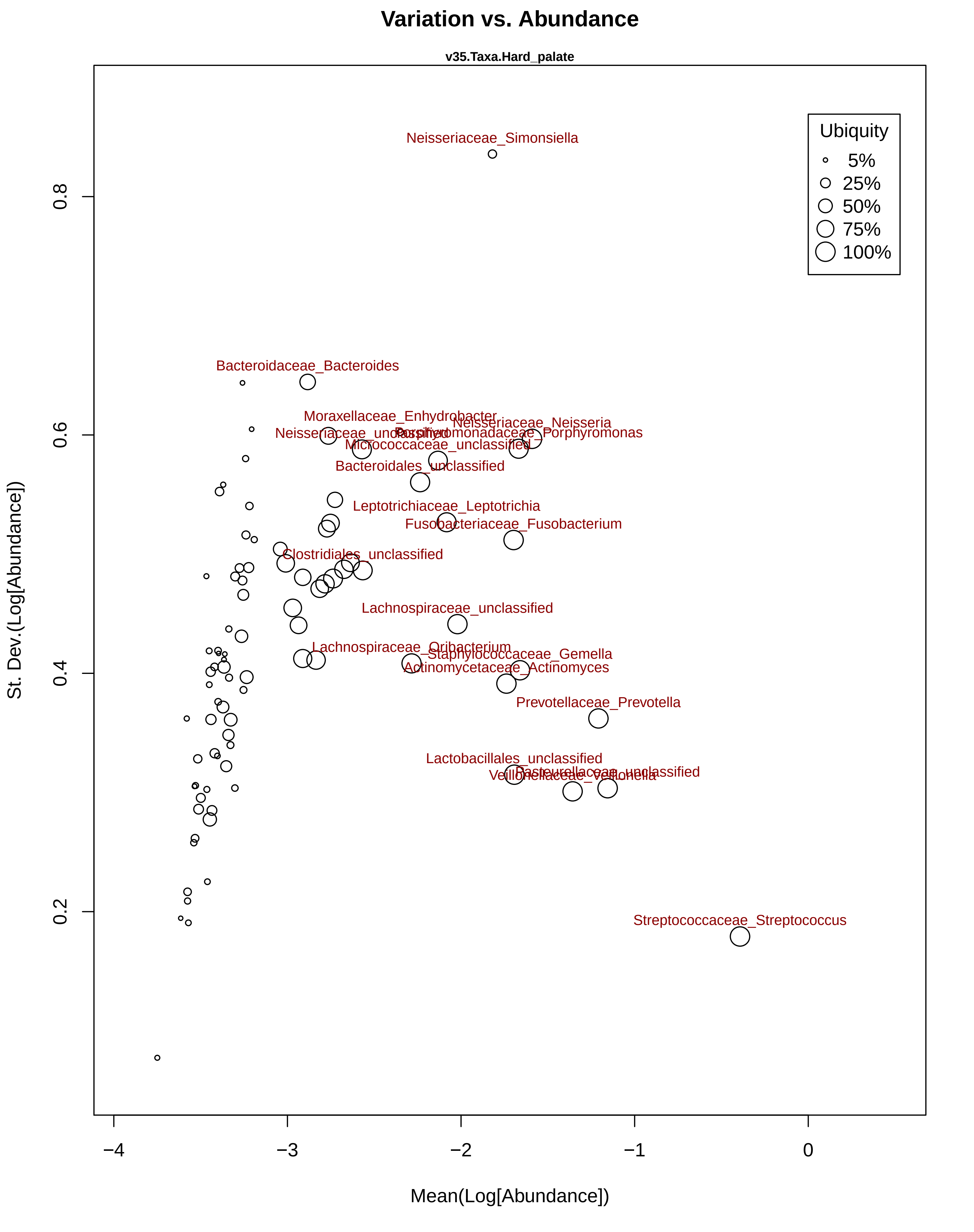

Variation vs. Abundance
v35.Taxa.Hard_palate
Ubiquity
5%
25%
50%
75%
100%
Neisseriaceae_Simonsiella
Bacteroidaceae_Bacteroides
Moraxellaceae_Enhydrobacter
Neisseriaceae_Neisseria
Porphyromonadaceae_Porphyromonas
Micrococcaceae_unclassified
Bacteroidales_unclassified
Leptotrichiaceae_Leptotrichia
Fusobacteriaceae_Fusobacterium
Clostridiales_unclassified
Lachnospiraceae_unclassified
Lachnospiraceae_Oribacterium
Staphylococcaceae_Gemella
Actinomycetaceae_Actinomyces
Prevotellaceae_Prevotella
Lactobacillales_unclassified
Pasteurellaceae_unclassified
Veillonellaceae_Veillonella
Streptococcaceae_Streptococcus
St. Dev.(Log[Abundance])
0.2
0.4
0.6
0.8
Mean(Log[Abundance])
−4
−3
−2
−1
0

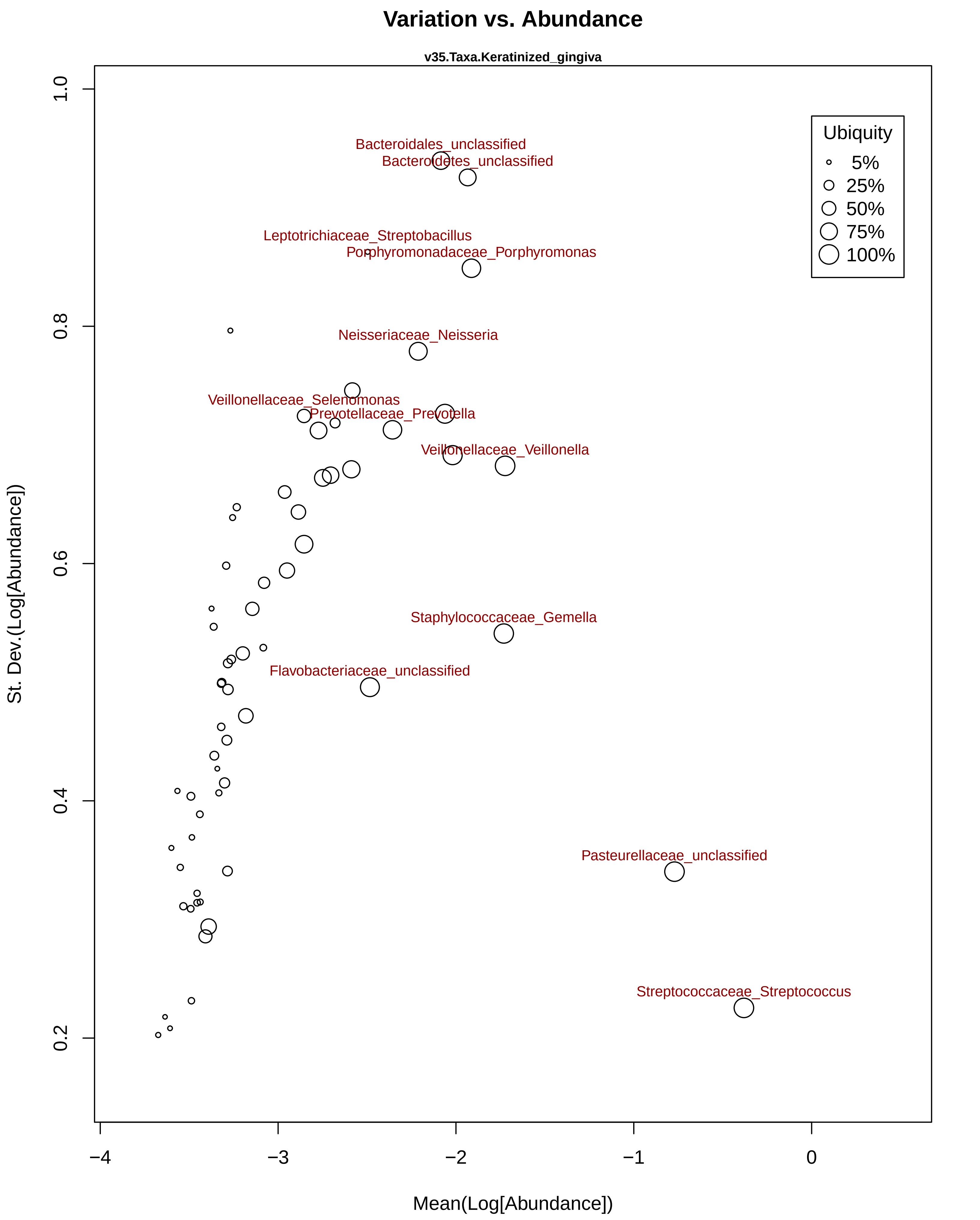

Variation vs. Abundance
v35.Taxa.Keratinized_gingiva
Ubiquity
5%
25%
50%
75%
100%
Bacteroidales_unclassified
Bacteroidetes_unclassified
Leptotrichiaceae_Streptobacillus
Porphyromonadaceae_Porphyromonas
Neisseriaceae_Neisseria
Veillonellaceae_Selenomonas
Prevotellaceae_Prevotella
Veillonellaceae_Veillonella
Staphylococcaceae_Gemella
Flavobacteriaceae_unclassified
Pasteurellaceae_unclassified
Streptococcaceae_Streptococcus
St. Dev.(Log[Abundance])
Mean(Log[Abundance])
1.0
0.8
0.6
0.4
0.2
−4
−3
−2
−1
0

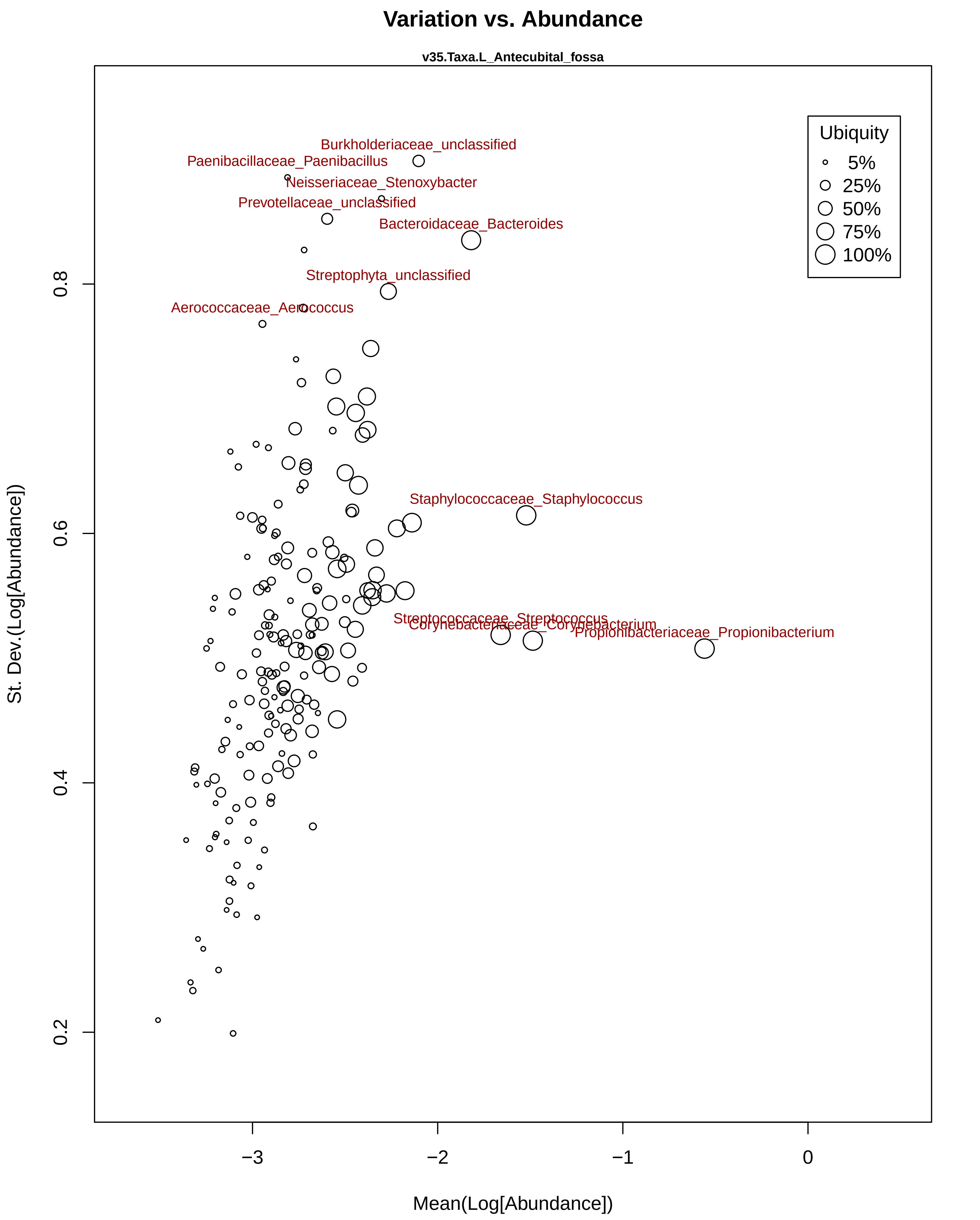
Variation vs. Abundance
v35.Taxa.L_Antecubital_fossa
Ubiquity
5%
25%
50%
75%
100%
Burkholderiaceae_unclassified
Paenibacillaceae_Paenibacillus
Neisseriaceae_Stenoxybacter
Prevotellaceae_unclassified
Bacteroidaceae_Bacteroides
Streptophyta_unclassified
Aerococcaceae_Aerococcus
Staphylococcaceae_Staphylococcus
Streptococcaceae_Streptococcus
Corynebacteriaceae_Corynebacterium
Propionibacteriaceae_Propionibacterium
St. Dev.(Log[Abundance])
0.2
0.4
0.6
0.8
Mean(Log[Abundance])
−3
−2
−1
0

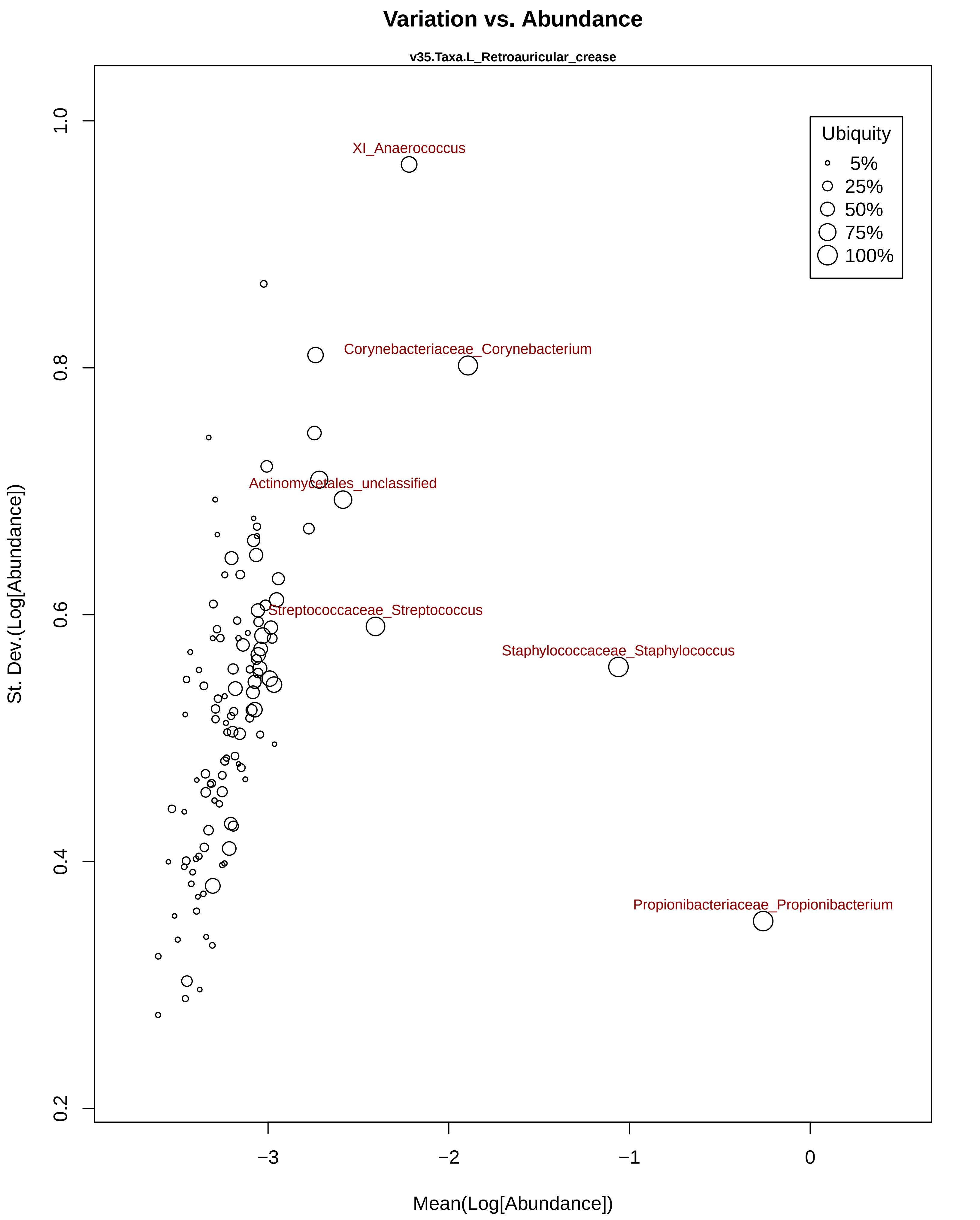
Variation vs. Abundance
v35.Taxa.L_Retroauricular_crease
Ubiquity
5%
25%
50%
75%
100%
XI_Anaerococcus
Corynebacteriaceae_Corynebacterium
Actinomycetales_unclassified
Streptococcaceae_Streptococcus
Staphylococcaceae_Staphylococcus
Propionibacteriaceae_Propionibacterium
1.0
0.8
0.6
0.4
0.2
−3
−2
−1
0
St. Dev.(Log[Abundance])
Mean(Log[Abundance])

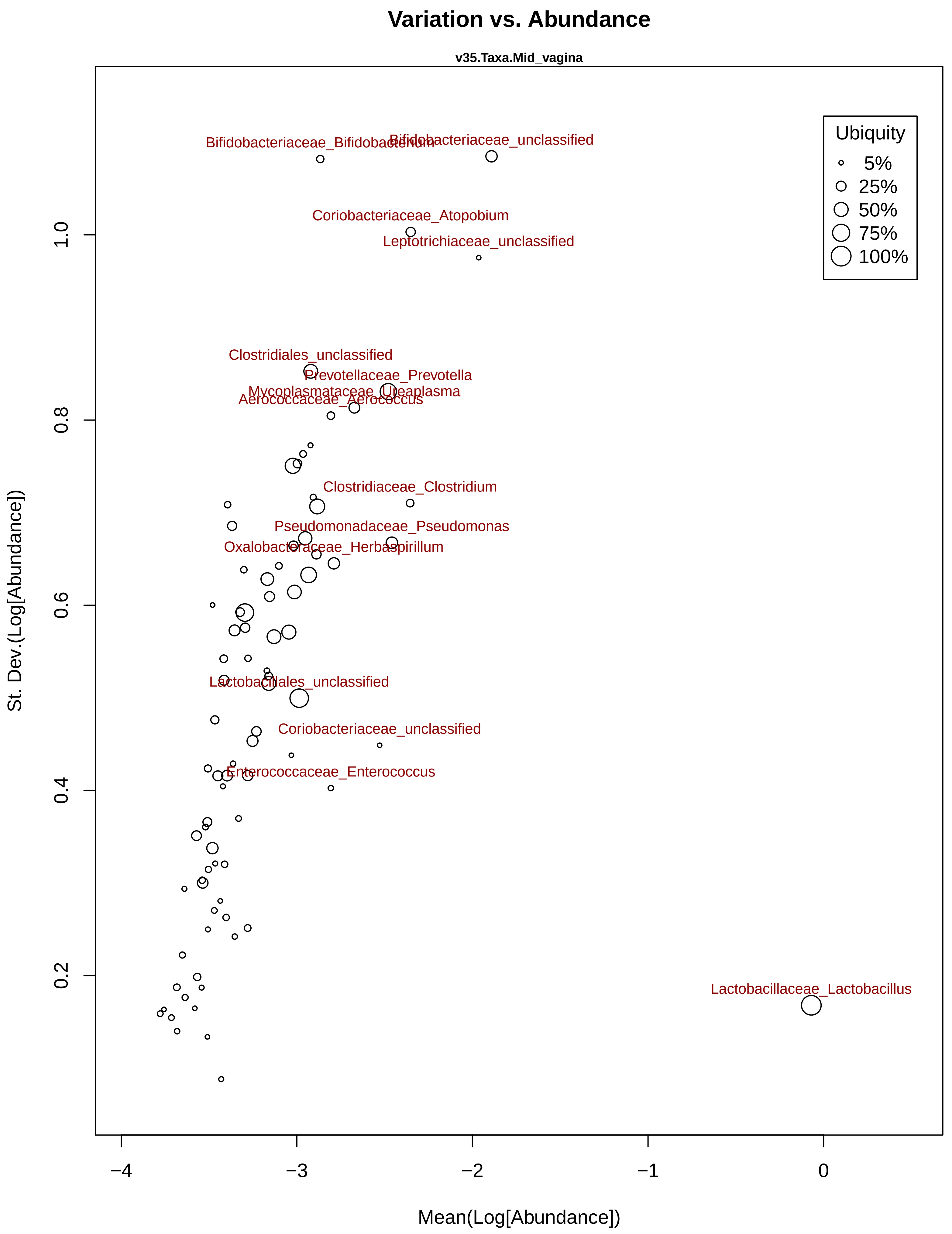

Variation vs. Abundance
v35.Taxa.Mid_vagina
Ubiquity
5%
25%
50%
75%
100%
Bifidobacteriaceae_Bifidobacterium
Bifidobacteriaceae_unclassified
Coriobacteriaceae_Atopobium
Leptotrichiaceae_unclassified
Clostridiales_unclassified
Prevotellaceae_Prevotella
Mycoplasmataceae_Ureaplasma
Aerococcaceae_Aerococcus
Clostridiaceae_Clostridium
Pseudomonadaceae_Pseudomonas
Oxalobacteraceae_Herbaspirillum
Lactobacillales_unclassified
Coriobacteriaceae_unclassified
Enterococcaceae_Enterococcus
Lactobacillaceae_Lactobacillus
St. Dev.(Log[Abundance])
Mean(Log[Abundance])
1.0
0.8
0.6
0.4
0.2
−4
−3
−2
−1
0

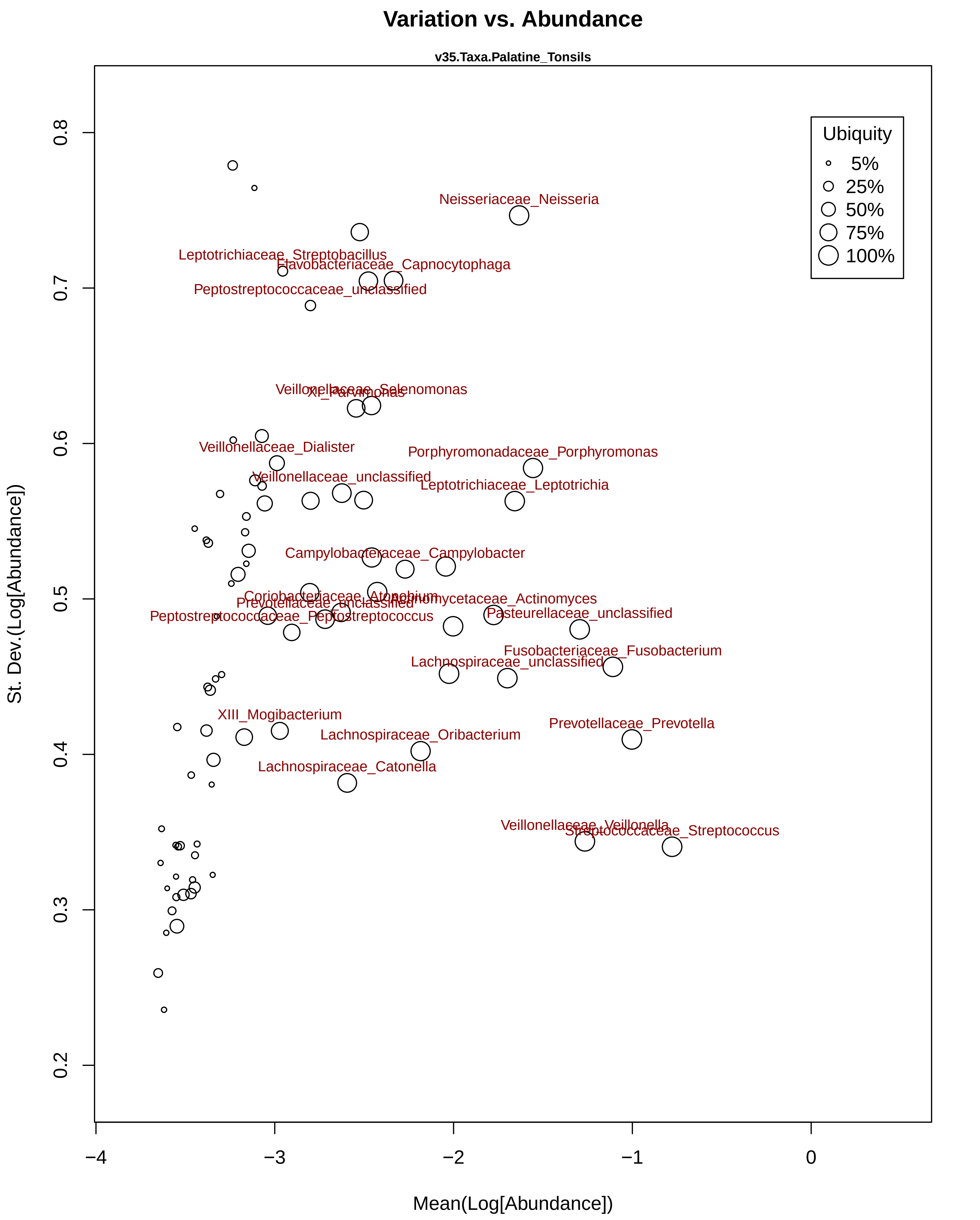
Variation vs. Abundance
v35.Taxa.Palatine_Tonsils
Ubiquity
5%
25%
50%
75%
100%
Neisseriaceae_Neisseria
Leptotrichiaceae_Streptobacillus
Flavobacteriaceae_Capnocytophaga
Peptostreptococcaceae_unclassified
Veillonellaceae_Selenomonas
XI_Parvimonas
Veillonellaceae_Dialister
Porphyromonadaceae_Porphyromonas
Veillonellaceae_unclassified
Leptotrichiaceae_Leptotrichia
Campylobacteraceae_Campylobacter
Coriobacteriaceae_Atopobium
Prevotellaceae_unclassified
Actinomycetaceae_Actinomyces
Peptostreptococcaceae_Peptostreptococcus
Pasteurellaceae_unclassified
Fusobacteriaceae_Fusobacterium
Lachnospiraceae_unclassified
XIII_Mogibacterium
Lachnospiraceae_Oribacterium
Prevotellaceae_Prevotella
Lachnospiraceae_Catonella
Veillonellaceae_Veillonella
Streptococcaceae_Streptococcus
St. Dev.(Log[Abundance])
0.2
0.3
0.4
0.5
0.6
0.7
0.8
Mean(Log[Abundance])
−4
−3
−2
−1
0

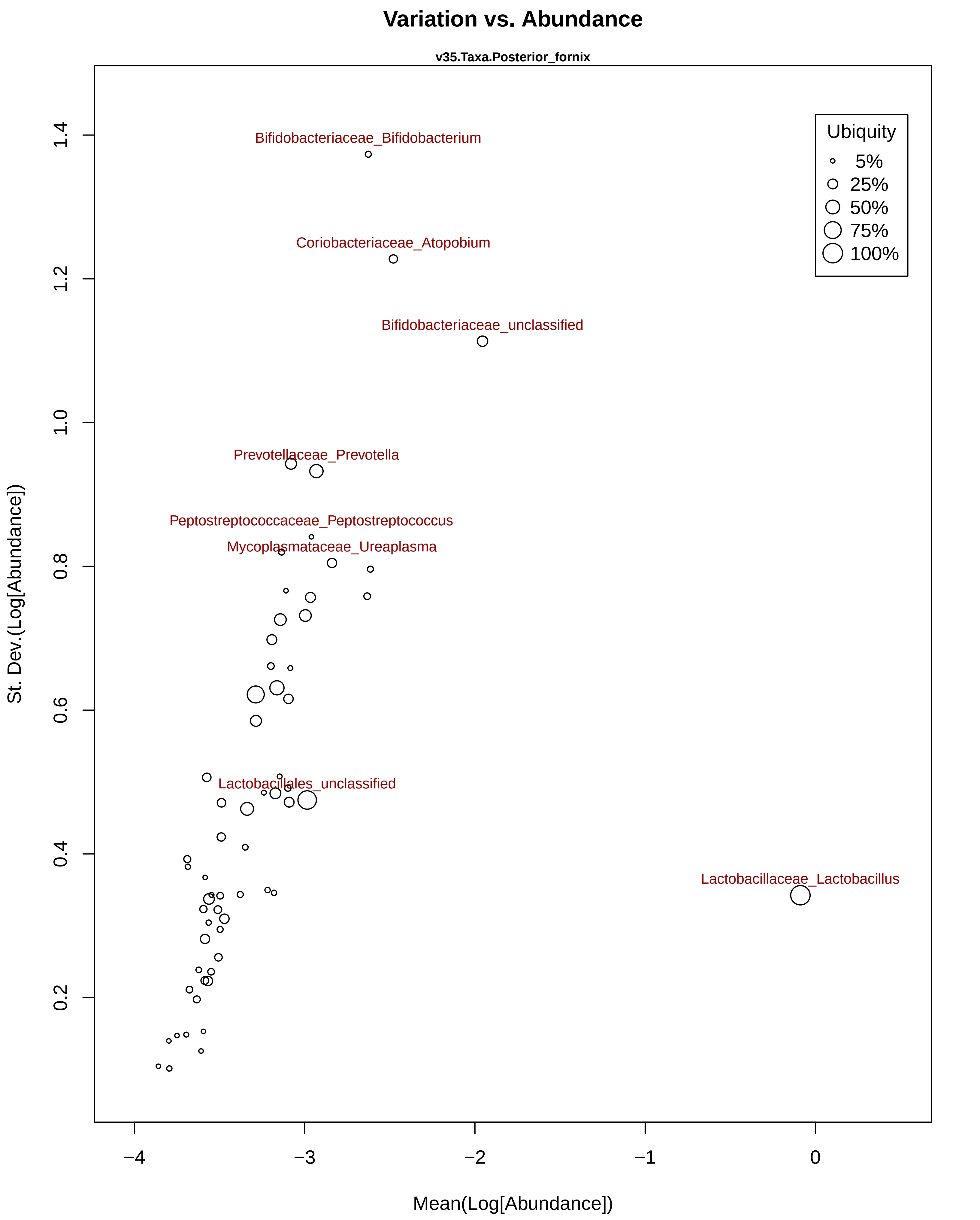

Variation vs. Abundance
v35.Taxa.Posterior_fornix
Ubiquity
5%
25%
50%
75%
100%
Bifidobacteriaceae_Bifidobacterium
Coriobacteriaceae_Atopobium
Bifidobacteriaceae_unclassified
Prevotellaceae_Prevotella
Peptostreptococcaceae_Peptostreptococcus
Mycoplasmataceae_Ureaplasma
Lactobacillales_unclassified
Lactobacillaceae_Lactobacillus
St. Dev.(Log[Abundance])
Mean(Log[Abundance])
1.4
1.2
1.0
0.8
0.6
0.4
0.2
−4
−3
−2
−1
0

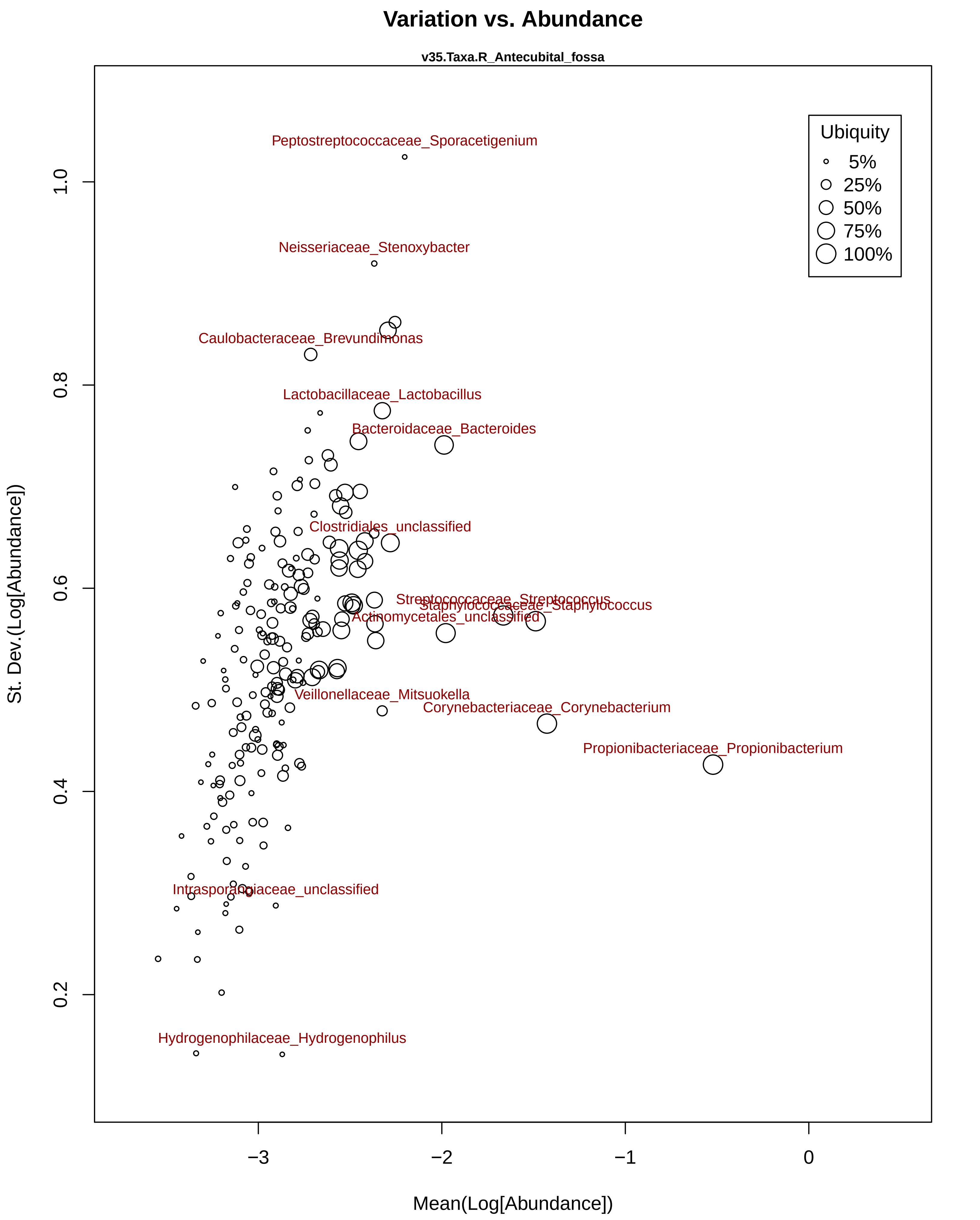

Variation vs. Abundance
v35.Taxa.R_Antecubital_fossa
Ubiquity
5%
25%
50%
75%
100%
Peptostreptococcaceae_Sporacetigenium
Neisseriaceae_Stenoxybacter
Caulobacteraceae_Brevundimonas
Lactobacillaceae_Lactobacillus
Bacteroidaceae_Bacteroides
Clostridiales_unclassified
Streptococcaceae_Streptococcus
Staphylococcaceae_Staphylococcus
Actinomycetales_unclassified
Veillonellaceae_Mitsuokella
Corynebacteriaceae_Corynebacterium
Propionibacteriaceae_Propionibacterium
Intrasporangiaceae_unclassified
Hydrogenophilaceae_Hydrogenophilus
St. Dev.(Log[Abundance])
1.0
0.8
0.6
0.4
0.2
Mean(Log[Abundance])
−3
−2
−1
0

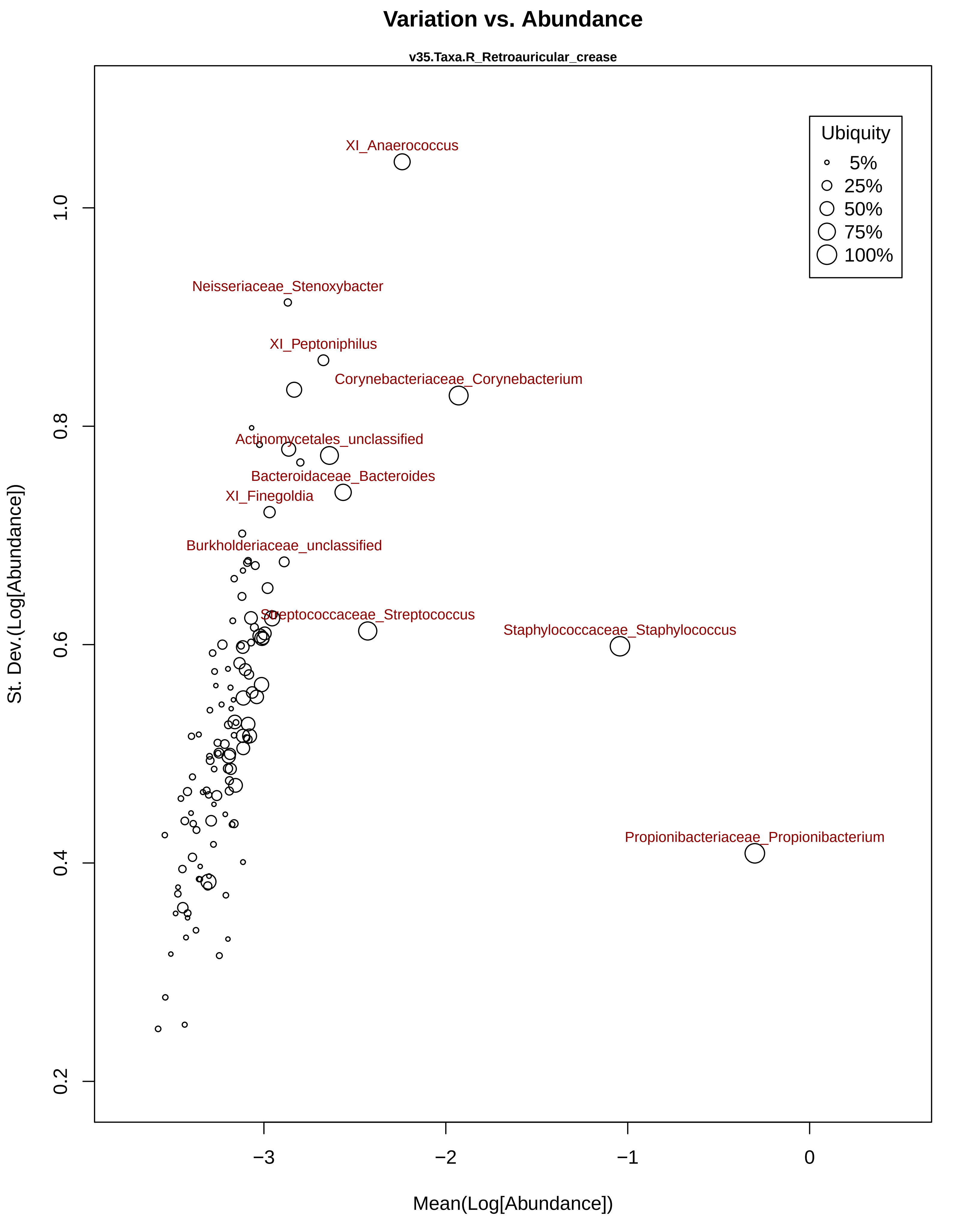
Variation vs. Abundance
v35.Taxa.R_Retroauricular_crease
Ubiquity
5%
25%
50%
75%
100%
XI_Anaerococcus
Neisseriaceae_Stenoxybacter
XI_Peptoniphilus
Corynebacteriaceae_Corynebacterium
Actinomycetales_unclassified
Bacteroidaceae_Bacteroides
XI_Finegoldia
Burkholderiaceae_unclassified
Streptococcaceae_Streptococcus
Staphylococcaceae_Staphylococcus
Propionibacteriaceae_Propionibacterium
St. Dev.(Log[Abundance])
1.0
0.8
0.6
0.4
0.2
−3
−2
−1
0
Mean(Log[Abundance])

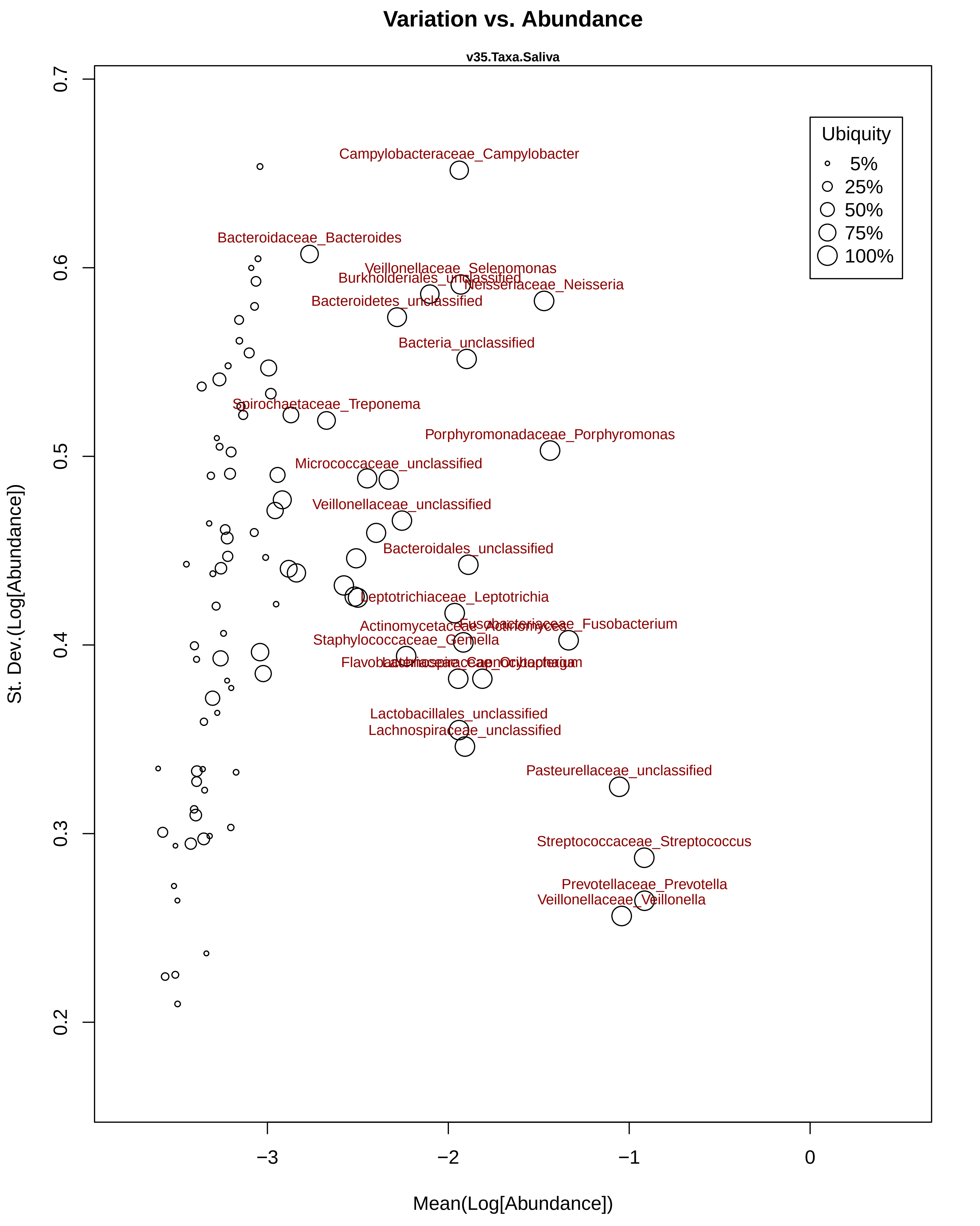

Variation vs. Abundance
v35.Taxa.Saliva
Ubiquity
5%
25%
50%
75%
100%
Campylobacteraceae_Campylobacter
Bacteroidaceae_Bacteroides
Veillonellaceae_Selenomonas
Burkholderiales_unclassified
Neisseriaceae_Neisseria
Bacteroidetes_unclassified
Bacteria_unclassified
Spirochaetaceae_Treponema
Porphyromonadaceae_Porphyromonas
Micrococcaceae_unclassified
Veillonellaceae_unclassified
Bacteroidales_unclassified
Leptotrichiaceae_Leptotrichia
Actinomycetaceae_Actinomyces
Fusobacteriaceae_Fusobacterium
Staphylococcaceae_Gemella
Pasteurellaceae_unclassified
Streptococcaceae_Streptococcus
Prevotellaceae_Prevotella
Veillonellaceae_Veillonella
Lactobacillales_unclassified
Lachnospiraceae_unclassified
0.7
0.6
0.5
0.4
0.3
0.2
−3
−2
−1
0
St. Dev.(Log[Abundance])
Mean(Log[Abundance])

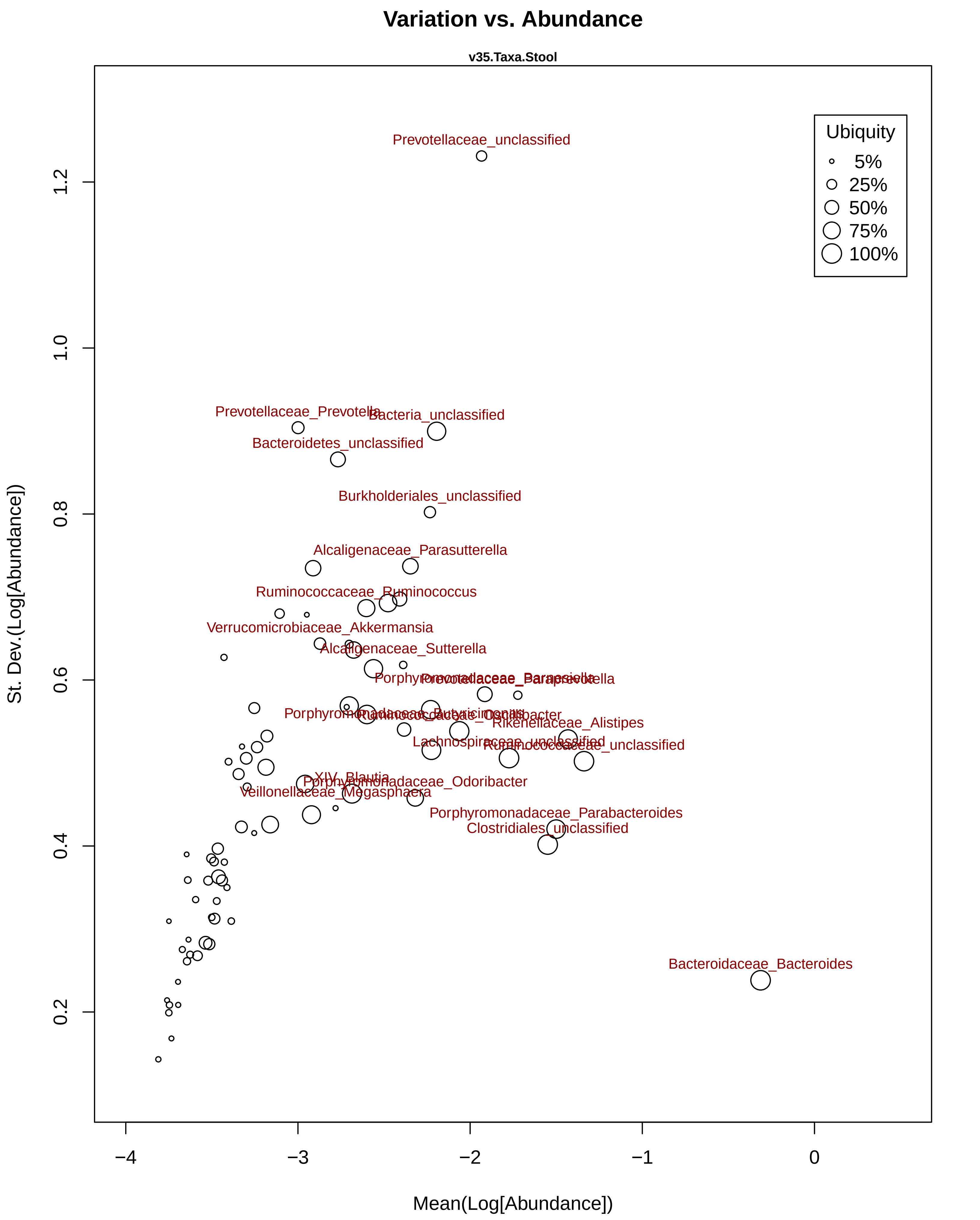

Variation vs. Abundance
v35.Taxa.Stool
Ubiquity
5%
25%
50%
75%
100%
Prevotellaceae_unclassified
Bacteria_unclassified
Bacteroidetes_unclassified
Burkholderiales_unclassified
Alcaligenaceae_Parasutterella
Ruminococcaceae_Ruminococcus
Verrucomicrobiaceae_Akkermansia
Alcaligenaceae_Sutterella
Rikenellaceae_Alistipes
Porphyromonadaceae_Odoribacter
Porphyromonadaceae_Parabacteroides
Clostridiales_unclassified
Bacteroidaceae_Bacteroides
St. Dev.(Log[Abundance])
Mean(Log[Abundance])
0.2
0.4
0.6
0.8
1.0
1.2
−4
−3
−2
−1
0

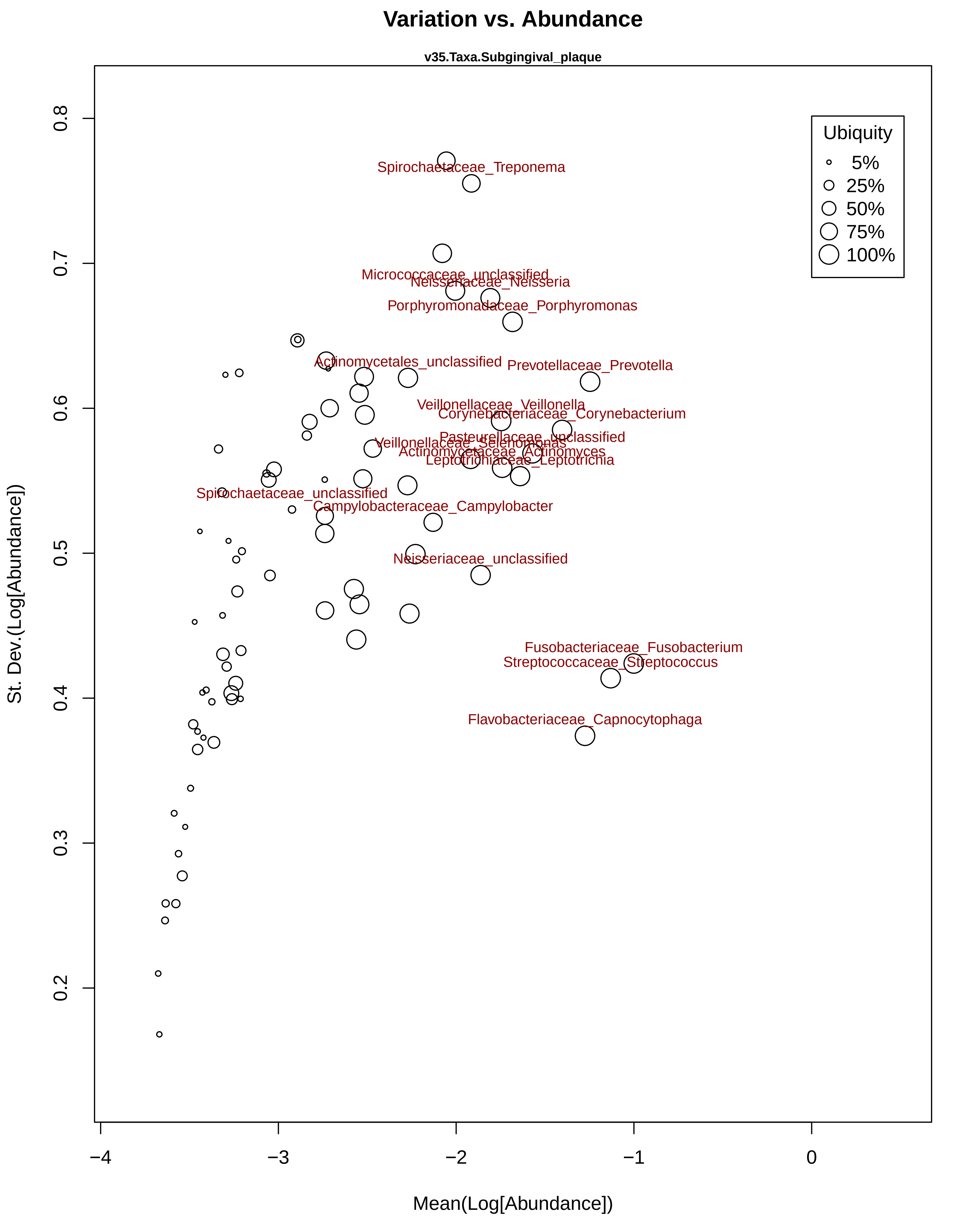
Variation vs. Abundance
v35.Taxa.Subgingival_plaque
Ubiquity
5%
25%
50%
75%
100%
Spirochaetaceae_Treponema
Micrococcaceae_unclassified
Neisseriaceae_Neisseria
Porphyromonadaceae_Porphyromonas
Actinomycetales_unclassified
Prevotellaceae_Prevotella
Veillonellaceae_Veillonella
Corynebacteriaceae_Corynebacterium
Pasteurellaceae_unclassified
Veillonellaceae_Selenomonas
Actinomycetaceae_Actinomyces
Leptotrichiaceae_Leptotrichia
Spirochaetaceae_unclassified
Campylobacteraceae_Campylobacter
Neisseriaceae_unclassified
Fusobacteriaceae_Fusobacterium
Streptococcaceae_Streptococcus
Flavobacteriaceae_Capnocytophaga
St. Dev.(Log[Abundance])
0.8
0.7
0.6
0.5
0.4
0.3
0.2
−4
−3
−2
−1
0
Mean(Log[Abundance])

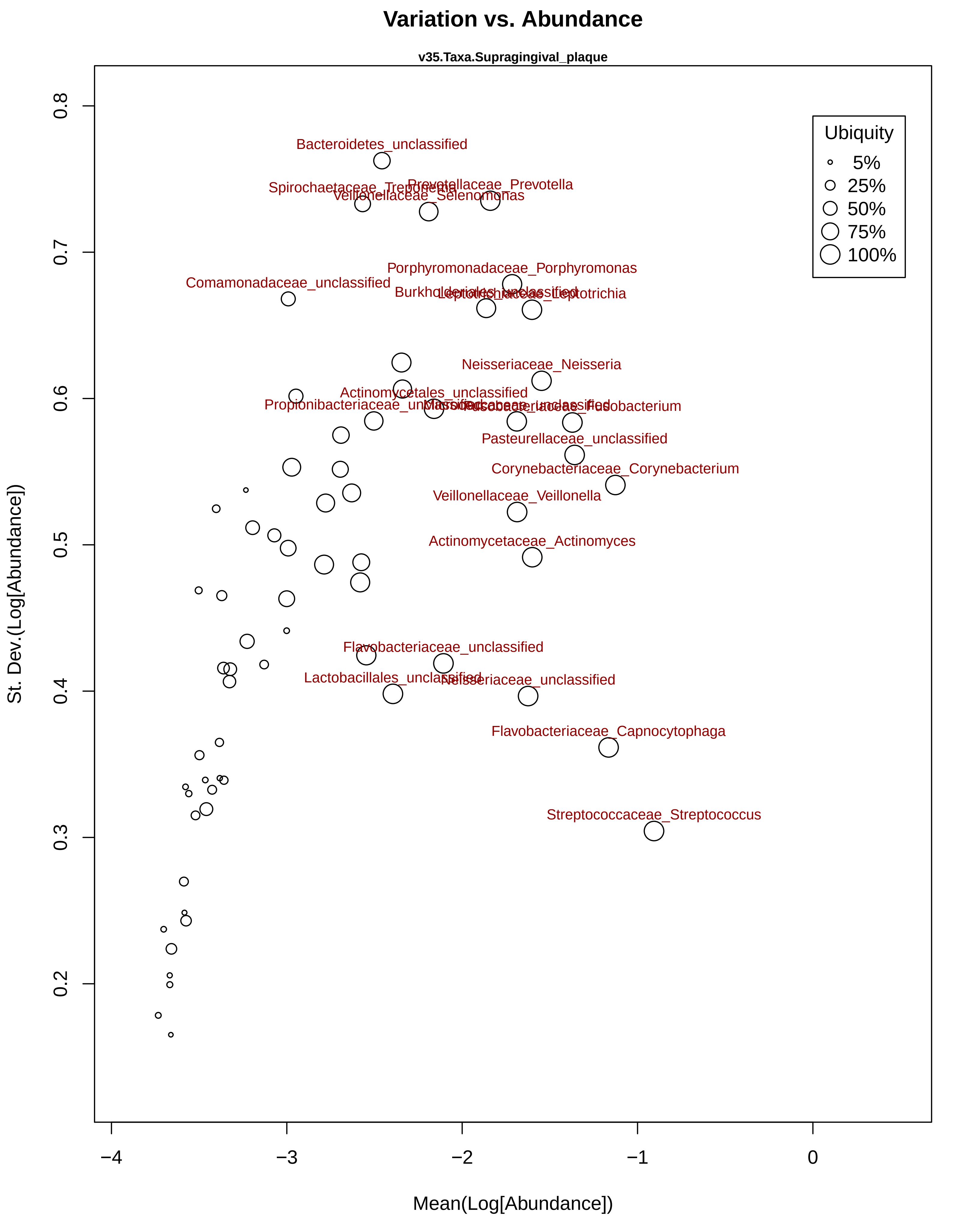

Variation vs. Abundance
v35.Taxa.Supragingival_plaque
Ubiquity
5%
25%
50%
75%
100%
St. Dev.(Log[Abundance])
Mean(Log[Abundance])
0.2
0.3
0.4
0.5
0.6
0.7
0.8
−4
−3
−2
−1
0
Bacteroidetes_unclassified
Spirochaetaceae_Treponema
Prevotellaceae_Prevotella
Veillonellaceae_Selenomonas
Porphyromonadaceae_Porphyromonas
Comamonadaceae_unclassified
Burkholderiales_unclassified
Leptotrichiaceae_Leptotrichia
Neisseriaceae_Neisseria
Actinomycetales_unclassified
Fusobacteriaceae_Fusobacterium
Pasteurellaceae_unclassified
Corynebacteriaceae_Corynebacterium
Veillonellaceae_Veillonella
Actinomycetaceae_Actinomyces
Flavobacteriaceae_unclassified
Lactobacillales_unclassified
Neisseriaceae_unclassified
Flavobacteriaceae_Capnocytophaga
Streptococcaceae_Streptococcus

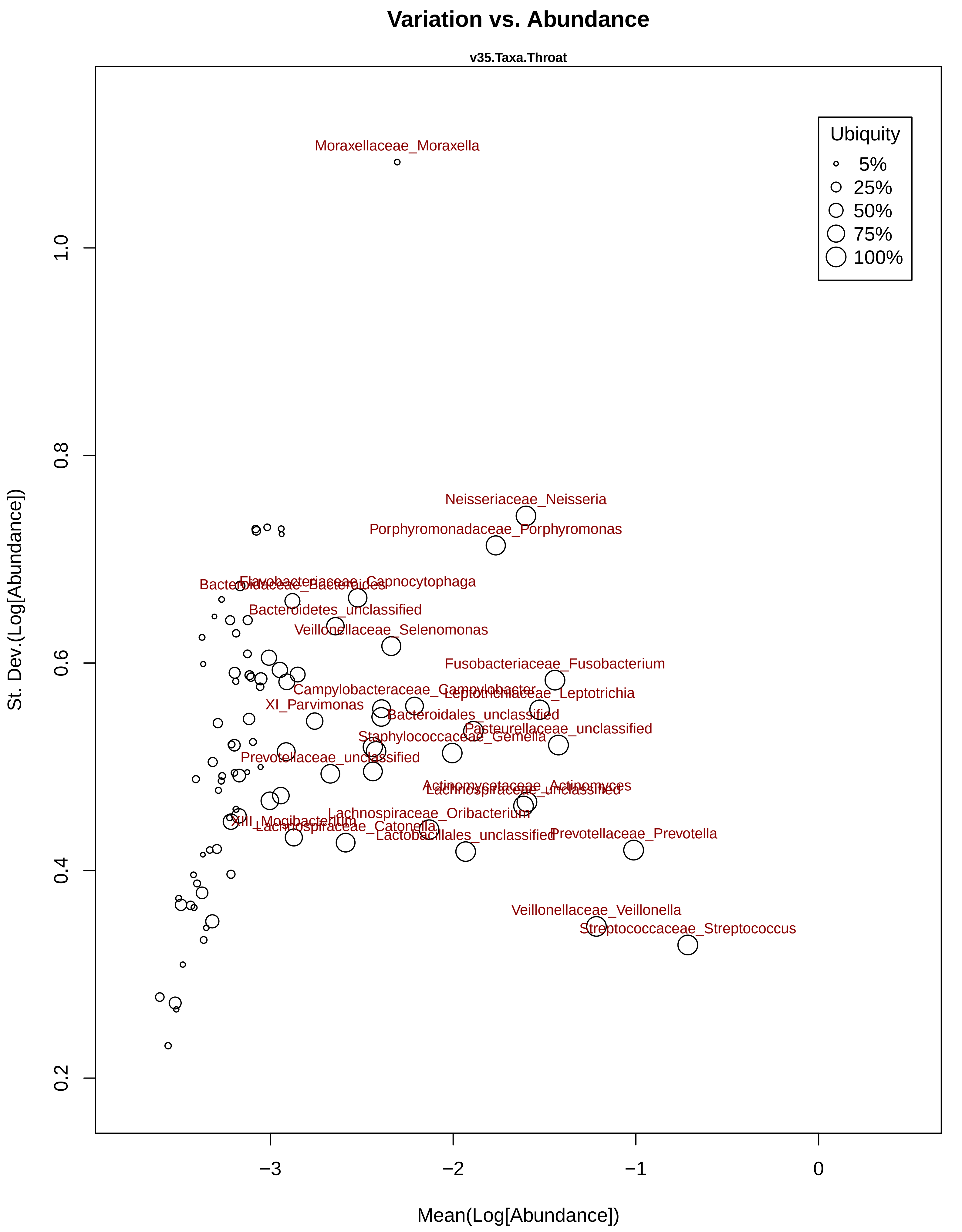
Variation vs. Abundance
v35.Taxa.Throat
Ubiquity
5%
25%
50%
75%
100%
Moraxellaceae_Moraxella
Neisseriaceae_Neisseria
Porphyromonadaceae_Porphyromonas
Flavobacteriaceae_Capnocytophaga
Bacteroidaceae_Bacteroides
Bacteroidetes_unclassified
Veillonellaceae_Selenomonas
Fusobacteriaceae_Fusobacterium
Campylobacteraceae_Campylobacter
Leptotrichiaceae_Leptotrichia
XI_Parvimonas
Bacteroidales_unclassified
Pasteurellaceae_unclassified
Staphylococcaceae_Gemella
Prevotellaceae_unclassified
Actinomycetaceae_Actinomyces
Lachnospiraceae_unclassified
Lachnospiraceae_Oribacterium
XIII_Mogibacterium
Lachnospiraceae_Catonella
Lactobacillales_unclassified
Prevotellaceae_Prevotella
Veillonellaceae_Veillonella
Streptococcaceae_Streptococcus
St. Dev.(Log[Abundance])
Mean(Log[Abundance])
0.2
0.4
0.6
0.8
1.0
−3
−2
−1
0

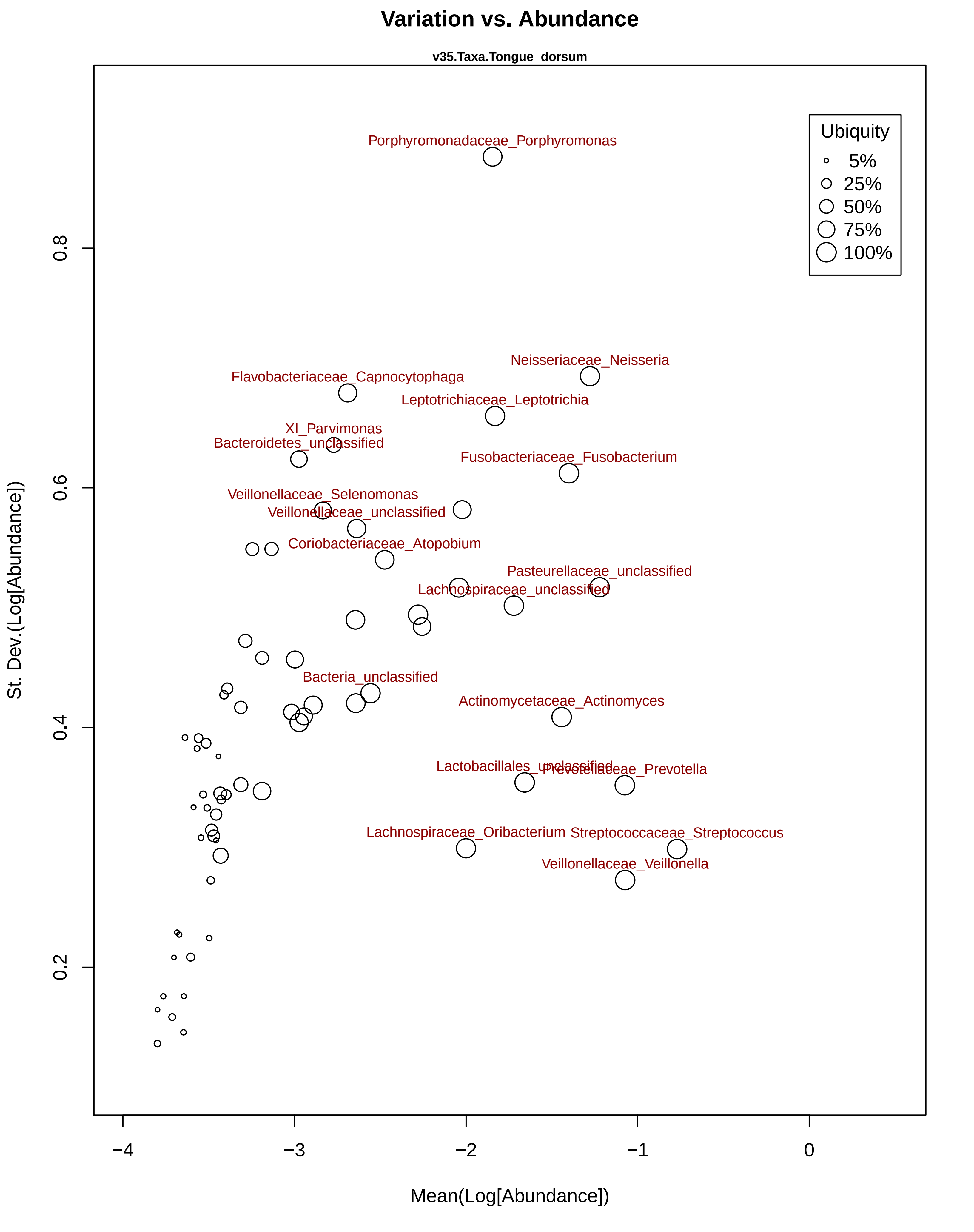
Variation vs. Abundance
v35.Taxa.Tongue_dorsum
Ubiquity
5%
25%
50%
75%
100%
Porphyromonadaceae_Porphyromonas
Neisseriaceae_Neisseria
Flavobacteriaceae_Capnocytophaga
Leptotrichiaceae_Leptotrichia
XI_Parvimonas
Bacteroidetes_unclassified
Fusobacteriaceae_Fusobacterium
Veillonellaceae_Selenomonas
Veillonellaceae_unclassified
Coriobacteriaceae_Atopobium
Pasteurellaceae_unclassified
Lachnospiraceae_unclassified
Bacteria_unclassified
Actinomycetaceae_Actinomyces
Lactobacillales_unclassified
Prevotellaceae_Prevotella
Lachnospiraceae_Oribacterium
Streptococcaceae_Streptococcus
Veillonellaceae_Veillonella
St. Dev.(Log[Abundance])
0.2
0.4
0.6
0.8
Mean(Log[Abundance])
−4
−3
−2
−1
0

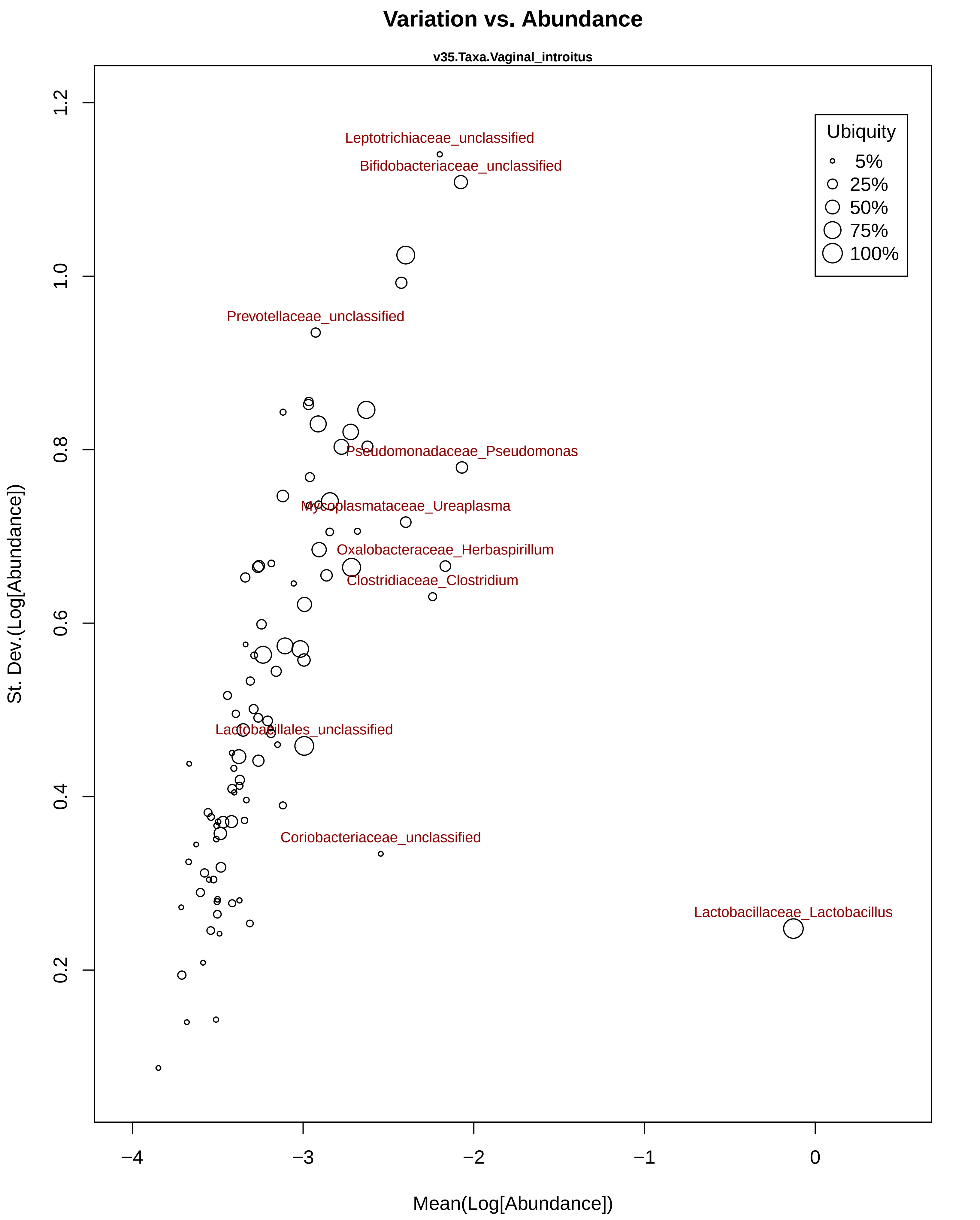

Variation vs. Abundance
v35.Taxa.Vaginal_introitus
Ubiquity
5%
25%
50%
75%
100%
Leptotrichiaceae_unclassified
Bifidobacteriaceae_unclassified
Prevotellaceae_unclassified
Pseudomonadaceae_Pseudomonas
Mycoplasmataceae_Ureaplasma
Oxalobacteraceae_Herbaspirillum
Clostridiaceae_Clostridium
Lactobacillales_unclassified
Coriobacteriaceae_unclassified
Lactobacillaceae_Lactobacillus
St. Dev.(Log[Abundance])
Mean(Log[Abundance])
0.2
0.4
0.6
0.8
1.0
1.2
−4
−3
−2
−1
0
